# Design and performance optimization of vehicle-mounted thermal snow-melting system

**DOI:** 10.1371/journal.pone.0317957

**Published:** 2025-03-13

**Authors:** Zhaoran Li, Yanqing Sheng, Weihan Xu

**Affiliations:** 1 CAS Key Laboratory of Coastal Zone Environmental Processes and Ecological Remediation, Yantai Institute of Coastal Zone Research, Chinese Academy of Sciences, Yantai, China; 2 State Environmental Protection Key Laboratory of Land and Sea Ecological Governance and Systematic Regulation, Shandong Academy for Environmental Planning, Jinan, China; ICIMOD: International Centre for Integrated Mountain Development, NEPAL

## Abstract

Snow clearing and melting vehicles do not need to transport snow out of the country, which helps to alleviate traffic congestion and has broad market prospects. In this study, a vehicle-mounted thermal snow-melting system was designed and the performance of the thermal snow-melting system was simulated. Then, the influences of the entry and exit modes, flow rate of the heat medium, and scraper speed on the snow melting rate were analysed. Studies have shown that thermal snowmelt systems are well designed and have good stability and availability. The speed of the scraper significantly affects the rate of snow melting. As the speed of the scraper increases from 0 r/min to 42 r/min, the time required for complete snow melting was reduced to approximately 1/25 of the original. The flow rate of the thermal medium has a small effect on the rate of snow melting. The time taken for the snow to melt completely under the internal inlet and external outlet method is 1.2 to 2.3 times longer than that of the external inlet and internal outlet method. The rank of influence degree was scraper speed>  entry and exit modes>  flow rate when the target was the maximum melting rate. This research provides a scientific basis for the manufacture of integrated snow clearing and melting equipment.

## Introduction

Snowfall not only purifies the air and replenishes water sources, but also brings many inconveniences to people’s production and life. Slippery roads caused by snow make them dangerous for both vehicles and pedestrians [1,2]. In China, approximately 75% of the country experiences snowfall during the winter months [3]. For this reason, many countries around the world are actively researching and developing technologies for fast and efficient snow removal. Snow salt, mechanical snow removal vehicles, and thermal snow removal are the more common methods of snow removal [4–6]. Among them, snow-melting salt is popular due to its low cost and good snow-melting effect [7,8], with nearly 35 million tons of snow-melting salt used around the world each year [9]. However, the extensive use of snow-melting salt can pollute water and soil, corrode pavement and steel, and cause a series of ecological and environmental problems [10,11]. Snow-melting salt has been banned in some areas. Thermal snow removal is a technique that converts energy input from the outside into heat to melt snow and ice on roadways [12,13]. Common thermal snow-melting methods include the heat-conducting concrete method [14], the circulating pipe heat flow method [15], and the heat-generating cable method [16]. Although this technology is environmentally friendly, it is more costly and technically challenging [17,18]. Therefore, the general municipal roadway snow removal cannot be applied, and is currently mainly used in special areas such as airport runways, bridge surface [19,20].

Mechanical snow removal vehicles have a wide range of applications, fast snow removal speed and high manoeuvrability [21]. However, it is unable to remove snow directly, and snow is usually stacked on the shoulder or sides of the road and then transported to a designated location using large loading and transportation vehicles [22]. This would cause road traffic congestion and significantly increase the cost of mechanical snow removal vehicles. Related studies have shown that the cost of transporting 10 miles of snow was comparable to the fuel cost needed to melt the same amount of snow [23]. In addition, many cities do not have snow dumping sites, and some harbors, rivers, lakes, or other waterways also prohibit snow dumping. Therefore, if integrated snow removal and melting equipment can be manufactured to achieve rapid snow-water conversion, road snow would not need to be stacked and transported out, which would help to alleviate traffic congestion and reduce the cost of mechanical snow removal. From the perspective of energy conservation, heat needs to be supplied to snow to achieve the conversion from snow to water [24]. The efficiency of snow melting is closely related to the method of snow accumulation, the heat exchange process of heat source, and other factors. Through reasonable organization, rapid snow melting can be achieved [25].

At present, the snow melting methods of in situ snow melting equipment can be accessed from the publicly available information are mainly divided into (1) heating the snow into water and then discharging it [26]; (2) using high-pressure sprinklers to spray low-temperature water onto the snow to melt the snow into water [27]. The drawback of melting snow by placing it on a heating device is low snow melting efficiency. The biggest problem with low-temperature water high-pressure spray snow melting is the need for a large amount of low-temperature water, and melted snow water is easy to freeze in the device. There are now many improvements to snow melting equipment. Some manufacturers, such as the Snow Dragon and the Trecan, have successfully developed and launched corresponding snow melting machines for thermal fluid snow melting. The principle of these equipments was to shovel the snow into the snow melting bin and use the heat exchanger tubes in the bin to heat the snow melting, while installing a sprinkler system to spray hot water onto the snow to accelerate its melting. In order to improve the efficiency of snow melting, large quantities of hot water need to be sprayed, which results in high energy consumption and larger equipment volumes. In addition, although these devices are movable, they still need to be placed in designated locations, and require the use of specialized trailers, forklifts, and other equipment. Unlike previous studies, the thermal snow melting system in this study is applied to an integrated snow clearing and melting equipment to realize the purpose of melting the snow while collecting it under the premise of low consumption. In this study, thermal fluid was used as the heat source to build a thermal snow melting system to explore the snow melting process and provide a scientific basis for the manufacture of integrated snow removal and melting equipment. Meanwhile, the influences of the entry and exit modes, flow rate of the heat medium, and contact mode between snow and heat source on the snow melting rate were analysed to optimize the thermal snow melting system.

## Construction of thermal snow-melting systems

**Fig 1:**
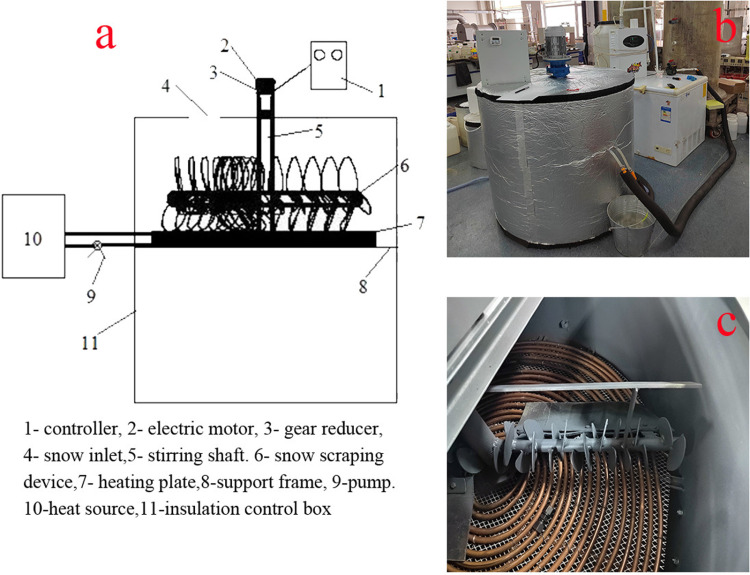
Thermal snow melting system device (a. Schematic diagram of the device, b. Physical diagram of the device, c. Internal diagram of the device).

The thermal snow-melting system mainly consists of an insulated control box, a heating device, and a snow scraping device (Fig 1). After the snow passes through the snow inlet and enters the heat preservation control box, it melts on the heating device under the continuous scraping pressure by the snow scraping device. The heating device includes a heat source, heat medium supply pipeline, heating plate, booster pump and so on. The heating plate is composed of heating tubes arranged spirally in a horizontal plane. The heat transfer capacity of heat pipes plays an important role in snow melting performance [28]. Therefore, we chose copper pipes with better thermal conductivity, ductility, and corrosion resistance to make the heating disk [29].

In the snow melting process, in addition to flowing to the bottom of the heating plate, some of the meltwater soaks into the snow and wets it due to the capillary effect. In this case, a “snow dome” is formed, which reduces the contact area with the heating plate and thus reduces the efficiency of snow melting [25,30]. To solve this issue, a snow scraping device with adjustable speed has been installed in the thermal snow-melting system. The snow scraping device includes a controller, electric motor, gear reducer, stirring shaft, snow scraping frame, scraper blade, etc. There is a clearance between the lowermost part of the snow scraping frame and the surface of the heating plate to accommodate the snow. The scraper blade is tightly attached to the heating plate and inclined at a certain angle (30° ~ 60°) to the heating plate plane. These settings ensure that the snow is evenly distributed on the heating plate while being continuously squeezed downwards, which facilitates full contact between the snow and the heating plate, thus improving snow melting efficiency and energy utilization. In addition, a screen is installed between the heating device and the support frame to prevent snow from falling to the bottom of the thermal control box before melting.

In order to understand the snow melting effectiveness of the designed thermal snow melting system, a test device was designed. This device was a cylinder of Φ1000 mm × 1000 mm, which was made of stainless steel and wrapped with an insulating material on the outside. The scraper was distributed by the “herringbone”, and the rotation direction of the scraper was in the same direction as the spiral direction of the heating tube. This design was designed to realize that during the movement of the scraper, debris, such as tree branches, can be pushed towards the edge of the heating plate and leak through the gap between the heating plate and the insulation manipulation box to its bottom. Debris that entered the bottom of the insulation manipulation box can be discharged through the drain port below. The scraper speed (SS) can be adjusted within 0-50 r/min. The heat source was heated by an electric water heater, with a temperature set at 90°C. The diameter of the heated copper tubes was 7 mm, and the distance between adjacent tubes of the heat pipes was approximately 10 mm. The flow rate of the thermal medium was regulated by a booster pump.

## Performance simulation of the thermal snow melting system

To verify the usability and stability of the designed thermal snowmelt system, the performance of the thermal snowmelt system was simulated by the Solidworks® software package [31]. The main purpose of this simulation is to explore whether the thermal medium can be stably supplied, the movement of the fluid, and whether the melted snow water will refreeze. Therefore, the maximum and minimum values of the heating pipe temperature, fluid flow traces, and box wall temperature were set as the main monitoring indicators for analysis. Before performing the simulations, we assumed the following conditions:

1. The walls of the insulated control box were completely insulated, and the fluids inside the box would not exchange heat with the external environment
2. The effect of thermal radiation was neglected during the model calculations
3. The temperature of the thermal medium in the heating plate was constant.

In terms of the thermodynamic simulation configuration of the heating plate, the thermal medium of the heating pipe was hot water, and the temperature was set at 90°C. The physical properties of the thermal medium were set as the fluid flow rate and the heat transfer between different media. In the computational domain, all fluids except the physical model and the thermal medium were set to 10°C air.

**Fig 2:**
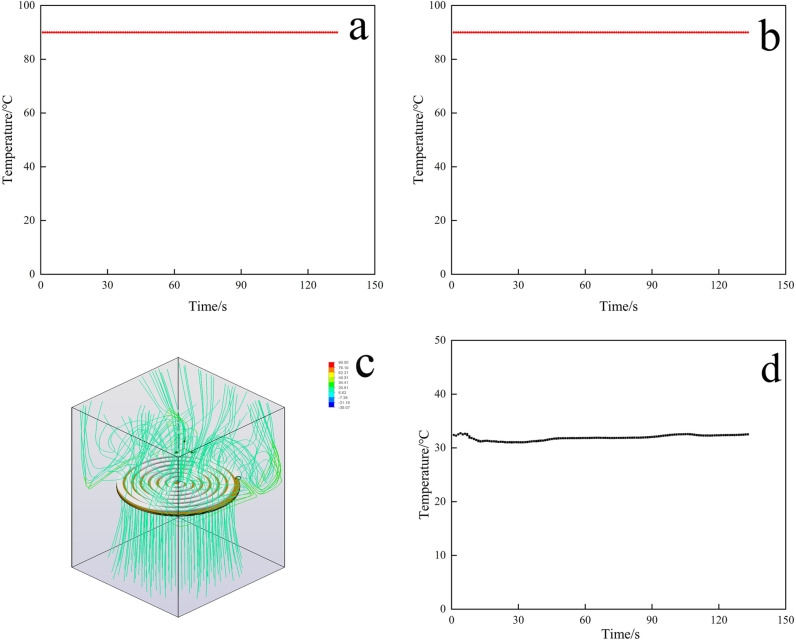
Graph of simulation results (a. Maximum value of heating pipe temperature; b. Minimum value of heating pipe temperature; c. Trajectory of the fluid; d. Wall temperature of the box) (Detailed data in S1 Table).

Fig 2a and Fig 2b show the maximum and minimum values of the heated pipe temperatures. From Fig 2a and Fig 2b, it can be seen that the maximum and minimum values of the temperature of the heated pipe were kept at 90°C, which indicated that this heat source had good anti-interference performance. The analysis of the trajectory of the fluid revealed that the thermal fluid moved downwards in addition to moving upwards (Fig 2c). Therefore, setting an appropriate distance between the heating plate and the sieve can make better use of the heat source. In addition, due to the churning effect of the scraper, the temperature of the thermal fluid at the periphery of the heating plate was significantly higher than that at the center of the heating plate. Therefore, during snow melting, more snow can be laid on the outer layer of the heating plate. In order to explore whether the melted snow water would refreeze, we analysed the temperature of the box wall and found that the wall temperature could be maintained at approximately 32°C, at which point the snow water would not condense into ice (Fig 2d). The above simulations showed that the thermal snow melting system was well designed with good stability and availability for melting snow.

## Optimization of thermal snowmelt systems

The same quality of snow (5 kg) was added to the heating plate, different water inlet and outlet methods, the flow rate of the heat medium, and the speed of the scraper were set up. The specifications of the snow were: (1) the snow density was 100 kg/m^3^, (2) the water content of snow was 0.24% ~ 0.65%, and (3) the temperature of snow was -3 ~ 0°C. The snow melting time, inlet and outlet water temperatures and box temperatures were recorded, and then the appropriate snow melting method was selected. The inlet water temperature, outlet water temperature and box temperature were measured by YET-640X thermometer (YOWEXA, China), with a range of -200°C ~ 1370°C, a measurement accuracy of 0.01°C, and a measurement interval of 1s. The internal inlet and external outlet method was that the thermal medium enters from the center of the plate and exits from the outer layer of the plate. The external inlet and internal outlet method was exactly the opposite. The change of the inlet and outlet method was realized by changing the position of the heat medium entering the heating plate. When the inlet and outlet method was used, the outlet pipe of the heat source was connected to the center position of the heating plate, and the inlet pipe of the heat source was connected to the outer position of the heating plate. The flow rates of thermal medium were 30 L/min, 40 L/min and 60 L/min, respectively. The flow rate of the heat medium was adjusted by the pump. The speed of the scraper was set to 0 r/min, 25 r/min and 42 r/min, respectively. The scraper speed was regulated through the scraper controller.

**Fig 3:**
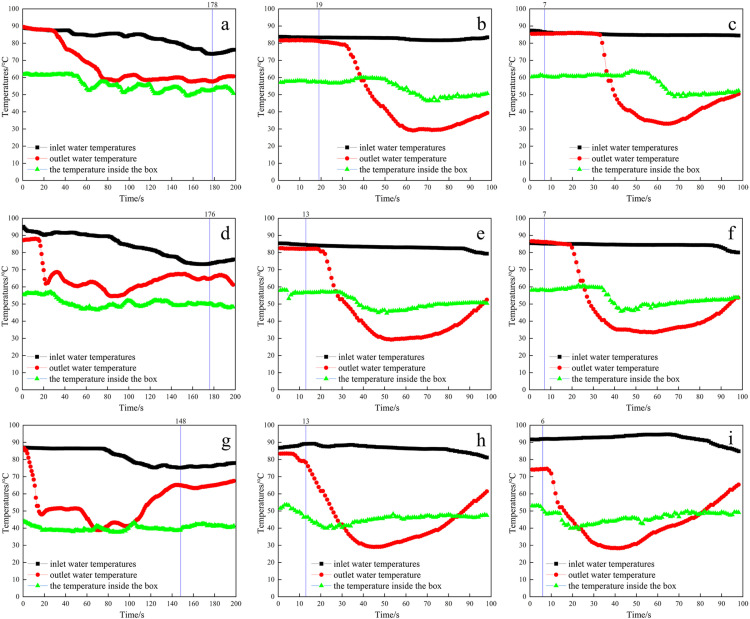
Snow melting effect under the external inlet and internal outlet method with different TMFR, SS, and SCMT, (a) 30 L/min, 0 r/min, 178 s, (b) 30 L/min, 25 r/min, 19 s, (c) 30 L/min, 42 r/min, 7 s, (d) 40 L/min, 0 r/min, 176 s, (e) 40 L/min, 25 r/min, 13 s, (f) 40 L/min, 42 r/min, 7 s, (g) 60 L/min, 0 r/min, 148 s, (h) 60 L/min, 25 r/min, 13 s, (i) 60 L/min, 42 r/min, 6 s (Detailed data in S2 Table).

Fig 3 showed the time of complete melting of snow, inlet and outlet water temperatures, and the temperature inside the box under the influence of different thermal medium flow rates (TMFR) and scraper speeds (SS) under the external inlet and internal outlet method. As shown in Fig 3, the inlet water temperature and the temperature inside the box remained basically constant at the same flow rate and different rotational speeds. It indicated that the different snow-melting efficiencies were mainly caused by different scraper speeds, and it also showed that the heat source was basically able to provide the required heat in a stable manner. When the speed of the scraper increased from 0 r/min to 25 r/min, the snow completely melted time (SCMT) was shortened to approximately 1/10 of the original. However, when the speed of the scraper reached 42 r/min, the time taken for the snow to completely melt was only approximately 1/25 of the original. This indicated that increasing the speed of the scraper significantly reduced the time required for complete melting of the snow.

The melting of snow at the heating plate can be divided into three main phases: the initial stage, rapid stage, and slow stage. During the initial stage, the heating plate was completely covered with snow [1]. In the rapid stage, the melting snow water was lifted into the pores of the snow by capillary forces [32], resulting in enhanced heat transfer [33,34], and thus the snow melting efficiency rapidly increased. In the slow stage, there is a significant decrease in snowmelt efficiency due to the decrease in capillary permeability and the formation of “snow domes” [25]. Increasing the rotational speed of the scraper allowed the snow to be quickly and evenly distributed on the heating plate, and continue to be squeezed downwards. This allows sufficient contact between the snow and the heating plate, avoiding the formation of a “snow dome” and thus shortening the initial and slow phases. Therefore, the snow melting efficiency increased with the increasing scraper speed. Meanwhile, the squeezing of the scraper rotation led to changes in the structure and properties of the snow, resulting in an increase in density and thermal conductivity, which accelerated the melting of the snow [35]. In addition, the rotation of the scraper also caused the heated air to spiral upwards, which was conducive to contact with the snow and promoted the melting of the snow.

From Fig 3, it can be seen that the outlet water temperature has an obvious hysteresis phenomenon, and the outlet water temperature starts to decrease after the snow has completely melted. This was mainly due to the copper pipe was longer, and the thermal medium that exchanged heat with the snow finished flowing to the outlet took some time, at the same time, the rate of snow melting was faster, and the snow finished melting in a very short time. The decrease in temperature of the outlet water in the scraper rotation group was significantly lower than that in the unchurned group (at 0 r/min), which was mainly due to the rapid melting of snow absorbing a large amount of heat in a short period of time, resulting in a sharp decrease in temperature.

Analysis was conducted on the indicators such as the time for complete melting of snow, inlet and outlet water temperatures, and the temperature inside the box under different thermal medium flow rates. It was found that the flow rate of the thermal medium had relatively little effect on the time required for complete melting of snow, the inlet water temperature, and the temperature inside the box, while it had a greater effect on the outlet water temperature. The higher the flow rate of the thermal medium was, the earlier the outlet water temperature started to decrease and increase. Increasing the flow rate of the thermal medium would increase the amount of heat passing through the heating plate per unit of time. The increased heat should lead to accelerated snow melting, but this phenomenon did not occur in this study. This is mainly due to the fact that at a scraper speed of 0 r/min, the increase in heat increased the heat exchange between the snow and the thermal medium, but some of the snow water soaked into the snow and wetted, forming an “ice dome” [25], which separated the snow from the heated plate. The increased heat had less effect on the snow. In the case of stirring, the heat exchange was already more adequate, so the effect of increasing the flow rate of the thermal medium was not obvious.

**Fig 4:**
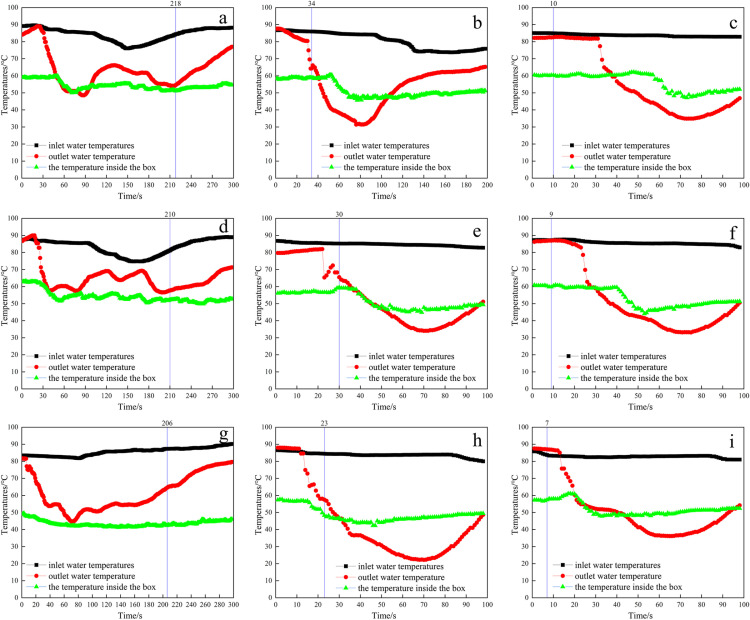
Snow melting effect under the internal inlet and external outlet method with different TMFR, SS, and SCMT, (a) 30 L/min, 0 r/min, 218 s, (b) 30 L/min, 25 r/min, 34 s, (c) 30 L/min, 42 r/min, 10 s, (d) 40 L/min, 0 r/min, 210 s, (e) 40 L/min, 25 r/min, 30 s, (f) 40 L/min, 42 r/min, 9 s, (g) 60 L/min, 0 r/min, 206 s, (h) 60 L/min, 25 r/min, 23 s, (i) 60 L/min, 42 r/min, 7 s (Detailed data in S3 Table).

Fig 4 showed the time of complete melting of snow, inlet and outlet water temperatures, and the temperature inside the box under the influence of different thermal medium flow rates and scraper speeds under the internal inlet and external outlet method. By comparison, it was found that the inlet water temperature and the temperature inside the box remained basically constant at the same flow rate and different rotational speeds. The effects of different thermal medium flow rates and scraper speeds on snow melting were basically the same as those under the external inlet and internal outlet method. When the speed of the scraper increased from 0 r/min to 25 r/min, the snow melting time was shortened to approximately 1/7 of the original. However, when the speed of the scraper reaches 42 r/min, the snow melting time was only approximately 1/25 of the original.

The effect of the water intake method on snow melt was analysed by comparing Fig 3 and Fig 4. From Fig 3 and Fig 4, it can be seen that the effect of the water intake method on the complete melting of snow was more obvious. The time taken for the snow to melt completely under the internal inlet and external outlet method is 1.2 to 2.3 times longer than that of the external inlet and internal outlet method. It is better to melt snow in a way that the thermal medium entered from the outer layer of the plate and flowed out from the center of the plate. This is mainly because to make the branches and other debris fall from the edge of the heating plate, the scraper would produce an outwards force at the same time as the uniform distribution of snow. This prompted the snow and debris to move from the inside to the outside, which caused the snow to melt more in the outer layer of the heating plate. The external inlet and internal outlet method resulted in higher temperatures in the outer layer of the heating plate, which facilitated the melting of snow. However, the internal inlet and external outlet method led to lower temperatures in the outer layers of the heated plate and higher temperatures in the central, resulting in a relatively slower rate of snow melting.

## Advantages, limitations and commercialization potential of the system

The research results indicate that it is technically feasible to develop integrated snow clearing and melting equipment. By analyzing the effects of the inlet and outlet modes, flow rate and scraper speed on the snow-melting efficiency of the thermal snow-melting system, a scientific reference is provided for the practical application of the integrated snow clearing and melting equipment. In practical on-board snow-melting applications, thermal snow-melting system is used in conjunction with other systems such as snow collection and throwing devices, snow water throwing devices and so on. These systems work cooperatively to realize the entire treatment process from snow collection to snowmelt and drainage.

Although this study has achieved preliminary results in exploring the feasibility of synchronous snow removal and melting, there are still some limitations in the research design and results: (1) the limited size of the experiment restricts the generalization and applicability of the results to a certain extent. (2) The simulation assumption is relatively simple, which to some extent affects the accuracy of the simulation results. (3) The environmental adaptability assessment is insufficient. To address the above limitations, future research can focus on the following aspects: (1) expanding the experimental scale to simulate environmental conditions closer to real-world applications, in order to comprehensively evaluate the system’s performance and reliability. (2) Optimizing the simulation model, complex physical processes such as thermal radiation are gradually introduced, in order to improve the accuracy of the simulation. (3) Conduct experiments under different climatic conditions to evaluate the environmental adaptability of the system.

Compared with other existing snow melting technologies, the thermal snow melting system in this study has the following advantages: (1) rapid snow-water conversion. The thermal snow-melting system can directly melt snow on site, without the need for stockpiling or long-distance transportation. The melted snow water can be directly drained off or used to wash the residual snow on the road surface and sidewalks along the road according to the demand. (2) Easy to transport, fix, and operate. The system adopts a modular design, making it convenient for handling, combining, and relocating at any time. Additionally, the system can be scaled up or down according to snow removal tasks or road characteristics, enhancing its flexibility and adaptability. (3) Environmentally friendly. The system does not use snow melting agents, avoiding environmental pollution issues and meeting the requirements of sustainable development. Meanwhile, there are also some issues with this system: (1) Power supply and energy consumption. The system needs to use electricity, or other sources, to provide a heat source for melting snow, so the stability of the power supply needs to be considered. Moreover, the energy consumption of the system is relatively high due to the need for heating to melt the snow. (2) System maintenance and inspection. As the system involves multiple components and equipment (such as heating plates, pipelines, pumps), regular maintenance and inspection of the system are required to ensure its normal operation.

The thermal snow-melting system has shown great advantages in scalability and commercial potential. In terms of scalability: (1) the modular design of the thermal snow-melting system means that the system can be flexibly configured and expanded according to actual needs. (2) The thermal snow-melting system can be equipped with an intelligent control system. The intelligent control system can automatically adjust the heating power based on real-time temperature, humidity, and snow accumulation to achieve efficient energy utilization. In terms of commercial potential: (1) Growing market demand. With global climate change and the frequency of extreme weather events, the amount and frequency of snowfall in winter are increasing, leading to continued growth in market demand. (2) Policy support. Many countries and regions have introduced relevant policies to encourage the use of environmentally friendly and efficient snow melting technology. This provides strong policy support for the promotion and application of thermal snow-melting systems, reducing the difficulty of market promotion. (3) Environmental advantages. Compared with traditional snow clearing and melting methods, the thermal snow melting system has significant environmental advantages. This environmental advantage makes the thermal snow-melting system has a broad application prospect.

## Conclusion

In this study, a vehicle-mounted thermal snow-melting system was designed. The performance of the thermal snow-melting system was simulated using the Solidworks® software package and it was found that the thermal snow-melting system was well-designed with good stability and availability. By analyzing the effects of thermal medium entry and exit modes, flow rate, and scraper rotation speed on the snow melting rate in the thermal snow melting system, it was found that the scraper rotation speed has the greatest effect on the snow melting rate, followed by entry and exit modes, and the thermal medium flow rate has the smallest effect. Appropriately increasing the flow rate of the thermal medium, selecting a higher speed of the scraper, and using external inlet and internal outlet method are conducive to the rapid melting of snow. Although this study has achieved preliminary results in exploring the feasibility of simultaneous snow removal and melting, there are still some limitations. Follow-up research can be carried out in expanding the experimental scale, optimizing simulation models, and evaluating different environmental adaptability.

## Supporting information

S1 Table Fig 2 data. (DOCX)

S2 Table Fig 3 data. (DOCX)

S3 Table Fig 4 data. (DOCX)
