# Supplementary material for: Design and performance optimization of vehicle-mounted thermal snow-melting system
Source: PLoS One. 2025 Mar 13;20(3):e0317957. doi: 10.1371/journal.pone.0317957 (PMC11906039; doi:10.1371/journal.pone.0317957)
Supplement: S1 Table — (DOCX) [file pone.0317957.s001.docx]

**S1 Table. Fig 2 data.**

| Time/s | Maximum value of heating pipe /℃ | Minimum value of heating pipe/℃ | Wall temperature of the box/℃ |
| --- | --- | --- | --- |
| 1 | 90 | 90 | 32.40552 |
| 2 | 90 | 90 | 32.30205 |
| 3 | 90 | 90 | 32.53661 |
| 4 | 90 | 90 | 32.73718 |
| 5 | 90 | 90 | 32.53838 |
| 5 | 90 | 90 | 32.52334 |
| 6 | 90 | 90 | 32.63669 |
| 6 | 90 | 90 | 32.63509 |
| 7 | 90 | 90 | 32.58533 |
| 7 | 90 | 90 | 32.38646 |
| 8 | 90 | 90 | 32.15115 |
| 8 | 90 | 90 | 31.92466 |
| 9 | 90 | 90 | 31.84195 |
| 10 | 90 | 90 | 31.69868 |
| 10 | 90 | 90 | 31.67153 |
| 11 | 90 | 90 | 31.50739 |
| 11 | 90 | 90 | 31.48973 |
| 12 | 90 | 90 | 31.36995 |
| 12 | 90 | 90 | 31.28097 |
| 13 | 90 | 90 | 31.24754 |
| 13 | 90 | 90 | 31.20688 |
| 14 | 90 | 90 | 31.24644 |
| 14 | 90 | 90 | 31.29308 |
| 15 | 90 | 90 | 31.3247 |
| 16 | 90 | 90 | 31.33961 |
| 16 | 90 | 90 | 31.32149 |
| 17 | 90 | 90 | 31.28935 |
| 17 | 90 | 90 | 31.25698 |
| 18 | 90 | 90 | 31.236 |
| 18 | 90 | 90 | 31.22101 |
| 19 | 90 | 90 | 31.2282 |
| 20 | 90 | 90 | 31.21881 |
| 20 | 90 | 90 | 31.18 |
| 21 | 90 | 90 | 31.13401 |
| 21 | 90 | 90 | 31.13404 |
| 22 | 90 | 90 | 31.14925 |
| 22 | 90 | 90 | 31.14412 |
| 23 | 90 | 90 | 31.13606 |
| 24 | 90 | 90 | 31.09943 |
| 24 | 90 | 90 | 31.05735 |
| 25 | 90 | 90 | 31.04911 |
| 25 | 90 | 90 | 31.05377 |
| 26 | 90 | 90 | 31.06731 |
| 26 | 90 | 90 | 31.07878 |
| 27 | 90 | 90 | 31.07609 |
| 27 | 90 | 90 | 31.02884 |
| 28 | 90 | 90 | 31.06813 |
| 29 | 90 | 90 | 31.06649 |
| 29 | 90 | 90 | 31.07612 |
| 30 | 90 | 90 | 31.05717 |
| 30 | 90 | 90 | 31.06669 |
| 31 | 90 | 90 | 31.04792 |
| 31 | 90 | 90 | 31.06795 |
| 32 | 90 | 90 | 31.05422 |
| 32 | 90 | 90 | 31.07101 |
| 33 | 90 | 90 | 31.07348 |
| 34 | 90 | 90 | 31.12034 |
| 34 | 90 | 90 | 31.11293 |
| 35 | 90 | 90 | 31.1621 |
| 35 | 90 | 90 | 31.17272 |
| 36 | 90 | 90 | 31.22288 |
| 36 | 90 | 90 | 31.22676 |
| 37 | 90 | 90 | 31.25851 |
| 38 | 90 | 90 | 31.23819 |
| 38 | 90 | 90 | 31.29631 |
| 39 | 90 | 90 | 31.28741 |
| 39 | 90 | 90 | 31.33535 |
| 40 | 90 | 90 | 31.33051 |
| 40 | 90 | 90 | 31.3805 |
| 41 | 90 | 90 | 31.38185 |
| 42 | 90 | 90 | 31.43725 |
| 42 | 90 | 90 | 31.44846 |
| 43 | 90 | 90 | 31.52231 |
| 43 | 90 | 90 | 31.54058 |
| 44 | 90 | 90 | 31.59498 |
| 44 | 90 | 90 | 31.61388 |
| 45 | 90 | 90 | 31.6727 |
| 45 | 90 | 90 | 31.67169 |
| 46 | 90 | 90 | 31.73695 |
| 47 | 90 | 90 | 31.7318 |
| 47 | 90 | 90 | 31.77495 |
| 48 | 90 | 90 | 31.775 |
| 48 | 90 | 90 | 31.81044 |
| 49 | 90 | 90 | 31.80546 |
| 50 | 90 | 90 | 31.81945 |
| 50 | 90 | 90 | 31.81002 |
| 51 | 90 | 90 | 31.81905 |
| 51 | 90 | 90 | 31.81205 |
| 52 | 90 | 90 | 31.81917 |
| 52 | 90 | 90 | 31.81372 |
| 53 | 90 | 90 | 31.82551 |
| 54 | 90 | 90 | 31.8232 |
| 54 | 90 | 90 | 31.84037 |
| 55 | 90 | 90 | 31.83253 |
| 55 | 90 | 90 | 31.84293 |
| 56 | 90 | 90 | 31.83299 |
| 57 | 90 | 90 | 31.83818 |
| 57 | 90 | 90 | 31.83913 |
| 58 | 90 | 90 | 31.84206 |
| 58 | 90 | 90 | 31.84644 |
| 59 | 90 | 90 | 31.85109 |
| 60 | 90 | 90 | 31.84976 |
| 60 | 90 | 90 | 31.85604 |
| 61 | 90 | 90 | 31.85943 |
| 62 | 90 | 90 | 31.86203 |
| 62 | 90 | 90 | 31.87188 |
| 63 | 90 | 90 | 31.87795 |
| 63 | 90 | 90 | 31.88822 |
| 64 | 90 | 90 | 31.88941 |
| 65 | 90 | 90 | 31.89437 |
| 65 | 90 | 90 | 31.89753 |
| 66 | 90 | 90 | 31.90387 |
| 66 | 90 | 90 | 31.89363 |
| 67 | 90 | 90 | 31.88992 |
| 67 | 90 | 90 | 31.8875 |
| 68 | 90 | 90 | 31.8834 |
| 69 | 90 | 90 | 31.87811 |
| 69 | 90 | 90 | 31.87754 |
| 70 | 90 | 90 | 31.8668 |
| 70 | 90 | 90 | 31.86674 |
| 71 | 90 | 90 | 31.85891 |
| 71 | 90 | 90 | 31.8599 |
| 72 | 90 | 90 | 31.8554 |
| 72 | 90 | 90 | 31.85705 |
| 73 | 90 | 90 | 31.85714 |
| 74 | 90 | 90 | 31.86762 |
| 74 | 90 | 90 | 31.86669 |
| 75 | 90 | 90 | 31.86762 |
| 75 | 90 | 90 | 31.87379 |
| 76 | 90 | 90 | 31.87383 |
| 77 | 90 | 90 | 31.88863 |
| 77 | 90 | 90 | 31.8781 |
| 78 | 90 | 90 | 31.89064 |
| 78 | 90 | 90 | 31.88691 |
| 79 | 90 | 90 | 31.89885 |
| 79 | 90 | 90 | 31.89357 |
| 80 | 90 | 90 | 31.90243 |
| 81 | 90 | 90 | 31.8989 |
| 81 | 90 | 90 | 31.90689 |
| 82 | 90 | 90 | 31.90639 |
| 82 | 90 | 90 | 31.92278 |
| 83 | 90 | 90 | 31.9142 |
| 84 | 90 | 90 | 31.91926 |
| 84 | 90 | 90 | 31.91614 |
| 85 | 90 | 90 | 31.9303 |
| 85 | 90 | 90 | 31.93985 |
| 86 | 90 | 90 | 31.94254 |
| 86 | 90 | 90 | 31.9533 |
| 87 | 90 | 90 | 31.96531 |
| 88 | 90 | 90 | 31.97012 |
| 88 | 90 | 90 | 31.99241 |
| 89 | 90 | 90 | 32.01674 |
| 89 | 90 | 90 | 32.0197 |
| 90 | 90 | 90 | 32.05052 |
| 91 | 90 | 90 | 32.06819 |
| 91 | 90 | 90 | 32.09101 |
| 92 | 90 | 90 | 32.12499 |
| 93 | 90 | 90 | 32.15892 |
| 93 | 90 | 90 | 32.17948 |
| 94 | 90 | 90 | 32.22692 |
| 94 | 90 | 90 | 32.23097 |
| 95 | 90 | 90 | 32.26583 |
| 95 | 90 | 90 | 32.28906 |
| 96 | 90 | 90 | 32.33017 |
| 97 | 90 | 90 | 32.33845 |
| 97 | 90 | 90 | 32.37736 |
| 98 | 90 | 90 | 32.39207 |
| 98 | 90 | 90 | 32.43199 |
| 99 | 90 | 90 | 32.44444 |
| 100 | 90 | 90 | 32.47302 |
| 100 | 90 | 90 | 32.48932 |
| 101 | 90 | 90 | 32.5111 |
| 101 | 90 | 90 | 32.52733 |
| 102 | 90 | 90 | 32.53406 |
| 102 | 90 | 90 | 32.5372 |
| 103 | 90 | 90 | 32.53765 |
| 103 | 90 | 90 | 32.54301 |
| 104 | 90 | 90 | 32.55219 |
| 105 | 90 | 90 | 32.55451 |
| 105 | 90 | 90 | 32.57006 |
| 106 | 90 | 90 | 32.58266 |
| 106 | 90 | 90 | 32.58127 |
| 107 | 90 | 90 | 32.5702 |
| 107 | 90 | 90 | 32.5383 |
| 108 | 90 | 90 | 32.52214 |
| 109 | 90 | 90 | 32.47851 |
| 109 | 90 | 90 | 32.44721 |
| 110 | 90 | 90 | 32.41516 |
| 110 | 90 | 90 | 32.39105 |
| 111 | 90 | 90 | 32.36804 |
| 111 | 90 | 90 | 32.35772 |
| 112 | 90 | 90 | 32.33107 |
| 113 | 90 | 90 | 32.32097 |
| 113 | 90 | 90 | 32.31162 |
| 114 | 90 | 90 | 32.32805 |
| 114 | 90 | 90 | 32.31019 |
| 115 | 90 | 90 | 32.32015 |
| 115 | 90 | 90 | 32.3041 |
| 116 | 90 | 90 | 32.31393 |
| 116 | 90 | 90 | 32.30205 |
| 117 | 90 | 90 | 32.32726 |
| 118 | 90 | 90 | 32.33259 |
| 118 | 90 | 90 | 32.3429 |
| 119 | 90 | 90 | 32.34747 |
| 120 | 90 | 90 | 32.35906 |
| 120 | 90 | 90 | 32.35823 |
| 121 | 90 | 90 | 32.37288 |
| 121 | 90 | 90 | 32.37346 |
| 122 | 90 | 90 | 32.38405 |
| 123 | 90 | 90 | 32.38094 |
| 123 | 90 | 90 | 32.39511 |
| 124 | 90 | 90 | 32.39469 |
| 124 | 90 | 90 | 32.39554 |
| 125 | 90 | 90 | 32.39769 |
| 125 | 90 | 90 | 32.39921 |
| 126 | 90 | 90 | 32.40409 |
| 127 | 90 | 90 | 32.40051 |
| 127 | 90 | 90 | 32.40532 |
| 128 | 90 | 90 | 32.41633 |
| 128 | 90 | 90 | 32.4225 |
| 129 | 90 | 90 | 32.42914 |
| 129 | 90 | 90 | 32.44776 |
| 130 | 90 | 90 | 32.45762 |
| 131 | 90 | 90 | 32.47103 |
| 131 | 90 | 90 | 32.4871 |
| 132 | 90 | 90 | 32.5032 |
| 132 | 90 | 90 | 32.51579 |
| 133 | 90 | 90 | 32.53661 |
