# Supplementary material for: Design and performance optimization of vehicle-mounted thermal snow-melting system
Source: PLoS One. 2025 Mar 13;20(3):e0317957. doi: 10.1371/journal.pone.0317957 (PMC11906039; doi:10.1371/journal.pone.0317957)
Supplement: S2 Table — (DOCX) [file pone.0317957.s002.docx]

**S2 Table. Fig 3 data.**

|  | Time/s | | Inlet water/℃ | | Outlet water/℃ | | Inside the box/℃ | | | Time/s | | Inlet water /℃ | | Outlet water/℃ | | Inside the box/℃ | |  |
| --- | --- | --- | --- | --- | --- | --- | --- | --- | --- | --- | --- | --- | --- | --- | --- | --- | --- | --- |
| a | 1 | | 89.02 | | 89.46 | | 61.75 | | | 100 | | 84.64 | | 61.13 | | 52.76 | |  |
|  | 2 | | 88.94 | | 89.24 | | 61.85 | | | 101 | | 84.69 | | 61.3 | | 52.76 | |  |
|  | 3 | | 88.72 | | 89.04 | | 62.07 | | | 102 | | 84.75 | | 61.31 | | 53.16 | |  |
|  | 4 | | 88.68 | | 88.96 | | 62.07 | | | 103 | | 84.84 | | 61.47 | | 53.77 | |  |
|  | 5 | | 88.64 | | 88.85 | | 62.05 | | | 104 | | 84.9 | | 61.58 | | 54.03 | |  |
|  | 6 | | 88.57 | | 88.64 | | 61.92 | | | 105 | | 85.21 | | 61.45 | | 54.44 | |  |
|  | 7 | | 88.51 | | 88.64 | | 61.65 | | | 106 | | 85.21 | | 61.45 | | 54.78 | |  |
|  | 8 | | 88.45 | | 88.54 | | 61.42 | | | 107 | | 85.24 | | 61.11 | | 55.56 | |  |
|  | 9 | | 88.37 | | 88.25 | | 61.26 | | | 108 | | 85.27 | | 60.74 | | 56.08 | |  |
|  | 10 | | 88.31 | | 88.25 | | 61.46 | | | 109 | | 85.27 | | 60.24 | | 55.92 | |  |
|  | 11 | | 88.24 | | 88.15 | | 61.46 | | | 110 | | 85.23 | | 59.83 | | 55.92 | |  |
|  | 12 | | 88.17 | | 88.23 | | 61.89 | | | 111 | | 85.15 | | 59.44 | | 55.71 | |  |
|  | 13 | | 88.08 | | 87.97 | | 61.89 | | | 112 | | 85.07 | | 59.00 | | 55.58 | |  |
|  | 14 | | 88.02 | | 88.10 | | 61.55 | | | 113 | | 84.83 | | 58.88 | | 55.27 | |  |
|  | 15 | | 87.95 | | 87.96 | | 61.28 | | | 114 | | 84.79 | | 58.63 | | 55.24 | |  |
|  | 16 | | 87.90 | | 87.96 | | 61.29 | | | 115 | | 84.41 | | 58.63 | | 55.08 | |  |
|  | 17 | | 87.88 | | 88.05 | | 61.64 | | | 116 | | 84.08 | | 58.49 | | 55.29 | |  |
|  | 18 | | 87.84 | | 87.96 | | 61.64 | | | 117 | | 83.75 | | 58.59 | | 55.88 | |  |
|  | 19 | | 87.82 | | 87.93 | | 61.64 | | | 118 | | 83.41 | | 58.59 | | 55.88 | |  |
|  | 20 | | 87.78 | | 87.84 | | 61.25 | | | 119 | | 83.37 | | 58.76 | | 56.13 | |  |
|  | 21 | | 87.73 | | 87.82 | | 61.35 | | | 120 | | 83.05 | | 58.61 | | 55.54 | |  |
|  | 22 | | 87.69 | | 87.87 | | 61.51 | | | 121 | | 82.98 | | 58.61 | | 54.15 | |  |
|  | 23 | | 87.63 | | 87.78 | | 61.52 | | | 122 | | 82.72 | | 58.90 | | 52.90 | |  |
|  | 24 | | 87.56 | | 87.80 | | 61.49 | | | 123 | | 82.65 | | 58.85 | | 51.88 | |  |
|  | 25 | | 87.47 | | 87.80 | | 61.28 | | | 124 | | 82.57 | | 58.85 | | 50.62 | |  |
|  | 26 | | 87.47 | | 87.61 | | 61.49 | | | 125 | | 82.5 | | 58.9 | | 50.77 | |  |
|  | 27 | | 87.43 | | 87.62 | | 61.49 | | | 126 | | 82.46 | | 58.87 | | 50.57 | |  |
|  | 28 | | 87.42 | | 87.35 | | 61.78 | | | 127 | | 82.47 | | 58.89 | | 50.57 | |  |
|  | 29 | | 87.44 | | 87.13 | | 61.77 | | | 128 | | 82.46 | | 59.06 | | 50.91 | |  |
|  | 30 | | 87.46 | | 86.74 | | 61.63 | | | 129 | | 82.46 | | 59.28 | | 51.09 | |  |
|  | 31 | | 87.47 | | 86.25 | | 61.62 | | | 130 | | 82.48 | | 59.2 | | 51.40 | |  |
|  | 32 | | 87.48 | | 84.89 | | 61.83 | | | 131 | | 82.43 | | 59.32 | | 51.84 | |  |
|  | 33 | | 87.49 | | 83.67 | | 61.84 | | | 132 | | 82.15 | | 59.32 | | 52.54 | |  |
|  | 34 | | 87.48 | | 83.67 | | 61.94 | | | 133 | | 82.08 | | 59.34 | | 53.11 | |  |
|  | 35 | | 87.47 | | 82.78 | | 61.94 | | | 134 | | 81.74 | | 59.22 | | 53.13 | |  |
|  | 36 | | 87.49 | | 81.7 | | 61.82 | | | 135 | | 81.42 | | 59.24 | | 53.13 | |  |
|  | 37 | | 87.52 | | 80.87 | | 61.84 | | | 136 | | 81.33 | | 59.18 | | 53.29 | |  |
|  | 38 | | 87.53 | | 79.79 | | 61.45 | | | 137 | | 81.09 | | 59.39 | | 53.41 | |  |
|  | 39 | | 87.53 | | 78.54 | | 62.00 | | | 138 | | 81.04 | | 59.53 | | 53.61 | |  |
|  | 40 | | 87.54 | | 77.44 | | 61.98 | | | 139 | | 80.78 | | 59.62 | | 53.82 | |  |
|  | 41 | | 87.55 | | 76.56 | | 61.99 | | | 140 | | 80.77 | | 59.72 | | 54.12 | |  |
|  | 42 | | 87.55 | | 76.28 | | 61.99 | | | 141 | | 80.73 | | 59.72 | | 54.25 | |  |
|  | 43 | | 87.49 | | 76.28 | | 61.97 | | | 142 | | 80.63 | | 59.98 | | 54.52 | |  |
|  | 44 | | 87.15 | | 76.08 | | 61.96 | | | 143 | | 80.55 | | 59.98 | | 54.41 | |  |
|  | 45 | | 86.72 | | 75.73 | | 61.59 | | | 144 | | 80.47 | | 60.10 | | 54.41 | |  |
|  | 46 | | 86.15 | | 75.48 | | 62.04 | | | 145 | | 80.22 | | 59.97 | | 54.26 | |  |
|  | 47 | | 85.75 | | 75.13 | | 61.71 | | | 146 | | 79.84 | | 60.11 | | 54.28 | |  |
|  | 48 | | 85.35 | | 74.60 | | 60.95 | | | 147 | | 79.80 | | 60.01 | | 53.75 | |  |
|  | 49 | | 85.28 | | 74.43 | | 60.74 | | | 148 | | 79.52 | | 59.94 | | 53.55 | |  |
|  | 50 | | 85.20 | | 73.78 | | 60.74 | | | 149 | | 79.42 | | 59.81 | | 52.95 | |  |
|  | 51 | | 85.15 | | 73.29 | | 60.75 | | | 150 | | 79.11 | | 59.81 | | 52.22 | |  |
|  | 52 | | 85.15 | | 73.29 | | 60.72 | | | 151 | | 78.72 | | 59.34 | | 51.55 | |  |
|  | 53 | | 85.17 | | 72.58 | | 59.99 | | | 152 | | 78.36 | | 59.06 | | 51.55 | |  |
|  | 54 | | 85.17 | | 71.94 | | 58.81 | | | 153 | | 78.05 | | 58.55 | | 50.31 | |  |
|  | 55 | | 85.16 | | 71.26 | | 57.75 | | | 154 | | 77.99 | | 58.35 | | 49.54 | |  |
|  | 56 | | 85.15 | | 70.53 | | 56.95 | | | 155 | | 77.65 | | 57.84 | | 49.24 | |  |
|  | 57 | | 85.10 | | 70.15 | | 56.09 | | | 156 | | 77.31 | | 57.74 | | 49.4 | |  |
|  | 58 | | 85.03 | | 69.62 | | 56.09 | | | 157 | | 77.22 | | 57.76 | | 49.77 | |  |
|  | 59 | | 84.95 | | 69.28 | | 55.37 | | | 158 | | 76.91 | | 57.67 | | 50.19 | |  |
|  | 60 | | 84.87 | | 68.88 | | 53.53 | | | 159 | | 76.82 | | 57.67 | | 49.95 | |  |
|  | 61 | | 84.62 | | 68.88 | | 53.22 | | | 160 | | 76.62 | | 57.76 | | 50.36 | |  |
|  | 62 | | 84.62 | | 68.39 | | 52.41 | | | 161 | | 76.62 | | 57.86 | | 50.36 | |  |
|  | 63 | | 84.59 | | 68.10 | | 52.58 | | | 162 | | 76.62 | | 57.71 | | 50.88 | |  |
|  | 64 | | 84.54 | | 67.68 | | 53.36 | | | 163 | | 76.63 | | 57.98 | | 51.41 | |  |
|  | 65 | | 84.47 | | 67.27 | | 53.69 | | | 164 | | 76.65 | | 58.05 | | 51.41 | |  |
|  | 66 | | 84.46 | | 66.89 | | 53.69 | | | 165 | | 76.66 | | 58.14 | | 51.63 | |  |
|  | 67 | | 84.44 | | 66.49 | | 54.08 | | | 166 | | 76.68 | | 58.19 | | 51.82 | |  |
|  | 68 | | 84.48 | | 66.23 | | 54.58 | | | 167 | | 76.66 | | 58.34 | | 52.61 | |  |
|  | 69 | | 84.53 | | 65.08 | | 55.26 | | | 168 | | 76.22 | | 58.34 | | 52.89 | |  |
|  | 70 | | 84.73 | | 65.08 | | 55.83 | | | 169 | | 75.66 | | 58.57 | | 52.89 | |  |
|  | 71 | | 84.76 | | 64.04 | | 56.48 | | | 170 | | 75.30 | | 58.43 | | 52.69 | |  |
|  | 72 | | 84.83 | | 62.96 | | 57.19 | | | 171 | | 74.75 | | 58.55 | | 52.70 | |  |
|  | 73 | | 85.06 | | 61.41 | | 57.53 | | | 172 | | 74.34 | | 58.47 | | 52.24 | |  |
|  | 74 | | 85.07 | | 60.44 | | 57.51 | | | 173 | | 73.98 | | 58.14 | | 52.22 | |  |
|  | 75 | | 85.15 | | 59.69 | | 57.51 | | | 174 | | 73.97 | | 58.05 | | 52.24 | |  |
|  | 76 | | 85.21 | | 59.32 | | 57.5 | | | 175 | | 73.91 | | 57.82 | | 52.14 | |  |
|  | 77 | | 85.29 | | 59.02 | | 57.49 | | | 176 | | 73.85 | | 57.79 | | 52.32 | |  |
|  | 78 | | 85.37 | | 58.97 | | 57.3 | | | 177 | | 73.8 | | 57.79 | | 52.55 | |  |
|  | 79 | | 85.51 | | 58.97 | | 57.31 | | | 178 | | 73.78 | | 57.63 | | 52.55 | |  |
|  | 80 | | 85.54 | | 58.96 | | 56.65 | | | 179 | | 73.82 | | 57.72 | | 52.68 | |  |
|  | 81 | | 85.56 | | 58.93 | | 55.92 | | | 180 | | 73.88 | | 57.93 | | 53.36 | |  |
|  | 82 | | 85.6 | | 58.79 | | 55.33 | | | 181 | | 73.97 | | 58.11 | | 53.76 | |  |
|  | 83 | | 85.64 | | 58.91 | | 55.2 | | | 182 | | 74.08 | | 58.31 | | 54.18 | |  |
|  | 84 | | 85.64 | | 58.67 | | 55.20 | | | 183 | | 74.16 | | 58.61 | | 53.90 | |  |
|  | 85 | | 85.65 | | 58.38 | | 55.38 | | | 184 | | 74.22 | | 59.16 | | 53.88 | |  |
|  | 86 | | 85.66 | | 58.34 | | 55.35 | | | 185 | | 74.28 | | 59.61 | | 53.89 | |  |
|  | 87 | | 85.68 | | 58.23 | | 55.39 | | | 186 | | 74.34 | | 59.61 | | 53.89 | |  |
|  | 88 | | 85.68 | | 58.23 | | 55.92 | | | 187 | | 74.42 | | 59.89 | | 54.07 | |  |
|  | 89 | | 85.67 | | 58.3 | | 55.90 | | | 188 | | 74.61 | | 60.16 | | 54.06 | |  |
|  | 90 | | 85.62 | | 58.32 | | 54.95 | | | 189 | | 74.67 | | 60.28 | | 54.55 | |  |
|  | 91 | | 85.31 | | 58.65 | | 54.19 | | | 190 | | 74.92 | | 60.25 | | 54.41 | |  |
|  | 92 | | 85.29 | | 59.16 | | 54.19 | | | 191 | | 74.98 | | 60.52 | | 54.54 | |  |
|  | 93 | | 84.96 | | 59.56 | | 52.55 | | | 192 | | 75.28 | | 60.64 | | 54.53 | |  |
|  | 94 | | 84.62 | | 60.09 | | 51.58 | | | 193 | | 75.35 | | 60.73 | | 53.7 | |  |
|  | 95 | | 84.20 | | 60.40 | | 51.17 | | | 194 | | 75.73 | | 60.72 | | 53.7 | |  |
|  | 96 | | 83.93 | | 60.85 | | 51.50 | | | 195 | | 75.78 | | 60.72 | | 53.16 | |  |
|  | 97 | | 83.94 | | 60.85 | | 52.15 | | 196 | | | 76.02 | | 60.77 | | 52.52 | |  |
|  | 98 | | 83.99 | | 60.87 | | 52.46 | | 197 | | | 76.13 | | 60.67 | | 51.75 | |  |
|  | 99 | | 84.30 | | 61.15 | | 53.44 | | | 198 | | 76.19 | | 60.70 | | 50.74 | |  |
| b | 1 | | | 83.8 | | 81.58 | | 57.21 | | 50 | | | 83.05 | | 41.93 | | 59.11 | |
|  | 2 | | | 83.77 | | 81.61 | | 57.09 | | 51 | | | 83.07 | | 40.47 | | 58.23 | |
|  | 3 | | | 83.76 | | 81.75 | | 57.71 | | 52 | | | 83.06 | | 38.77 | | 56.97 | |
|  | 4 | | | 83.69 | | 81.75 | | 57.71 | | 53 | | | 83.04 | | 37.38 | | 56.97 | |
|  | 5 | | | 83.64 | | 81.60 | | 57.72 | | 54 | | | 83.00 | | 35.79 | | 55.85 | |
|  | 6 | | | 83.46 | | 81.71 | | 57.73 | | 55 | | | 82.99 | | 34.44 | | 56.25 | |
|  | 7 | | | 83.49 | | 81.78 | | 57.72 | | 56 | | | 82.94 | | 32.95 | | 55.01 | |
|  | 8 | | | 83.51 | | 81.67 | | 57.90 | | 57 | | | 82.70 | | 31.89 | | 53.65 | |
|  | 9 | | | 83.53 | | 81.84 | | 58.11 | | 58 | | | 82.68 | | 31.89 | | 53.65 | |
|  | 10 | | | 83.48 | | 81.72 | | 58.10 | | 59 | | | 82.62 | | 30.79 | | 52.86 | |
|  | 11 | | | 83.48 | | 81.82 | | 58.01 | | 60 | | | 82.34 | | 30.14 | | 54.02 | |
|  | 12 | | | 83.47 | | 81.61 | | 58.01 | | 61 | | | 82.31 | | 29.60 | | 52.21 | |
|  | 13 | | | 83.45 | | 81.61 | | 57.99 | | 62 | | | 82.06 | | 29.47 | | 50.83 | |
|  | 14 | | | 83.42 | | 81.67 | | 57.33 | | 63 | | | 82.06 | | 29.15 | | 51.10 | |
|  | 15 | | | 83.42 | | 81.62 | | 57.34 | | 64 | | | 82.03 | | 29.27 | | 50.83 | |
|  | 16 | | | 83.40 | | 81.61 | | 57.69 | | 65 | | | 81.97 | | 29.52 | | 49.74 | |
|  | 17 | | | 83.40 | | 81.46 | | 57.69 | | 66 | | | 81.91 | | 29.91 | | 49.74 | |
|  | 18 | | | 83.39 | | 81.41 | | 57.26 | | 67 | | | 81.87 | | 29.91 | | 48.71 | |
|  | 19 | | | 83.36 | | 81.16 | | 57.41 | | 68 | | | 81.84 | | 29.95 | | 47.55 | |
|  | 20 | | | 83.35 | | 80.94 | | 57.41 | | 69 | | | 81.81 | | 29.76 | | 46.91 | |
|  | 21 | | | 83.34 | | 80.74 | | 57.13 | | 70 | | | 81.79 | | 29.79 | | 46.39 | |
|  | 22 | | | 83.36 | | 80.74 | | 57.48 | | 71 | | | 81.78 | | 29.68 | | 46.98 | |
|  | 23 | | | 83.35 | | 80.66 | | 57.02 | | 72 | | | 81.76 | | 29.40 | | 46.48 | |
|  | 24 | | | 83.35 | | 80.41 | | 56.72 | | 73 | | | 81.73 | | 29.52 | | 48.31 | |
|  | 25 | | | 83.34 | | 80.24 | | 56.72 | | 74 | | | 81.74 | | 29.54 | | 46.40 | |
|  | 26 | | | 83.34 | | 80.08 | | 56.86 | | 75 | | | 81.72 | | 29.62 | | 46.40 | |
|  | 27 | | | 83.33 | | 79.92 | | 56.86 | | 76 | | | 81.72 | | 29.62 | | 48.49 | |
|  | 28 | | | 83.31 | | 79.71 | | 56.74 | | 77 | | | 81.73 | | 29.85 | | 48.23 | |
|  | 29 | | | 83.30 | | 79.35 | | 56.74 | | 78 | | | 81.77 | | 30.16 | | 48.77 | |
|  | 30 | | | 83.28 | | 79.20 | | 57.11 | | 79 | | | 81.76 | | 30.51 | | 47.64 | |
|  | 31 | | | 83.29 | | 79.20 | | 57.50 | | 80 | | | 81.79 | | 30.89 | | 48.99 | |
|  | 32 | | | 83.27 | | 78.32 | | 57.81 | | 81 | | | 81.79 | | 31.15 | | 47.95 | |
|  | 33 | | | 83.26 | | 76.62 | | 57.44 | | 82 | | | 81.85 | | 31.73 | | 48.85 | |
|  | 34 | | | 83.26 | | 73.32 | | 58.16 | | 83 | | | 81.88 | | 32.24 | | 48.85 | |
|  | 35 | | | 83.24 | | 71.94 | | 58.16 | | 84 | | | 81.92 | | 32.84 | | 50.15 | |
|  | 36 | | | 83.24 | | 68.96 | | 59.32 | | 85 | | | 81.97 | | 32.84 | | 48.64 | |
|  | 37 | | | 83.22 | | 65.42 | | 59.50 | | 86 | | | 82.18 | | 33.34 | | 48.76 | |
|  | 38 | | | 83.21 | | 62.05 | | 59.81 | | 87 | | | 82.20 | | 33.81 | | 48.94 | |
|  | 39 | | | 83.19 | | 58.35 | | 60.03 | | 88 | | | 82.48 | | 34.48 | | 48.6 | |
|  | 40 | | | 83.18 | | 58.35 | | 60.01 | | 89 | | | 82.53 | | 35.06 | | 49.30 | |
|  | 41 | | | 83.18 | | 55.17 | | 59.92 | | 90 | | | 82.60 | | 35.46 | | 48.95 | |
|  | 42 | | | 83.18 | | 53.01 | | 59.56 | | 91 | | | 82.82 | | 35.97 | | 49.66 | |
|  | 43 | | | 83.15 | | 51.00 | | 59.56 | | 92 | | | 82.80 | | 36.52 | | 49.66 | |
|  | 44 | | | 83.15 | | 48.96 | | 59.90 | | 93 | | | 82.77 | | 36.99 | | 49.72 | |
|  | 45 | | | 83.13 | | 47.18 | | 59.47 | | 94 | | | 82.77 | | 36.99 | | 49.22 | |
|  | 46 | | | 83.11 | | 45.91 | | 59.63 | | 95 | | | 82.84 | | 37.65 | | 49.95 | |
|  | 47 | | | 83.11 | | 44.64 | | 59.38 | | 96 | | | 82.93 | | 38.26 | | 50.10 | |
|  | 48 | | | 83.08 | | 43.35 | | 59.39 | | 97 | | | 83.15 | | 38.79 | | 50.40 | |
|  | 49 | | | 83.06 | | 43.35 | | 59.11 | | 98 | | | 83.47 | | 39.35 | | 50.70 | |
| c | 1 | | | 87.37 | | 85.47 | | 60.34 | | 50 | | | 84.83 | | 38.15 | | 63.31 | |
|  | 2 | | | 87.33 | | 85.47 | | 60.53 | | 51 | | | 84.79 | | 37.05 | | 62.89 | |
|  | 3 | | | 87.25 | | 85.47 | | 60.52 | | 52 | | | 84.77 | | 36.32 | | 62.60 | |
|  | 4 | | | 87.18 | | 85.59 | | 60.63 | | 53 | | | 84.76 | | 35.78 | | 62.60 | |
|  | 5 | | | 86.95 | | 85.52 | | 61.45 | | 54 | | | 84.76 | | 35.12 | | 62.60 | |
|  | 6 | | | 86.60 | | 85.58 | | 61.05 | | 55 | | | 84.79 | | 35.12 | | 62.49 | |
|  | 7 | | | 86.56 | | 85.53 | | 60.63 | | 56 | | | 84.78 | | 34.94 | | 62.02 | |
|  | 8 | | | 86.26 | | 85.59 | | 60.63 | | 57 | | | 84.75 | | 34.76 | | 60.23 | |
|  | 9 | | | 86.22 | | 85.61 | | 60.35 | | 58 | | | 84.74 | | 34.5 | | 59.66 | |
|  | 10 | | | 86.18 | | 85.55 | | 60.34 | | 59 | | | 84.74 | | 34.2 | | 59.13 | |
|  | 11 | | | 85.93 | | 85.55 | | 60.16 | | 60 | | | 84.75 | | 33.84 | | 57.47 | |
|  | 12 | | | 85.96 | | 85.76 | | 60.19 | | 61 | | | 84.74 | | 33.58 | | 57.47 | |
|  | 13 | | | 85.97 | | 85.76 | | 60.20 | | 62 | | | 84.74 | | 33.37 | | 55.56 | |
|  | 14 | | | 85.97 | | 85.77 | | 60.70 | | 63 | | | 84.73 | | 33.12 | | 54.33 | |
|  | 15 | | | 85.97 | | 85.74 | | 61.09 | | 64 | | | 84.73 | | 33.12 | | 53.14 | |
|  | 16 | | | 85.96 | | 85.75 | | 61.09 | | 65 | | | 84.7 | | 33.07 | | 50.75 | |
|  | 17 | | | 85.94 | | 85.76 | | 61.09 | | 66 | | | 84.75 | | 33.2 | | 50.76 | |
|  | 18 | | | 85.92 | | 85.99 | | 60.74 | | 67 | | | 84.74 | | 33.46 | | 50.06 | |
|  | 19 | | | 85.92 | | 86.02 | | 60.72 | | 68 | | | 84.75 | | 33.73 | | 48.79 | |
|  | 20 | | | 85.89 | | 86.02 | | 60.85 | | 69 | | | 84.76 | | 34.18 | | 50.05 | |
|  | 21 | | | 85.89 | | 85.88 | | 60.56 | | 70 | | | 84.74 | | 34.68 | | 50.05 | |
|  | 22 | | | 85.90 | | 86.02 | | 60.88 | | 71 | | | 84.76 | | 35.39 | | 48.89 | |
|  | 23 | | | 85.87 | | 86.01 | | 60.88 | | 72 | | | 84.76 | | 35.95 | | 50.63 | |
|  | 24 | | | 85.85 | | 86.00 | | 60.71 | | 73 | | | 84.74 | | 35.95 | | 49.00 | |
|  | 25 | | | 85.80 | | 85.89 | | 60.55 | | 74 | | | 84.73 | | 36.64 | | 49.70 | |
|  | 26 | | | 85.76 | | 85.76 | | 60.72 | | 75 | | | 84.74 | | 37.5 | | 50.64 | |
|  | 27 | | | 85.71 | | 85.72 | | 61.35 | | 76 | | | 84.76 | | 38.27 | | 49.82 | |
|  | 28 | | | 85.65 | | 85.73 | | 61.35 | | 77 | | | 84.74 | | 39.17 | | 50.20 | |
|  | 29 | | | 85.59 | | 85.73 | | 61.42 | | 78 | | | 84.75 | | 39.94 | | 49.98 | |
|  | 30 | | | 85.54 | | 85.56 | | 61.54 | | 79 | | | 84.75 | | 40.79 | | 49.98 | |
|  | 31 | | | 85.47 | | 85.42 | | 61.54 | | 80 | | | 84.75 | | 41.61 | | 49.67 | |
|  | 32 | | | 85.42 | | 85.27 | | 61.54 | | 81 | | | 84.77 | | 42.36 | | 50.44 | |
|  | 33 | | | 85.37 | | 84.80 | | 61.42 | | 82 | | | 84.79 | | 42.36 | | 50.86 | |
|  | 34 | | | 85.31 | | 80.21 | | 61.03 | | 83 | | | 84.78 | | 42.93 | | 49.90 | |
|  | 35 | | | 85.24 | | 73.17 | | 61.28 | | 84 | | | 84.75 | | 43.53 | | 49.99 | |
|  | 36 | | | 85.16 | | 63.73 | | 61.64 | | 85 | | | 84.76 | | 44.2 | | 50.29 | |
|  | 37 | | | 85.10 | | 58.10 | | 61.18 | | 86 | | | 84.74 | | 44.92 | | 50.31 | |
|  | 38 | | | 85.05 | | 58.10 | | 61.18 | | 87 | | | 84.74 | | 45.44 | | 50.31 | |
|  | 39 | | | 85.02 | | 53.03 | | 60.83 | | 88 | | | 84.72 | | 46.19 | | 50.61 | |
|  | 40 | | | 85.00 | | 49.56 | | 60.84 | | 89 | | | 84.71 | | 46.81 | | 50.74 | |
|  | 41 | | | 84.97 | | 47.25 | | 60.98 | | 90 | | | 84.69 | | 47.24 | | 50.73 | |
|  | 42 | | | 84.94 | | 45.12 | | 60.99 | | 91 | | | 84.70 | | 47.24 | | 50.67 | |
|  | 43 | | | 84.92 | | 43.80 | | 60.88 | | 92 | | | 84.70 | | 47.76 | | 51.07 | |
|  | 44 | | | 84.93 | | 42.34 | | 61.40 | | 93 | | | 84.68 | | 48.07 | | 52.03 | |
|  | 45 | | | 84.89 | | 41.38 | | 62.42 | | 94 | | | 84.66 | | 48.48 | | 51.37 | |
|  | 46 | | | 84.89 | | 40.89 | | 62.42 | | 95 | | | 84.63 | | 48.9 | | 51.25 | |
|  | 47 | | | 84.86 | | 40.89 | | 63.31 | | 96 | | | 84.61 | | 49.4 | | 51.25 | |
|  | 48 | | | 84.89 | | 40.07 | | 63.79 | | 97 | | | 84.53 | | 50.04 | | 51.87 | |
|  | 49 | | | 84.84 | | 39.20 | | 63.54 | | 98 | | | 84.49 | | 50.44 | | 52.28 | |
| d | 1 | | | 95.09 | | 87.23 | | 55.38 | | 100 | | | 84.33 | | 57.91 | | 51.79 | |
|  | 2 | | | 94.2 | | 87.37 | | 55.38 | | 101 | | | 83.99 | | 58.37 | | 51.79 | |
|  | 3 | | | 93.53 | | 87.5 | | 56.02 | | 102 | | | 83.95 | | 58.73 | | 51.66 | |
|  | 4 | | | 92.98 | | 87.55 | | 56.14 | | 103 | | | 83.64 | | 58.73 | | 51.35 | |
|  | 5 | | | 92.6 | | 87.54 | | 56.47 | | 104 | | | 83.55 | | 59.22 | | 50.79 | |
|  | 6 | | | 92.56 | | 87.54 | | 56.32 | | 105 | | | 83.21 | | 59.6 | | 50.28 | |
|  | 7 | | | 92.52 | | 87.6 | | 56.13 | | 106 | | | 83.16 | | 59.89 | | 49.64 | |
|  | 8 | | | 92.48 | | 87.87 | | 56.14 | | 107 | | | 83.08 | | 60.22 | | 49.15 | |
|  | 9 | | | 92.44 | | 87.85 | | 56.14 | | 108 | | | 83.01 | | 60.44 | | 48.95 | |
|  | 10 | | | 92.18 | | 87.95 | | 56.1 | | 109 | | | 82.95 | | 60.82 | | 48.93 | |
|  | 11 | | | 92.18 | | 87.92 | | 56.09 | | 110 | | | 82.93 | | 60.67 | | 48.93 | |
|  | 12 | | | 92.15 | | 88.08 | | 56.55 | | 111 | | | 82.87 | | 60.63 | | 48.52 | |
|  | 13 | | | 92.11 | | 87.99 | | 56.31 | | 112 | | | 82.82 | | 60.63 | | 48.82 | |
|  | 14 | | | 91.85 | | 87.8 | | 56.34 | | 113 | | | 82.77 | | 60.91 | | 48.86 | |
|  | 15 | | | 91.8 | | 87.8 | | 56.16 | | 114 | | | 82.47 | | 61.17 | | 49.11 | |
|  | 16 | | | 91.52 | | 86.85 | | 55.73 | | 115 | | | 82.45 | | 61.18 | | 49.32 | |
|  | 17 | | | 91.14 | | 83.93 | | 55.73 | | 116 | | | 82.38 | | 61.3 | | 49.28 | |
|  | 18 | | | 90.8 | | 79.16 | | 55.6 | | 117 | | | 82.14 | | 61.6 | | 50.03 | |
|  | 19 | | | 90.71 | | 73.85 | | 55.26 | | 118 | | | 82.11 | | 62.02 | | 50.03 | |
|  | 20 | | | 90.28 | | 69.77 | | 55.45 | | 119 | | | 82.02 | | 62.33 | | 50.31 | |
|  | 21 | | | 90.23 | | 64.43 | | 55.79 | | 120 | | | 81.83 | | 62.62 | | 50.8 | |
|  | 22 | | | 90.43 | | 61.91 | | 56.54 | | 121 | | | 81.77 | | 62.62 | | 50.98 | |
|  | 23 | | | 90.74 | | 62.34 | | 55.91 | | 122 | | | 81.5 | | 63.1 | | 51.45 | |
|  | 24 | | | 90.77 | | 62.34 | | 56.52 | | 123 | | | 81.43 | | 63.67 | | 51.88 | |
|  | 25 | | | 90.79 | | 63.58 | | 56.52 | | 124 | | | 81.24 | | 63.94 | | 52.17 | |
|  | 26 | | | 90.85 | | 65.14 | | 56.92 | | 125 | | | 81.16 | | 64.05 | | 52.19 | |
|  | 27 | | | 90.87 | | 66.22 | | 56.93 | | 126 | | | 80.77 | | 64.02 | | 52.19 | |
|  | 28 | | | 91.21 | | 67.08 | | 56.27 | | 127 | | | 80.45 | | 64.13 | | 51.76 | |
|  | 29 | | | 91.52 | | 67.56 | | 55.75 | | 128 | | | 80.38 | | 64.42 | | 52.01 | |
|  | 30 | | | 91.6 | | 68.15 | | 55.77 | | 129 | | | 79.94 | | 64.89 | | 51.8 | |
|  | 31 | | | 91.85 | | 68.46 | | 54.98 | | 130 | | | 79.6 | | 64.89 | | 52.17 | |
|  | 32 | | | 91.85 | | 68.62 | | 53.69 | | 131 | | | 79.25 | | 65.2 | | 51.74 | |
|  | 33 | | | 91.82 | | 68.62 | | 53.69 | | 132 | | | 78.87 | | 65.49 | | 51.22 | |
|  | 34 | | | 91.77 | | 68.35 | | 52.83 | | 133 | | | 78.56 | | 65.67 | | 51.93 | |
|  | 35 | | | 91.58 | | 67.89 | | 52.62 | | 134 | | | 78.5 | | 66 | | 51.74 | |
|  | 36 | | | 91.54 | | 66.78 | | 51.73 | | 135 | | | 78.42 | | 66.25 | | 51.74 | |
|  | 37 | | | 91.52 | | 65.73 | | 51.57 | | 136 | | | 78.24 | | 66.5 | | 51.73 | |
|  | 38 | | | 91.51 | | 64.79 | | 51.28 | | 137 | | | 77.92 | | 66.82 | | 51.41 | |
|  | 39 | | | 91.52 | | 63.91 | | 50.47 | | 138 | | | 77.89 | | 66.82 | | 51.13 | |
|  | 40 | | | 91.52 | | 63.25 | | 50.49 | | 139 | | | 77.84 | | 67.07 | | 50.51 | |
|  | 41 | | | 91.49 | | 63.25 | | 50.49 | | 140 | | | 77.78 | | 67.08 | | 50.2 | |
|  | 42 | | | 91.51 | | 62.75 | | 50.35 | | 141 | | | 77.74 | | 67.04 | | 49.99 | |
|  | 43 | | | 91.54 | | 62.46 | | 49.91 | | 142 | | | 77.7 | | 67.06 | | 49.38 | |
|  | 44 | | | 91.61 | | 61.99 | | 49.91 | | 143 | | | 77.66 | | 67.24 | | 49.17 | |
|  | 45 | | | 91.68 | | 61.8 | | 49.31 | | 144 | | | 77.45 | | 67.5 | | 49.17 | |
|  | 46 | | | 91.72 | | 61.59 | | 49.87 | | 145 | | | 77.38 | | 67.34 | | 49.19 | |
|  | 47 | | | 91.69 | | 61.18 | | 49.87 | | 146 | | | 76.92 | | 67.53 | | 49.03 | |
|  | 48 | | | 91.64 | | 60.9 | | 49.21 | | 147 | | | 76.47 | | 67.53 | | 49.03 | |
|  | 49 | | | 91.58 | | 60.61 | | 49.21 | | 148 | | | 76.01 | | 67.36 | | 49.15 | |
|  | 50 | | | 91.5 | | 60.61 | | 48.78 | | 149 | | | 75.67 | | 67.41 | | 49.33 | |
|  | 51 | | | 91.2 | | 60.51 | | 48.39 | | 150 | | | 75.27 | | 67.33 | | 49.14 | |
|  | 52 | | | 91.15 | | 60.4 | | 48.57 | | 151 | | | 74.93 | | 67.5 | | 49.15 | |
|  | 53 | | | 90.87 | | 60.62 | | 48.26 | | 152 | | | 74.58 | | 67.59 | | 49.27 | |
|  | 54 | | | 90.78 | | 60.77 | | 48.4 | | 153 | | | 74.25 | | 67.61 | | 49.27 | |
|  | 55 | | | 90.7 | | 61.02 | | 47.25 | | 154 | | | 74.2 | | 67.13 | | 49.93 | |
|  | 56 | | | 90.63 | | 61.21 | | 47.66 | | 155 | | | 73.97 | | 66.83 | | 50.42 | |
|  | 57 | | | 90.65 | | 61.57 | | 47.01 | | 156 | | | 73.97 | | 66.83 | | 50.58 | |
|  | 58 | | | 90.7 | | 61.57 | | 47.01 | | 157 | | | 73.96 | | 66.46 | | 50.25 | |
|  | 59 | | | 90.71 | | 61.57 | | 47.18 | | 158 | | | 73.84 | | 66.14 | | 50.23 | |
|  | 60 | | | 90.43 | | 61.73 | | 47.92 | | 159 | | | 73.63 | | 65.71 | | 50.25 | |
|  | 61 | | | 90.38 | | 62.04 | | 47.53 | | 160 | | | 73.6 | | 65.36 | | 49.77 | |
|  | 62 | | | 90.13 | | 62.49 | | 47.54 | | 161 | | | 73.53 | | 65.16 | | 49.77 | |
|  | 63 | | | 90.1 | | 62.7 | | 48.8 | | 162 | | | 73.47 | | 64.92 | | 49.53 | |
|  | 64 | | | 90.07 | | 62.99 | | 47.14 | | 163 | | | 73.4 | | 64.67 | | 49.76 | |
|  | 65 | | | 90.06 | | 62.82 | | 47.14 | | 164 | | | 73.36 | | 64.39 | | 50.44 | |
|  | 66 | | | 90.01 | | 62.96 | | 47.14 | | 165 | | | 73.33 | | 64.39 | | 50.44 | |
|  | 67 | | | 89.95 | | 62.66 | | 46.92 | | 166 | | | 73.3 | | 64.38 | | 50.17 | |
|  | 68 | | | 89.86 | | 62.66 | | 46.91 | | 167 | | | 73.28 | | 64.56 | | 50.17 | |
|  | 69 | | | 89.78 | | 62.17 | | 46.94 | | 168 | | | 73.27 | | 64.83 | | 50.33 | |
|  | 70 | | | 89.7 | | 61.95 | | 46.52 | | 169 | | | 73.26 | | 65.13 | | 50.1 | |
|  | 71 | | | 89.66 | | 61.36 | | 46.51 | | 170 | | | 73.27 | | 65.26 | | 50.1 | |
|  | 72 | | | 89.6 | | 60.99 | | 47.19 | | 171 | | | 73.27 | | 65.4 | | 50.23 | |
|  | 73 | | | 89.57 | | 60.35 | | 47.15 | | 172 | | | 73.3 | | 65.2 | | 50 | |
|  | 74 | | | 89.56 | | 60 | | 47.65 | | 173 | | | 73.32 | | 64.71 | | 49.98 | |
|  | 75 | | | 89.56 | | 59.38 | | 47.65 | | 174 | | | 73.35 | | 64.71 | | 49.77 | |
|  | 76 | | | 89.55 | | 58.58 | | 47.66 | | 175 | | | 73.4 | | 64.7 | | 50 | |
|  | 77 | | | 89.58 | | 58.58 | | 47.67 | | 176 | | | 73.49 | | 64.8 | | 50.22 | |
|  | 78 | | | 89.58 | | 57.4 | | 47.99 | | 177 | | | 73.7 | | 64.89 | | 50.22 | |
|  | 79 | | | 89.58 | | 56.45 | | 48.31 | | 178 | | | 73.74 | | 65.2 | | 50.01 | |
|  | 80 | | | 89.56 | | 55.69 | | 49.04 | | 179 | | | 74.03 | | 65.51 | | 49.66 | |
|  | 81 | | | 89.49 | | 55.22 | | 49.26 | | 180 | | | 74.03 | | 65.89 | | 49.14 | |
|  | 82 | | | 89.21 | | 54.83 | | 49.51 | | 181 | | | 74.08 | | 66.25 | | 48.91 | |
|  | 83 | | | 89.19 | | 54.67 | | 49.34 | | 182 | | | 74.33 | | 66.35 | | 48.91 | |
|  | 84 | | | 88.84 | | 54.63 | | 49.34 | | 183 | | | 74.39 | | 66.35 | | 49.24 | |
|  | 85 | | | 88.31 | | 54.63 | | 50.04 | | 184 | | | 74.67 | | 66.63 | | 49.57 | |
|  | 86 | | | 87.88 | | 54.72 | | 49.59 | | 185 | | | 74.71 | | 66.89 | | 49.57 | |
|  | 87 | | | 87.48 | | 54.72 | | 49.29 | | 186 | | | 74.72 | | 66.75 | | 49.72 | |
|  | 88 | | | 87.07 | | 54.73 | | 48.99 | | 187 | | | 74.75 | | 66.88 | | 49.54 | |
|  | 89 | | | 87.01 | | 54.68 | | 48.43 | | 188 | | | 74.74 | | 66.65 | | 49.43 | |
|  | 90 | | | 86.57 | | 54.71 | | 48.67 | | 189 | | | 74.75 | | 66.55 | | 48.91 | |
|  | 91 | | | 85.8 | | 54.92 | | 48.15 | | 190 | | | 75 | | 66.12 | | 48.67 | |
|  | 92 | | | 85.4 | | 54.97 | | 48.15 | | 191 | | | 75.01 | | 66.12 | | 48.12 | |
|  | 93 | | | 85.07 | | 55.26 | | 48.88 | | 192 | | | 75.37 | | 65.62 | | 48.09 | |
|  | 94 | | | 85.04 | | 55.26 | | 49.36 | | 193 | | | 75.41 | | 65.05 | | 48.09 | |
|  | 95 | | | 84.97 | | 55.49 | | 50.18 | | 194 | | | 75.47 | | 64.17 | | 47.74 | |
|  | 96 | | | 84.77 | | 55.69 | | 50.72 | | 195 | | | 75.55 | | 63.45 | | 47.88 | |
|  | 97 | | | 84.73 | | 56.37 | | 51.44 | | 196 | | | 75.63 | | 62.79 | | 48.32 | |
|  | 98 | | | 84.67 | | 56.77 | | 51.74 | | 197 | | | 75.72 | | 61.83 | | 48.35 | |
|  | 99 | | | 84.36 | | 57.48 | | 52.09 | | 198 | | | 76.01 | | 61.28 | | 48.31 | |
| e | 1 | | | 85.4 | | 82.56 | | 57.91 | | 50 | | | 83.2 | | 29.8 | | 46.33 | |
|  | 2 | | | 85.38 | | 82.58 | | 58.2 | | 51 | | | 83.19 | | 29.54 | | 44.68 | |
|  | 3 | | | 85.35 | | 82.34 | | 58.2 | | 52 | | | 83.17 | | 29.51 | | 45.79 | |
|  | 4 | | | 85.31 | | 82.34 | | 58.01 | | 53 | | | 83.15 | | 29.29 | | 45.79 | |
|  | 5 | | | 85.22 | | 82.32 | | 53.18 | | 54 | | | 83.11 | | 29.38 | | 46 | |
|  | 6 | | | 85 | | 82.34 | | 55.33 | | 55 | | | 83.08 | | 29.63 | | 45.98 | |
|  | 7 | | | 84.96 | | 82.35 | | 56.16 | | 56 | | | 83.07 | | 29.65 | | 46.02 | |
|  | 8 | | | 84.9 | | 82.25 | | 56.9 | | 57 | | | 83.07 | | 29.65 | | 46.45 | |
|  | 9 | | | 84.82 | | 82.24 | | 56.37 | | 58 | | | 83.08 | | 29.84 | | 46.19 | |
|  | 10 | | | 84.73 | | 82.15 | | 56.36 | | 59 | | | 83.09 | | 29.8 | | 46.19 | |
|  | 11 | | | 84.66 | | 82.2 | | 56.36 | | 60 | | | 83.1 | | 29.94 | | 46.35 | |
|  | 12 | | | 84.63 | | 82.2 | | 56.68 | | 61 | | | 83.09 | | 30.07 | | 47.07 | |
|  | 13 | | | 84.59 | | 82.08 | | 56.7 | | 62 | | | 83.08 | | 30.3 | | 46.93 | |
|  | 14 | | | 84.38 | | 82.24 | | 56.69 | | 63 | | | 83.04 | | 30.27 | | 47.7 | |
|  | 15 | | | 84.36 | | 82.19 | | 56.7 | | 64 | | | 83.01 | | 30.34 | | 47.38 | |
|  | 16 | | | 84.32 | | 82.23 | | 56.57 | | 65 | | | 82.99 | | 30.54 | | 47.38 | |
|  | 17 | | | 84.27 | | 82.23 | | 56.71 | | 66 | | | 82.96 | | 30.54 | | 48.64 | |
|  | 18 | | | 84.18 | | 82.27 | | 56.72 | | 67 | | | 82.95 | | 30.39 | | 48.5 | |
|  | 19 | | | 84.11 | | 81.95 | | 56.72 | | 68 | | | 82.91 | | 30.68 | | 48.5 | |
|  | 20 | | | 84.07 | | 80.66 | | 57.22 | | 69 | | | 82.89 | | 30.96 | | 48.28 | |
|  | 21 | | | 84.04 | | 80.66 | | 57.21 | | 70 | | | 82.87 | | 31.18 | | 48.86 | |
|  | 22 | | | 84.01 | | 79.23 | | 56.99 | | 71 | | | 82.84 | | 31.5 | | 49.4 | |
|  | 23 | | | 83.97 | | 74.8 | | 56.84 | | 72 | | | 82.79 | | 31.7 | | 48.77 | |
|  | 24 | | | 83.94 | | 70.48 | | 56.85 | | 73 | | | 82.78 | | 32.22 | | 49.54 | |
|  | 25 | | | 83.91 | | 65.24 | | 56.97 | | 74 | | | 82.76 | | 32.66 | | 49.79 | |
|  | 26 | | | 83.88 | | 60.91 | | 56.97 | | 75 | | | 82.72 | | 32.66 | | 49.93 | |
|  | 27 | | | 83.84 | | 57.48 | | 56.98 | | 76 | | | 82.66 | | 33.03 | | 49.93 | |
|  | 28 | | | 83.8 | | 54.72 | | 57.01 | | 77 | | | 82.62 | | 33.6 | | 49.82 | |
|  | 29 | | | 83.77 | | 52.83 | | 56.93 | | 78 | | | 82.57 | | 34.23 | | 50.01 | |
|  | 30 | | | 83.73 | | 52.83 | | 56.42 | | 79 | | | 82.55 | | 34.66 | | 49.61 | |
|  | 31 | | | 83.68 | | 51.17 | | 55.48 | | 80 | | | 82.53 | | 35.25 | | 50.01 | |
|  | 32 | | | 83.66 | | 49.9 | | 54.75 | | 81 | | | 82.52 | | 35.99 | | 50.02 | |
|  | 33 | | | 83.62 | | 48.54 | | 53.8 | | 82 | | | 82.51 | | 36.75 | | 50.14 | |
|  | 34 | | | 83.6 | | 47.2 | | 53.8 | | 83 | | | 82.5 | | 37.63 | | 50.17 | |
|  | 35 | | | 83.57 | | 45.53 | | 53.41 | | 84 | | | 82.49 | | 37.63 | | 50.34 | |
|  | 36 | | | 83.57 | | 43.93 | | 52.78 | | 85 | | | 82.48 | | 38.39 | | 50.34 | |
|  | 37 | | | 83.55 | | 41.99 | | 50.96 | | 86 | | | 82.46 | | 39.28 | | 50.33 | |
|  | 38 | | | 83.52 | | 40.42 | | 49.3 | | 87 | | | 82.4 | | 40.44 | | 50.57 | |
|  | 39 | | | 83.48 | | 40.42 | | 49.13 | | 88 | | | 82.09 | | 41.46 | | 50.54 | |
|  | 40 | | | 83.45 | | 38.81 | | 47.73 | | 89 | | | 81.68 | | 42.51 | | 50.55 | |
|  | 41 | | | 83.44 | | 37.42 | | 47.74 | | 90 | | | 81.27 | | 43.64 | | 50.74 | |
|  | 42 | | | 83.42 | | 35.98 | | 47.74 | | 91 | | | 80.89 | | 44.88 | | 50.75 | |
|  | 43 | | | 83.4 | | 34.83 | | 46.95 | | 92 | | | 80.49 | | 46.25 | | 50.74 | |
|  | 44 | | | 83.36 | | 33.66 | | 46.39 | | 93 | | | 80.1 | | 46.25 | | 50.74 | |
|  | 45 | | | 83.34 | | 32.53 | | 46.38 | | 94 | | | 80.06 | | 47.56 | | 50.71 | |
|  | 46 | | | 83.3 | | 31.67 | | 46.02 | | 95 | | | 79.98 | | 48.81 | | 50.69 | |
|  | 47 | | | 83.27 | | 30.92 | | 44.97 | | 96 | | | 79.73 | | 50.01 | | 50.86 | |
|  | 48 | | | 83.22 | | 30.92 | | 45.66 | | 97 | | | 79.42 | | 51.18 | | 50.64 | |
|  | 49 | | | 83.19 | | 30.29 | | 46.33 | | 98 | | | 79.38 | | 52.51 | | 50.45 | |
| f | 1 | | | 85.49 | | 86.61 | | 58.03 | | 50 | | | 84.51 | | 34.24 | | 47.63 | |
|  | 2 | | | 85.46 | | 86.62 | | 58.14 | | 51 | | | 84.49 | | 34.07 | | 46.81 | |
|  | 3 | | | 85.43 | | 86.4 | | 58.15 | | 52 | | | 84.51 | | 33.74 | | 48.6 | |
|  | 4 | | | 85.38 | | 86.44 | | 57.88 | | 53 | | | 84.5 | | 33.74 | | 49.56 | |
|  | 5 | | | 85.32 | | 86.24 | | 58.33 | | 54 | | | 84.48 | | 33.88 | | 49.73 | |
|  | 6 | | | 85.27 | | 86.17 | | 58.33 | | 55 | | | 84.48 | | 33.61 | | 49.73 | |
|  | 7 | | | 85.21 | | 86.16 | | 58.17 | | 56 | | | 84.48 | | 33.66 | | 49.25 | |
|  | 8 | | | 85.19 | | 86.16 | | 57.83 | | 57 | | | 84.48 | | 33.65 | | 47.63 | |
|  | 9 | | | 85.16 | | 85.91 | | 57.85 | | 58 | | | 84.47 | | 33.48 | | 49.8 | |
|  | 10 | | | 85.13 | | 85.9 | | 57.8 | | 59 | | | 84.48 | | 33.62 | | 48.59 | |
|  | 11 | | | 85.11 | | 85.76 | | 58.13 | | 60 | | | 84.51 | | 33.81 | | 48.98 | |
|  | 12 | | | 85.10 | | 85.61 | | 58.38 | | 61 | | | 84.51 | | 34.26 | | 49.95 | |
|  | 13 | | | 85.11 | | 85.32 | | 58.58 | | 62 | | | 84.51 | | 34.26 | | 49.36 | |
|  | 14 | | | 85.12 | | 85.26 | | 58.58 | | 63 | | | 84.51 | | 34.63 | | 49.36 | |
|  | 15 | | | 85.13 | | 85.16 | | 58.89 | | 64 | | | 84.52 | | 34.93 | | 49.55 | |
|  | 16 | | | 85.1 | | 85 | | 58.92 | | 65 | | | 84.52 | | 35.15 | | 49.86 | |
|  | 17 | | | 85.09 | | 85 | | 58.53 | | 66 | | | 84.53 | | 35.47 | | 50.44 | |
|  | 18 | | | 85.08 | | 84.75 | | 59.13 | | 67 | | | 84.52 | | 35.71 | | 50.22 | |
|  | 19 | | | 85.08 | | 84.76 | | 59.12 | | 68 | | | 84.53 | | 36 | | 50.22 | |
|  | 20 | | | 85.06 | | 82.89 | | 59.69 | | 69 | | | 84.52 | | 36.28 | | 51.01 | |
|  | 21 | | | 85.06 | | 79.15 | | 59.69 | | 70 | | | 84.51 | | 36.53 | | 50.68 | |
|  | 22 | | | 85.07 | | 74.33 | | 59.93 | | 71 | | | 84.49 | | 36.53 | | 50.7 | |
|  | 23 | | | 85.06 | | 69.1 | | 60.89 | | 72 | | | 84.48 | | 36.74 | | 50.7 | |
|  | 24 | | | 85.05 | | 63.59 | | 59.89 | | 73 | | | 84.47 | | 36.76 | | 51.09 | |
|  | 25 | | | 85.03 | | 63.59 | | 60.63 | | 74 | | | 84.44 | | 37.06 | | 51.37 | |
|  | 26 | | | 85 | | 58.34 | | 60.4 | | 75 | | | 84.42 | | 37.29 | | 51.37 | |
|  | 27 | | | 85 | | 54.31 | | 59.71 | | 76 | | | 84.4 | | 37.48 | | 51.39 | |
|  | 28 | | | 84.97 | | 51.21 | | 59.86 | | 77 | | | 84.38 | | 37.79 | | 51.52 | |
|  | 29 | | | 84.92 | | 48.91 | | 59.86 | | 78 | | | 84.4 | | 38.13 | | 51.77 | |
|  | 30 | | | 84.87 | | 47.11 | | 59.87 | | 79 | | | 84.39 | | 38.77 | | 51.56 | |
|  | 31 | | | 84.84 | | 45.31 | | 59.61 | | 80 | | | 84.4 | | 38.77 | | 51.85 | |
|  | 32 | | | 84.8 | | 43.67 | | 59.63 | | 81 | | | 84.4 | | 39.35 | | 51.85 | |
|  | 33 | | | 84.78 | | 42.18 | | 59.48 | | 82 | | | 84.4 | | 39.76 | | 51.87 | |
|  | 34 | | | 84.74 | | 40.65 | | 59.47 | | 83 | | | 84.37 | | 40.6 | | 52.57 | |
|  | 35 | | | 84.72 | | 40.65 | | 57.86 | | 84 | | | 84.35 | | 41.17 | | 52.1 | |
|  | 36 | | | 84.7 | | 39.11 | | 55.35 | | 85 | | | 84.31 | | 41.75 | | 52.06 | |
|  | 37 | | | 84.7 | | 38.14 | | 55.35 | | 86 | | | 84.26 | | 42.57 | | 52.49 | |
|  | 38 | | | 84.71 | | 37.06 | | 52.15 | | 87 | | | 83.97 | | 43.41 | | 52.47 | |
|  | 39 | | | 84.71 | | 36.26 | | 50.91 | | 88 | | | 83.91 | | 44.38 | | 52.29 | |
|  | 40 | | | 84.7 | | 35.63 | | 50.2 | | 89 | | | 83.56 | | 44.38 | | 52.29 | |
|  | 41 | | | 84.64 | | 35.16 | | 47.85 | | 90 | | | 83.16 | | 45.32 | | 52.58 | |
|  | 42 | | | 84.63 | | 35.18 | | 47.49 | | 91 | | | 82.66 | | 46.54 | | 52.58 | |
|  | 43 | | | 84.61 | | 35.12 | | 45.75 | | 92 | | | 82.11 | | 47.66 | | 52.37 | |
|  | 44 | | | 84.59 | | 35.12 | | 46.09 | | 93 | | | 81.23 | | 48.88 | | 52.53 | |
|  | 45 | | | 84.59 | | 35.17 | | 47.11 | | 94 | | | 80.9 | | 50.06 | | 52.88 | |
|  | 46 | | | 84.56 | | 35.03 | | 47.11 | | 95 | | | 80.5 | | 51.41 | | 53.1 | |
|  | 47 | | | 84.55 | | 35 | | 46.87 | | 96 | | | 80.46 | | 52.55 | | 53.77 | |
|  | 48 | | | 84.52 | | 34.62 | | 46.3 | | 97 | | | 80.15 | | 53.71 | | 53.77 | |
|  | 49 | | | 84.51 | | 34.3 | | 46.16 | | 98 | | | 80.09 | | 53.71 | | 53.77 | |
| g | 1 | | | 87.02 | | 86.66 | | 43.95 | | 100 | | | 80.07 | | 41.53 | | 41.19 | |
|  | 2 | | | 86.99 | | 85.35 | | 43.52 | | 101 | | | 79.71 | | 41.8 | | 41.63 | |
|  | 3 | | | 86.96 | | 85.35 | | 43.37 | | 102 | | | 79.38 | | 42.15 | | 42.4 | |
|  | 4 | | | 86.93 | | 83.58 | | 43.1 | | 103 | | | 78.93 | | 42.61 | | 42.4 | |
|  | 5 | | | 86.89 | | 81.07 | | 42.48 | | 104 | | | 78.54 | | 43.13 | | 43.44 | |
|  | 6 | | | 86.85 | | 78.41 | | 42.48 | | 105 | | | 78.22 | | 43.73 | | 42.76 | |
|  | 7 | | | 86.81 | | 75.88 | | 42.06 | | 106 | | | 78.15 | | 44.37 | | 42.36 | |
|  | 8 | | | 86.8 | | 73.32 | | 41.77 | | 107 | | | 77.9 | | 45.1 | | 41.22 | |
|  | 9 | | | 86.78 | | 71.02 | | 41.19 | | 108 | | | 77.89 | | 45.1 | | 40.78 | |
|  | 10 | | | 86.77 | | 68.33 | | 41.2 | | 109 | | | 77.88 | | 45.9 | | 40.3 | |
|  | 11 | | | 86.76 | | 64.78 | | 41.25 | | 110 | | | 77.8 | | 46.7 | | 40.64 | |
|  | 12 | | | 86.74 | | 57.75 | | 40.95 | | 111 | | | 77.56 | | 47.59 | | 40.65 | |
|  | 13 | | | 86.72 | | 52.96 | | 40.62 | | 112 | | | 77.47 | | 48.75 | | 40.65 | |
|  | 14 | | | 86.69 | | 52.96 | | 40.62 | | 113 | | | 77.18 | | 50.08 | | 40.36 | |
|  | 15 | | | 86.68 | | 49.67 | | 40.46 | | 114 | | | 76.9 | | 51.45 | | 39.92 | |
|  | 16 | | | 86.66 | | 48.54 | | 40.26 | | 115 | | | 76.86 | | 52.6 | | 39.92 | |
|  | 17 | | | 86.65 | | 48.05 | | 39.97 | | 116 | | | 76.6 | | 53.63 | | 39.92 | |
|  | 18 | | | 86.63 | | 48.05 | | 38.78 | | 117 | | | 76.53 | | 53.63 | | 39.67 | |
|  | 19 | | | 86.59 | | 48.55 | | 38.73 | | 118 | | | 76.26 | | 54.46 | | 39.66 | |
|  | 20 | | | 86.57 | | 49.22 | | 38.73 | | 119 | | | 75.83 | | 55.22 | | 39.53 | |
|  | 21 | | | 86.57 | | 49.9 | | 39.13 | | 120 | | | 75.77 | | 55.91 | | 39.53 | |
|  | 22 | | | 86.54 | | 50.49 | | 38.82 | | 121 | | | 75.51 | | 56.54 | | 39.69 | |
|  | 23 | | | 86.52 | | 50.49 | | 38.82 | | 122 | | | 75.44 | | 57.07 | | 39.57 | |
|  | 24 | | | 86.48 | | 50.91 | | 38.88 | | 123 | | | 75.36 | | 57.54 | | 39.71 | |
|  | 25 | | | 86.47 | | 51.04 | | 39.04 | | 124 | | | 75.36 | | 58.01 | | 40.09 | |
|  | 26 | | | 86.44 | | 51.05 | | 39.04 | | 125 | | | 75.4 | | 58.42 | | 39.86 | |
|  | 27 | | | 86.44 | | 51.27 | | 39.02 | | 126 | | | 75.65 | | 58.42 | | 39.97 | |
|  | 28 | | | 86.42 | | 51.25 | | 38.81 | | 127 | | | 75.98 | | 58.76 | | 39.5 | |
|  | 29 | | | 86.42 | | 51.42 | | 38.62 | | 128 | | | 76.06 | | 59.07 | | 39.5 | |
|  | 30 | | | 86.42 | | 51.42 | | 38.82 | | 129 | | | 76.38 | | 59.52 | | 39.29 | |
|  | 31 | | | 86.44 | | 51.46 | | 38.82 | | 130 | | | 76.39 | | 59.97 | | 39.59 | |
|  | 32 | | | 86.46 | | 51.57 | | 38.76 | | 131 | | | 76.68 | | 60.51 | | 39.62 | |
|  | 33 | | | 86.47 | | 51.57 | | 38.76 | | 132 | | | 76.71 | | 61.07 | | 39.6 | |
|  | 34 | | | 86.46 | | 51.61 | | 38.77 | | 133 | | | 76.73 | | 61.7 | | 39.37 | |
|  | 35 | | | 86.45 | | 51.62 | | 38.57 | | 134 | | | 76.7 | | 62.32 | | 38.95 | |
|  | 36 | | | 86.46 | | 51.63 | | 38.7 | | 135 | | | 76.41 | | 62.32 | | 38.95 | |
|  | 37 | | | 86.46 | | 51.48 | | 38.75 | | 136 | | | 76.01 | | 62.9 | | 38.95 | |
|  | 38 | | | 86.47 | | 51.26 | | 38.6 | | 137 | | | 75.95 | | 63.3 | | 39.1 | |
|  | 39 | | | 86.48 | | 51.12 | | 38.6 | | 138 | | | 75.85 | | 63.79 | | 39.46 | |
|  | 40 | | | 86.5 | | 50.96 | | 38.38 | | 139 | | | 75.63 | | 64.19 | | 39.17 | |
|  | 41 | | | 86.48 | | 50.96 | | 38.57 | | 140 | | | 75.63 | | 64.48 | | 39.04 | |
|  | 42 | | | 86.49 | | 50.96 | | 38.55 | | 141 | | | 75.62 | | 64.72 | | 39.04 | |
|  | 43 | | | 86.48 | | 50.97 | | 38.56 | | 142 | | | 75.61 | | 65 | | 38.7 | |
|  | 44 | | | 86.5 | | 51.2 | | 38.51 | | 143 | | | 75.56 | | 65.18 | | 38.54 | |
|  | 45 | | | 86.49 | | 51.33 | | 38.22 | | 144 | | | 75.5 | | 65.18 | | 38.54 | |
|  | 46 | | | 86.48 | | 51.36 | | 38.22 | | 145 | | | 75.28 | | 65.18 | | 38.69 | |
|  | 47 | | | 86.47 | | 51.37 | | 37.92 | | 146 | | | 75.27 | | 65.2 | | 38.69 | |
|  | 48 | | | 86.48 | | 51.51 | | 38.47 | | 147 | | | 75.26 | | 65.03 | | 38.68 | |
|  | 49 | | | 86.49 | | 51.51 | | 38.88 | | 148 | | | 75.28 | | 65.04 | | 38.99 | |
|  | 50 | | | 86.48 | | 51.51 | | 38.57 | | 149 | | | 75.32 | | 64.88 | | 40.02 | |
|  | 51 | | | 86.48 | | 51.37 | | 38.94 | | 150 | | | 75.39 | | 64.77 | | 40.4 | |
|  | 52 | | | 86.49 | | 51.37 | | 38.66 | | 151 | | | 75.48 | | 64.61 | | 40.4 | |
|  | 53 | | | 86.48 | | 51.14 | | 38.41 | | 152 | | | 75.58 | | 64.48 | | 40.4 | |
|  | 54 | | | 86.48 | | 50.85 | | 38.41 | | 153 | | | 75.66 | | 64.48 | | 40.76 | |
|  | 55 | | | 86.46 | | 50.32 | | 38.28 | | 154 | | | 75.73 | | 64.31 | | 40.75 | |
|  | 56 | | | 86.46 | | 49.42 | | 38.29 | | 155 | | | 75.97 | | 64.08 | | 40.47 | |
|  | 57 | | | 86.46 | | 48.54 | | 39.68 | | 156 | | | 76.01 | | 63.93 | | 40.48 | |
|  | 58 | | | 86.46 | | 47.6 | | 39.51 | | 157 | | | 76.08 | | 63.78 | | 41.14 | |
|  | 59 | | | 86.45 | | 46.63 | | 38.87 | | 158 | | | 76.12 | | 63.66 | | 41.02 | |
|  | 60 | | | 86.45 | | 45.45 | | 38.86 | | 159 | | | 75.88 | | 63.5 | | 41.31 | |
|  | 61 | | | 86.45 | | 45.45 | | 38.84 | | 160 | | | 75.84 | | 63.39 | | 41.49 | |
|  | 62 | | | 86.46 | | 44.28 | | 38.84 | | 161 | | | 75.81 | | 63.39 | | 41.49 | |
|  | 63 | | | 86.46 | | 43.19 | | 38.34 | | 162 | | | 75.74 | | 63.39 | | 42.28 | |
|  | 64 | | | 86.45 | | 42.21 | | 38.79 | | 163 | | | 75.72 | | 63.41 | | 42.04 | |
|  | 65 | | | 86.46 | | 41.25 | | 38.77 | | 164 | | | 75.78 | | 63.38 | | 42.05 | |
|  | 66 | | | 86.45 | | 40.37 | | 39.12 | | 165 | | | 75.82 | | 63.58 | | 42.05 | |
|  | 67 | | | 86.45 | | 39.8 | | 40.01 | | 166 | | | 75.9 | | 63.76 | | 42.04 | |
|  | 68 | | | 86.44 | | 39.23 | | 40.62 | | 167 | | | 75.97 | | 63.89 | | 42.06 | |
|  | 69 | | | 86.45 | | 38.95 | | 41.37 | | 168 | | | 76.06 | | 64.11 | | 42.54 | |
|  | 70 | | | 86.45 | | 38.95 | | 41.06 | | 169 | | | 76.12 | | 64.11 | | 42.54 | |
|  | 71 | | | 86.45 | | 38.89 | | 41.06 | | 170 | | | 76.16 | | 64.12 | | 42.53 | |
|  | 72 | | | 86.43 | | 38.91 | | 40.42 | | 171 | | | 76.18 | | 64.29 | | 42.19 | |
|  | 73 | | | 86.4 | | 38.94 | | 40.28 | | 172 | | | 76.17 | | 64.29 | | 42.2 | |
|  | 74 | | | 86.39 | | 39.23 | | 39.84 | | 173 | | | 76.1 | | 64.48 | | 41.4 | |
|  | 75 | | | 86.36 | | 39.58 | | 39.62 | | 174 | | | 76.05 | | 64.58 | | 41.72 | |
|  | 76 | | | 86.29 | | 39.92 | | 39.3 | | 175 | | | 76.03 | | 64.59 | | 41.86 | |
|  | 77 | | | 85.98 | | 40.38 | | 38.99 | | 176 | | | 76.04 | | 64.78 | | 41.6 | |
|  | 78 | | | 85.68 | | 40.91 | | 38.8 | | 177 | | | 76.07 | | 64.79 | | 41.6 | |
|  | 79 | | | 85.34 | | 40.91 | | 38.8 | | 178 | | | 76.12 | | 64.91 | | 41.31 | |
|  | 80 | | | 85.28 | | 41.47 | | 38.53 | | 179 | | | 76.37 | | 64.93 | | 41.09 | |
|  | 81 | | | 84.92 | | 42.04 | | 38.55 | | 180 | | | 76.43 | | 65.12 | | 41.74 | |
|  | 82 | | | 84.39 | | 42.54 | | 37.88 | | 181 | | | 76.71 | | 65.12 | | 41.48 | |
|  | 83 | | | 83.96 | | 42.95 | | 37.72 | | 182 | | | 76.78 | | 65.27 | | 41.24 | |
|  | 84 | | | 83.58 | | 43.15 | | 37.99 | | 183 | | | 77.02 | | 65.42 | | 41.55 | |
|  | 85 | | | 83.22 | | 43.26 | | 38.1 | | 184 | | | 77.02 | | 65.52 | | 41.54 | |
|  | 86 | | | 83.16 | | 43.28 | | 37.59 | | 185 | | | 77.01 | | 65.7 | | 41.3 | |
|  | 87 | | | 82.92 | | 43.27 | | 37.59 | | 186 | | | 76.97 | | 65.83 | | 41.3 | |
|  | 88 | | | 82.87 | | 43.07 | | 37.58 | | 187 | | | 76.96 | | 65.95 | | 41.52 | |
|  | 89 | | | 82.83 | | 43.07 | | 37.58 | | 188 | | | 76.94 | | 66.09 | | 41.21 | |
|  | 90 | | | 82.64 | | 42.79 | | 37.58 | | 189 | | | 76.98 | | 66.24 | | 41.04 | |
|  | 91 | | | 82.57 | | 42.47 | | 37.77 | | 190 | | | 77.23 | | 66.24 | | 40.73 | |
|  | 92 | | | 82.26 | | 42.18 | | 37.74 | | 191 | | | 77.29 | | 66.39 | | 41.04 | |
|  | 93 | | | 82.22 | | 41.88 | | 37.87 | | 192 | | | 77.72 | | 66.54 | | 40.52 | |
|  | 94 | | | 82.15 | | 41.57 | | 38.38 | | 193 | | | 77.71 | | 66.72 | | 40.36 | |
|  | 95 | | | 81.89 | | 41.37 | | 38.38 | | 194 | | | 77.75 | | 66.92 | | 40.36 | |
|  | 96 | | | 81.56 | | 41.23 | | 38.37 | | 195 | | | 77.81 | | 67.14 | | 40.61 | |
|  | 97 | | | 81.25 | | 41.24 | | 39.32 | | 196 | | | 77.89 | | 67.15 | | 40.61 | |
|  | 98 | | | 80.82 | | 41.24 | | 39.79 | | 197 | | | 77.94 | | 67.39 | | 40.65 | |
|  | 99 | | | 80.47 | | 41.25 | | 40.04 | | 198 | | | 77.99 | | 67.53 | | 41.28 | |
| h | 1 | | | 86.9 | | 83.41 | | 51.56 | | 50 | | | 87.15 | | 29.62 | | 46.32 | |
|  | 2 | | | 86.97 | | 83.47 | | 52.28 | | 51 | | | 87.12 | | 29.99 | | 46.44 | |
|  | 3 | | | 87.28 | | 83.47 | | 53.46 | | 52 | | | 87.05 | | 30.37 | | 46.45 | |
|  | 4 | | | 87.34 | | 83.5 | | 53.63 | | 53 | | | 87.01 | | 30.74 | | 46.68 | |
|  | 5 | | | 87.43 | | 83.44 | | 53.26 | | 54 | | | 86.93 | | 31.17 | | 48.19 | |
|  | 6 | | | 87.66 | | 83.42 | | 51.24 | | 55 | | | 86.86 | | 31.6 | | 46.78 | |
|  | 7 | | | 87.72 | | 83.29 | | 51.24 | | 56 | | | 86.75 | | 31.95 | | 46.51 | |
|  | 8 | | | 87.93 | | 82.46 | | 50.22 | | 57 | | | 86.67 | | 31.95 | | 46.51 | |
|  | 9 | | | 87.97 | | 81.03 | | 49.33 | | 58 | | | 86.63 | | 32.2 | | 45.57 | |
|  | 10 | | | 88.35 | | 79.54 | | 49.45 | | 59 | | | 86.58 | | 32.77 | | 45.2 | |
|  | 11 | | | 88.79 | | 79.15 | | 48.32 | | 60 | | | 86.53 | | 33.07 | | 45.86 | |
|  | 12 | | | 89.1 | | 79.15 | | 46.39 | | 61 | | | 86.48 | | 33.6 | | 46.4 | |
|  | 13 | | | 89.14 | | 78.29 | | 46.38 | | 62 | | | 86.45 | | 33.96 | | 45.87 | |
|  | 14 | | | 89.18 | | 76.11 | | 46.38 | | 63 | | | 86.43 | | 34.38 | | 46.14 | |
|  | 15 | | | 89.21 | | 73.66 | | 45.06 | | 64 | | | 86.4 | | 34.78 | | 46.32 | |
|  | 16 | | | 89.24 | | 71.36 | | 43.92 | | 65 | | | 86.34 | | 35.05 | | 46.08 | |
|  | 17 | | | 89.26 | | 68.97 | | 42.88 | | 66 | | | 86.29 | | 35.05 | | 46.08 | |
|  | 18 | | | 88.9 | | 66.54 | | 43.43 | | 67 | | | 86.21 | | 35.41 | | 46.99 | |
|  | 19 | | | 88.4 | | 63.98 | | 41.96 | | 68 | | | 86.19 | | 35.95 | | 46.35 | |
|  | 20 | | | 88.06 | | 61.67 | | 41.48 | | 69 | | | 86.17 | | 36.36 | | 46.94 | |
|  | 21 | | | 87.73 | | 61.67 | | 40.61 | | 70 | | | 86.2 | | 36.84 | | 47.29 | |
|  | 22 | | | 87.67 | | 59.03 | | 39.97 | | 71 | | | 86.23 | | 37.32 | | 47.26 | |
|  | 23 | | | 87.58 | | 56.64 | | 39.97 | | 72 | | | 86.24 | | 37.84 | | 46.76 | |
|  | 24 | | | 87.51 | | 54.15 | | 41 | | 73 | | | 86.24 | | 38.4 | | 46.36 | |
|  | 25 | | | 87.79 | | 51.46 | | 41.53 | | 74 | | | 86.23 | | 38.91 | | 46.36 | |
|  | 26 | | | 87.82 | | 49.15 | | 39.86 | | 75 | | | 86.19 | | 38.91 | | 46.79 | |
|  | 27 | | | 88.19 | | 46.77 | | 40.54 | | 76 | | | 86.17 | | 39.74 | | 47.58 | |
|  | 28 | | | 88.2 | | 44.39 | | 41.33 | | 77 | | | 86.16 | | 40.62 | | 47.43 | |
|  | 29 | | | 88.24 | | 42.14 | | 42.27 | | 78 | | | 86.14 | | 41.39 | | 47.16 | |
|  | 30 | | | 88.27 | | 42.14 | | 40.46 | | 79 | | | 86.08 | | 42.44 | | 47.4 | |
|  | 31 | | | 88.31 | | 40.23 | | 41.43 | | 80 | | | 85.75 | | 43.33 | | 47.87 | |
|  | 32 | | | 88.36 | | 38.43 | | 41.43 | | 81 | | | 85.4 | | 44.4 | | 47.51 | |
|  | 33 | | | 88.51 | | 36.59 | | 41.43 | | 82 | | | 85.36 | | 45.36 | | 46.84 | |
|  | 34 | | | 88.57 | | 35.33 | | 41.25 | | 83 | | | 85.01 | | 46.62 | | 46.84 | |
|  | 35 | | | 88.57 | | 34.06 | | 43.11 | | 84 | | | 84.94 | | 46.62 | | 47.16 | |
|  | 36 | | | 88.31 | | 32.98 | | 43.73 | | 85 | | | 84.56 | | 47.71 | | 47.01 | |
|  | 37 | | | 88.27 | | 32.03 | | 43.74 | | 86 | | | 84.53 | | 48.95 | | 46.63 | |
|  | 38 | | | 88 | | 31.07 | | 43.45 | | 87 | | | 84.28 | | 49.99 | | 46.65 | |
|  | 39 | | | 88 | | 31.07 | | 44.13 | | 88 | | | 84.24 | | 51.2 | | 47.06 | |
|  | 40 | | | 87.97 | | 30.46 | | 44.13 | | 89 | | | 83.91 | | 52.34 | | 46.53 | |
|  | 41 | | | 87.91 | | 29.85 | | 43.95 | | 90 | | | 83.82 | | 53.46 | | 46.38 | |
|  | 42 | | | 87.63 | | 29.52 | | 44.75 | | 91 | | | 83.42 | | 54.58 | | 46.38 | |
|  | 43 | | | 87.61 | | 29.3 | | 44.77 | | 92 | | | 83.13 | | 55.88 | | 46.11 | |
|  | 44 | | | 87.56 | | 29.14 | | 45.03 | | 93 | | | 83.09 | | 55.88 | | 46.39 | |
|  | 45 | | | 87.48 | | 29.06 | | 45.33 | | 94 | | | 82.72 | | 56.98 | | 46.41 | |
|  | 46 | | | 87.29 | | 29.15 | | 45.31 | | 95 | | | 82.34 | | 58.06 | | 47.25 | |
|  | 47 | | | 87.27 | | 29.15 | | 45.71 | | 96 | | | 81.97 | | 59.14 | | 47.36 | |
|  | 48 | | | 87.23 | | 29.16 | | 45.56 | | 97 | | | 81.49 | | 60.27 | | 47.79 | |
|  | 49 | | | 87.2 | | 29.34 | | 45.56 | | 98 | | | 81.17 | | 61.42 | | 47.45 | |
| i | 1 | | | 91.73 | | 74.16 | | 53.04 | | 50 | | | 94.11 | | 30.73 | | 45.58 | |
|  | 2 | | | 91.69 | | 74.11 | | 52.89 | | 51 | | | 94.36 | | 31.53 | | 45.13 | |
|  | 3 | | | 91.67 | | 74.32 | | 53.10 | | 52 | | | 94.41 | | 32.35 | | 43.03 | |
|  | 4 | | | 91.72 | | 74.23 | | 53.10 | | 53 | | | 94.48 | | 33.26 | | 42.59 | |
|  | 5 | | | 91.96 | | 74.46 | | 52.58 | | 54 | | | 94.55 | | 34.05 | | 44.83 | |
|  | 6 | | | 92.00 | | 74.46 | | 50.99 | | 55 | | | 94.56 | | 34.99 | | 44.83 | |
|  | 7 | | | 92.06 | | 74.54 | | 49.35 | | 56 | | | 94.56 | | 35.93 | | 44.5 | |
|  | 8 | | | 92.09 | | 74.66 | | 48.21 | | 57 | | | 94.56 | | 36.79 | | 45.04 | |
|  | 9 | | | 92.05 | | 73.63 | | 48.75 | | 58 | | | 94.55 | | 37.51 | | 45.64 | |
|  | 10 | | | 92.03 | | 71.87 | | 48.47 | | 59 | | | 94.54 | | 37.51 | | 46.47 | |
|  | 11 | | | 91.98 | | 66.79 | | 48.93 | | 60 | | | 94.54 | | 38.19 | | 46.08 | |
|  | 12 | | | 92.02 | | 60.27 | | 48.93 | | 61 | | | 94.56 | | 38.83 | | 45.78 | |
|  | 13 | | | 92.06 | | 55.06 | | 48.89 | | 62 | | | 94.6 | | 39.33 | | 46.33 | |
|  | 14 | | | 92.15 | | 52.20 | | 48.53 | | 63 | | | 94.65 | | 39.82 | | 47.91 | |
|  | 15 | | | 92.20 | | 52.20 | | 44.61 | | 64 | | | 94.72 | | 40.40 | | 47.91 | |
|  | 16 | | | 92.24 | | 50.29 | | 42.59 | | 65 | | | 94.71 | | 40.85 | | 47.54 | |
|  | 17 | | | 92.26 | | 48.59 | | 41.6 | | 66 | | | 94.64 | | 41.13 | | 46.72 | |
|  | 18 | | | 92.30 | | 46.88 | | 42.18 | | 67 | | | 94.44 | | 41.54 | | 47.08 | |
|  | 19 | | | 92.33 | | 45.59 | | 39.94 | | 68 | | | 94.39 | | 41.54 | | 48.92 | |
|  | 20 | | | 92.34 | | 44.46 | | 39.94 | | 69 | | | 94.1 | | 42.06 | | 48.21 | |
|  | 21 | | | 92.39 | | 42.67 | | 40.17 | | 70 | | | 93.68 | | 42.5 | | 46.65 | |
|  | 22 | | | 92.45 | | 41.03 | | 40.62 | | 71 | | | 93.32 | | 42.96 | | 48.69 | |
|  | 23 | | | 92.49 | | 39.5 | | 39.45 | | 72 | | | 93 | | 43.44 | | 48.69 | |
|  | 24 | | | 92.52 | | 39.5 | | 41.27 | | 73 | | | 92.92 | | 44.08 | | 49.53 | |
|  | 25 | | | 92.76 | | 37.96 | | 41.61 | | 74 | | | 92.64 | | 44.58 | | 49.81 | |
|  | 26 | | | 92.74 | | 36.46 | | 41.41 | | 75 | | | 92.57 | | 45.35 | | 48.47 | |
|  | 27 | | | 92.75 | | 34.79 | | 42.33 | | 76 | | | 92.30 | | 45.97 | | 49.46 | |
|  | 28 | | | 92.75 | | 33.25 | | 41.9 | | 77 | | | 92.23 | | 45.97 | | 49.09 | |
|  | 29 | | | 92.78 | | 32.05 | | 41.9 | | 78 | | | 91.88 | | 46.73 | | 48.63 | |
|  | 30 | | | 92.83 | | 31.41 | | 42.18 | | 79 | | | 91.82 | | 47.54 | | 47.90 | |
|  | 31 | | | 92.85 | | 30.8 | | 42.54 | | 80 | | | 91.52 | | 48.47 | | 48.48 | |
|  | 32 | | | 92.88 | | 30.11 | | 42.77 | | 81 | | | 91.46 | | 49.19 | | 48.48 | |
|  | 33 | | | 92.92 | | 30.11 | | 43.28 | | 82 | | | 91.17 | | 50.35 | | 49.76 | |
|  | 34 | | | 92.97 | | 29.52 | | 44.1 | | 83 | | | 91.12 | | 51.36 | | 48.73 | |
|  | 35 | | | 92.98 | | 29.09 | | 44.11 | | 84 | | | 90.68 | | 52.51 | | 49.16 | |
|  | 36 | | | 92.98 | | 28.83 | | 43.54 | | 85 | | | 89.79 | | 53.58 | | 48.67 | |
|  | 37 | | | 93.00 | | 28.69 | | 44.02 | | 86 | | | 89.18 | | 53.58 | | 48.65 | |
|  | 38 | | | 93.04 | | 28.43 | | 44.02 | | 87 | | | 88.76 | | 54.77 | | 48.66 | |
|  | 39 | | | 93.11 | | 28.42 | | 44.02 | | 88 | | | 88.31 | | 55.6 | | 48.38 | |
|  | 40 | | | 93.36 | | 28.44 | | 44.15 | | 89 | | | 87.79 | | 56.73 | | 48.38 | |
|  | 41 | | | 93.39 | | 28.33 | | 44.66 | | 90 | | | 87.74 | | 57.85 | | 49.32 | |
|  | 42 | | | 93.45 | | 28.33 | | 45.06 | | 91 | | | 87.33 | | 58.72 | | 48.98 | |
|  | 43 | | | 93.50 | | 28.48 | | 45.43 | | 92 | | | 87.00 | | 59.79 | | 48.65 | |
|  | 44 | | | 93.56 | | 28.58 | | 45.76 | | 93 | | | 86.57 | | 61.01 | | 48.14 | |
|  | 45 | | | 93.91 | | 29.05 | | 46.03 | | 94 | | | 86.53 | | 62.42 | | 47.56 | |
|  | 46 | | | 93.91 | | 29.29 | | 45.39 | | 95 | | | 86.21 | | 62.42 | | 47.81 | |
|  | 47 | | | 93.96 | | 29.66 | | 45.39 | | 96 | | | 85.77 | | 63.59 | | 49.29 | |
|  | 48 | | | 94.00 | | 30.3 | | 46.14 | | 97 | | | 85.32 | | 64.57 | | 49.29 | |
|  | 49 | | | 94.05 | | 30.73 | | 45.96 | | 98 | | | 84.84 | | 65.39 | | 49.16 | |

a. The thermal medium flow rate (TMFR) was 30 L/min, the scraper speed (SS) was 0 r/min, the snow completely melted time (SCMT) was 178 s; b. The TMFR was 30 L/min, the SS was 25 r/min, the SCMT time was 19 s; c. The TMFR was 30 L/min, the SS was 42 r/min, the SCMT was 7 s; d. The TMFR was 40 L/min, the SS was 0 r/min, the SCMT was 176 s; e. The TMFR was 40 L/min, the SS was 25 r/min, the SCMT was 13 s; f. The TMFR was 40 L/min, the SS was 42 r/min, the SCMT was 7 s; g. The TMFR was 60 L/min, the SS was 0 r/min, the SCMT was 148 s; h. The TMFR was 60 L/min, the SS was 25 r/min, the SCMT was 13 s; i. The TMFR was 60 L/min, the SS was 42 r/min, the SCMT was 6 s.
