# Supplementary material for: Design and performance optimization of vehicle-mounted thermal snow-melting system
Source: PLoS One. 2025 Mar 13;20(3):e0317957. doi: 10.1371/journal.pone.0317957 (PMC11906039; doi:10.1371/journal.pone.0317957)
Supplement: S3 Table — (DOCX) [file pone.0317957.s003.docx]

**S3 Table. Fig 4 data.**

|  | Time/s | Inlet water/℃ | Outlet water/℃ | Inside the box/℃ | Time/s | Inlet water /℃ | Outlet water/℃ | Inside the box/℃ |
| --- | --- | --- | --- | --- | --- | --- | --- | --- |
| a | 1 | 89.22 | 84.17 | 58.98 | 150 | 76.08 | 63.32 | 55.24 |
|  | 2 | 89.22 | 84.39 | 58.98 | 151 | 76.13 | 62.95 | 55.33 |
|  | 3 | 89.2 | 84.63 | 58.97 | 152 | 76.2 | 62.93 | 55.33 |
|  | 4 | 89.18 | 84.82 | 59.26 | 153 | 76.27 | 62.69 | 55.19 |
|  | 5 | 89.21 | 85.21 | 59.07 | 154 | 76.36 | 62.55 | 55.18 |
|  | 6 | 89.26 | 85.45 | 58.96 | 155 | 76.49 | 62.35 | 54.54 |
|  | 7 | 89.38 | 85.45 | 58.83 | 156 | 76.55 | 62.18 | 54.2 |
|  | 8 | 89.45 | 85.64 | 58.62 | 157 | 76.62 | 61.96 | 53.43 |
|  | 9 | 89.46 | 85.75 | 58.62 | 158 | 76.9 | 61.74 | 53.14 |
|  | 10 | 89.44 | 86.17 | 58.64 | 159 | 76.9 | 61.74 | 53.01 |
|  | 11 | 89.43 | 86.36 | 58.82 | 160 | 76.93 | 61.66 | 53 |
|  | 12 | 89.43 | 86.56 | 58.8 | 161 | 76.98 | 61.56 | 53 |
|  | 13 | 89.46 | 86.99 | 58.87 | 162 | 77 | 61.38 | 52.76 |
|  | 14 | 89.52 | 87.3 | 58.9 | 163 | 77.03 | 61.35 | 53.25 |
|  | 15 | 89.58 | 87.49 | 59.09 | 164 | 77.04 | 61.39 | 53.58 |
|  | 16 | 89.65 | 87.49 | 59.09 | 165 | 77.09 | 61.38 | 53.79 |
|  | 17 | 89.66 | 87.73 | 59.09 | 166 | 77.13 | 61.48 | 53.77 |
|  | 18 | 89.63 | 88.06 | 59.1 | 167 | 77.22 | 61.67 | 53.78 |
|  | 19 | 89.63 | 88.17 | 59.11 | 168 | 77.26 | 61.67 | 53.81 |
|  | 20 | 89.55 | 88.56 | 59.09 | 169 | 77.35 | 61.81 | 53.81 |
|  | 21 | 89.35 | 88.88 | 59.09 | 170 | 77.56 | 61.88 | 53.83 |
|  | 22 | 89.35 | 89.03 | 59.01 | 171 | 77.6 | 61.87 | 53.85 |
|  | 23 | 89.36 | 88.9 | 58.85 | 172 | 77.64 | 61.82 | 53.93 |
|  | 24 | 89.36 | 89.1 | 58.74 | 173 | 77.9 | 61.73 | 53.79 |
|  | 25 | 89.32 | 89.1 | 58.74 | 174 | 77.93 | 61.77 | 53.79 |
|  | 26 | 89.03 | 88.82 | 58.93 | 175 | 77.98 | 61.83 | 53.14 |
|  | 27 | 88.99 | 87.68 | 59.21 | 176 | 78.25 | 61.73 | 52.27 |
|  | 28 | 88.93 | 86.73 | 59.02 | 177 | 78.29 | 61.73 | 52.25 |
|  | 29 | 88.64 | 86.09 | 59.05 | 178 | 78.54 | 61.54 | 52.25 |
|  | 30 | 88.31 | 85.82 | 58.99 | 179 | 78.57 | 61.15 | 51.97 |
|  | 31 | 88.29 | 85.17 | 58.99 | 180 | 78.62 | 60.55 | 51.88 |
|  | 32 | 88.21 | 82 | 58.99 | 181 | 78.85 | 60.16 | 51.87 |
|  | 33 | 87.95 | 79.82 | 59 | 182 | 78.91 | 59.53 | 51.98 |
|  | 34 | 87.87 | 79.82 | 58.84 | 183 | 79.2 | 58.94 | 52.18 |
|  | 35 | 87.59 | 77.2 | 58.84 | 184 | 79.25 | 58.18 | 52.44 |
|  | 36 | 87.3 | 76.11 | 58.63 | 185 | 79.51 | 57.75 | 52.45 |
|  | 37 | 87.32 | 75.13 | 58.64 | 186 | 79.53 | 57.75 | 52.45 |
|  | 38 | 87.27 | 72.93 | 59.19 | 187 | 79.79 | 57.11 | 52.45 |
|  | 39 | 87.22 | 71.62 | 59.45 | 188 | 79.83 | 56.6 | 52.44 |
|  | 40 | 87.18 | 70.97 | 59.45 | 189 | 80.14 | 56.36 | 52.63 |
|  | 41 | 87.17 | 69.84 | 59.28 | 190 | 80.17 | 55.88 | 52.63 |
|  | 42 | 87.17 | 68.03 | 59.05 | 191 | 80.48 | 55.78 | 52.64 |
|  | 43 | 87.15 | 68.03 | 59.02 | 192 | 80.53 | 55.57 | 52.84 |
|  | 44 | 87.19 | 66.6 | 59.35 | 193 | 80.79 | 55.31 | 52.84 |
|  | 45 | 87.19 | 65.4 | 59.64 | 194 | 80.85 | 55.26 | 52.83 |
|  | 46 | 87.23 | 64.02 | 59.64 | 195 | 80.93 | 55.26 | 52.83 |
|  | 47 | 87.22 | 62.31 | 59.88 | 196 | 81.22 | 55.29 | 52.8 |
|  | 48 | 87.24 | 60.44 | 59.88 | 197 | 81.23 | 55.27 | 52.06 |
|  | 49 | 87.22 | 59.5 | 59.77 | 198 | 81.32 | 55.17 | 51.7 |
|  | 50 | 87.2 | 58.86 | 59.78 | 199 | 81.58 | 55.23 | 51.23 |
|  | 51 | 87.19 | 58.36 | 59.87 | 200 | 81.62 | 55.17 | 51.23 |
|  | 52 | 87.13 | 58.36 | 58.94 | 201 | 81.69 | 55.33 | 51.05 |
|  | 53 | 87.03 | 57.69 | 57.75 | 202 | 82.01 | 55.32 | 51.04 |
|  | 54 | 86.81 | 56.97 | 57.07 | 203 | 82.07 | 55.16 | 51.21 |
|  | 55 | 86.79 | 56.15 | 56.53 | 204 | 82.33 | 55.16 | 51.21 |
|  | 56 | 86.5 | 55.3 | 56.53 | 205 | 82.4 | 55.09 | 51.19 |
|  | 57 | 86.43 | 54.34 | 56.08 | 206 | 82.71 | 54.96 | 51.19 |
|  | 58 | 86.22 | 53.43 | 55.52 | 207 | 82.74 | 54.73 | 51.18 |
|  | 59 | 86.2 | 52.59 | 54.78 | 208 | 83.05 | 54.45 | 51.18 |
|  | 60 | 86.18 | 52.59 | 53.83 | 209 | 83.06 | 54.48 | 51.31 |
|  | 61 | 86.08 | 51.9 | 53.69 | 210 | 83.14 | 54.46 | 51.52 |
|  | 62 | 85.85 | 51.57 | 53.48 | 211 | 83.21 | 54.14 | 51.5 |
|  | 63 | 85.82 | 51.23 | 53.65 | 212 | 83.42 | 54.2 | 51.5 |
|  | 64 | 85.8 | 51.05 | 53.65 | 213 | 83.47 | 54.2 | 51.64 |
|  | 65 | 85.76 | 50.94 | 53.35 | 214 | 83.53 | 54.18 | 51.79 |
|  | 66 | 85.74 | 50.97 | 52.77 | 215 | 83.81 | 54.17 | 51.78 |
|  | 67 | 85.73 | 51.06 | 52.34 | 216 | 84.07 | 54.18 | 51.49 |
|  | 68 | 85.74 | 51.01 | 51.86 | 217 | 84.12 | 54.22 | 51.26 |
|  | 69 | 85.76 | 51.14 | 51.48 | 218 | 84.41 | 54.6 | 51.11 |
|  | 70 | 85.8 | 51.14 | 50.96 | 219 | 84.48 | 54.84 | 51.27 |
|  | 71 | 85.81 | 51.11 | 50.02 | 220 | 84.53 | 55.13 | 51.27 |
|  | 72 | 85.84 | 51.13 | 50.32 | 221 | 84.61 | 55.55 | 51.27 |
|  | 73 | 85.82 | 51.05 | 50.32 | 222 | 84.85 | 55.55 | 51.28 |
|  | 74 | 85.78 | 51.11 | 50.35 | 223 | 84.9 | 56.04 | 51.59 |
|  | 75 | 85.77 | 51.13 | 50.36 | 224 | 85.19 | 56.41 | 51.73 |
|  | 76 | 85.72 | 50.93 | 50.18 | 225 | 85.21 | 56.72 | 51.98 |
|  | 77 | 85.72 | 50.91 | 50.18 | 226 | 85.26 | 57.16 | 52 |
|  | 78 | 85.67 | 50.69 | 51.21 | 227 | 85.35 | 57.45 | 52.03 |
|  | 79 | 85.65 | 50.69 | 51.82 | 228 | 85.56 | 57.8 | 52.26 |
|  | 80 | 85.63 | 50.42 | 52.3 | 229 | 85.65 | 58.11 | 52.25 |
|  | 81 | 85.58 | 50.08 | 51.84 | 230 | 86.02 | 58.47 | 52.25 |
|  | 82 | 85.53 | 49.61 | 51.84 | 231 | 86.02 | 58.47 | 52.49 |
|  | 83 | 85.53 | 49.19 | 52.1 | 232 | 86.03 | 58.91 | 52.69 |
|  | 84 | 85.53 | 49 | 52.35 | 233 | 86.11 | 59.27 | 52.69 |
|  | 85 | 85.49 | 48.64 | 52.54 | 234 | 86.14 | 59.56 | 52.83 |
|  | 86 | 85.46 | 48.66 | 52.54 | 235 | 86.18 | 60 | 52.82 |
|  | 87 | 85.42 | 48.58 | 52.55 | 236 | 86.21 | 60.41 | 52.82 |
|  | 88 | 85.36 | 48.58 | 52.67 | 237 | 86.29 | 60.75 | 52.8 |
|  | 89 | 85.33 | 48.78 | 52.67 | 238 | 86.46 | 61.12 | 52.8 |
|  | 90 | 85.28 | 49.03 | 52.84 | 239 | 86.53 | 61.63 | 52.99 |
|  | 91 | 85.27 | 49.43 | 52.84 | 240 | 86.61 | 61.63 | 53 |
|  | 92 | 85.24 | 50.01 | 53.03 | 241 | 86.66 | 61.78 | 53 |
|  | 93 | 85.2 | 50.69 | 52.41 | 242 | 86.69 | 62.31 | 53.02 |
|  | 94 | 85.14 | 51.51 | 53.12 | 243 | 86.66 | 62.6 | 53.11 |
|  | 95 | 85.1 | 52.27 | 53.44 | 244 | 86.66 | 62.73 | 53.13 |
|  | 96 | 85.08 | 53.39 | 53.63 | 245 | 86.71 | 63.02 | 53.13 |
|  | 97 | 85.09 | 53.39 | 53.94 | 246 | 87 | 63.27 | 53.13 |
|  | 98 | 85.09 | 54.33 | 54.32 | 247 | 87.01 | 63.56 | 53.14 |
|  | 99 | 85.08 | 55.41 | 54.32 | 248 | 87.09 | 63.7 | 53.24 |
|  | 100 | 85.08 | 56.6 | 54.47 | 249 | 87.17 | 63.7 | 53.24 |
|  | 101 | 85.05 | 57.64 | 54.49 | 250 | 87.25 | 63.71 | 53 |
|  | 102 | 84.99 | 58.58 | 54.06 | 251 | 87.26 | 63.97 | 53.01 |
|  | 103 | 84.75 | 59.42 | 53.86 | 252 | 87.23 | 64.13 | 53.05 |
|  | 104 | 84.69 | 60.2 | 53.4 | 253 | 87.24 | 64.21 | 53.03 |
|  | 105 | 84.41 | 60.78 | 53.2 | 254 | 87.29 | 64.47 | 53.03 |
|  | 106 | 84.11 | 60.78 | 53.57 | 255 | 87.38 | 64.63 | 53.03 |
|  | 107 | 84.05 | 61.36 | 53.99 | 256 | 87.45 | 64.74 | 53.02 |
|  | 108 | 83.75 | 61.9 | 53.99 | 257 | 87.69 | 65.1 | 53.13 |
|  | 109 | 83.45 | 62.33 | 54.11 | 258 | 87.71 | 65.1 | 53.25 |
|  | 110 | 83.36 | 62.66 | 54.12 | 259 | 87.75 | 65.45 | 53.07 |
|  | 111 | 83.07 | 62.89 | 54.23 | 260 | 87.89 | 65.8 | 53.07 |
|  | 112 | 82.75 | 63.11 | 54.23 | 261 | 87.94 | 65.99 | 53.06 |
|  | 113 | 82.67 | 63.59 | 54.24 | 262 | 87.96 | 66.36 | 53.06 |
|  | 114 | 82.31 | 63.85 | 54.26 | 263 | 87.95 | 66.93 | 53.06 |
|  | 115 | 82.25 | 63.85 | 53.93 | 264 | 87.94 | 67.28 | 53.06 |
|  | 116 | 82 | 64.05 | 54.12 | 265 | 87.98 | 67.7 | 53.18 |
|  | 117 | 81.96 | 64.41 | 54.12 | 266 | 87.98 | 68.15 | 53.32 |
|  | 118 | 81.91 | 64.48 | 54.35 | 267 | 88.03 | 68.15 | 53.32 |
|  | 119 | 81.7 | 64.78 | 54.04 | 268 | 88 | 68.49 | 53.19 |
|  | 120 | 81.68 | 65.02 | 54.04 | 269 | 88.04 | 68.89 | 53.2 |
|  | 121 | 81.62 | 65.2 | 54.23 | 270 | 88.02 | 69.32 | 53.39 |
|  | 122 | 81.41 | 65.4 | 54.22 | 271 | 88.02 | 69.66 | 53.42 |
|  | 123 | 81.35 | 65.55 | 54.07 | 272 | 87.98 | 69.92 | 53.42 |
|  | 124 | 81.08 | 65.55 | 54.05 | 273 | 87.96 | 70.18 | 53.4 |
|  | 125 | 81.06 | 65.63 | 54.24 | 274 | 87.94 | 70.62 | 53.23 |
|  | 126 | 81.02 | 65.96 | 54.24 | 275 | 87.91 | 70.84 | 53.24 |
|  | 127 | 80.83 | 65.95 | 54.25 | 276 | 87.91 | 70.84 | 53.35 |
|  | 128 | 80.77 | 66.16 | 54.53 | 277 | 87.88 | 71.02 | 54.05 |
|  | 129 | 80.4 | 66.03 | 54.51 | 278 | 87.84 | 71.3 | 54.04 |
|  | 130 | 80.31 | 66.13 | 54.53 | 279 | 87.85 | 71.66 | 54.22 |
|  | 131 | 80.05 | 66.21 | 54.04 | 280 | 87.89 | 72.07 | 54.22 |
|  | 132 | 79.96 | 66.21 | 54.51 | 281 | 87.95 | 72.44 | 54.31 |
|  | 133 | 79.67 | 66.06 | 54.88 | 282 | 87.99 | 72.73 | 54.6 |
|  | 134 | 79.36 | 66.02 | 55.02 | 283 | 88 | 73.11 | 55.1 |
|  | 135 | 79.05 | 65.89 | 55.02 | 284 | 88 | 73.42 | 55.32 |
|  | 136 | 79 | 65.73 | 54.9 | 285 | 87.97 | 73.42 | 55.33 |
|  | 137 | 78.7 | 65.62 | 54.9 | 286 | 88 | 73.83 | 55.18 |
|  | 138 | 78.66 | 65.59 | 54.86 | 287 | 88.02 | 74.14 | 55.3 |
|  | 139 | 78.41 | 65.36 | 55.07 | 288 | 88.01 | 74.52 | 55.28 |
|  | 140 | 78.35 | 65.06 | 55.19 | 289 | 88.04 | 74.79 | 55.28 |
|  | 141 | 78.02 | 65.06 | 55.18 | 290 | 88.02 | 75.21 | 55.45 |
|  | 142 | 77.61 | 64.74 | 55.18 | 291 | 88.02 | 75.42 | 55.26 |
|  | 143 | 77.19 | 64.38 | 55.18 | 292 | 88.03 | 75.78 | 55.23 |
|  | 144 | 76.86 | 63.97 | 55.28 | 293 | 88.07 | 76.04 | 54.93 |
|  | 145 | 76.83 | 63.84 | 55.49 | 294 | 88.14 | 76.04 | 54.92 |
|  | 146 | 76.46 | 63.8 | 55.48 | 295 | 88.2 | 76.33 | 54.78 |
|  | 147 | 76.15 | 63.7 | 55.39 | 296 | 88.24 | 76.55 | 54.59 |
|  | 148 | 76.11 | 63.49 | 55.38 | 297 | 88.24 | 76.92 | 54.59 |
|  | 149 | 76.08 | 63.32 | 55.38 | 298 | 88.25 | 77.03 | 54.58 |
| b | 1 | 86.78 | 87.66 | 58.43 | 100 | 82 | 43.23 | 46.97 |
|  | 2 | 86.77 | 87.66 | 58.28 | 101 | 81.95 | 43.23 | 47.82 |
|  | 3 | 86.78 | 87.65 | 57.88 | 102 | 81.68 | 44.32 | 47.21 |
|  | 4 | 86.78 | 87.57 | 57.89 | 103 | 81.63 | 45.23 | 47.87 |
|  | 5 | 86.79 | 87.43 | 58.05 | 104 | 81.29 | 45.94 | 48.48 |
|  | 6 | 86.78 | 87.22 | 58.21 | 105 | 81.27 | 46.71 | 47.99 |
|  | 7 | 86.79 | 86.92 | 58.39 | 106 | 81.2 | 47.57 | 47.68 |
|  | 8 | 86.77 | 86.59 | 58.25 | 107 | 81.1 | 48.27 | 47.43 |
|  | 9 | 86.76 | 86.05 | 58.25 | 108 | 81.08 | 48.94 | 47.42 |
|  | 10 | 86.76 | 85.72 | 58.13 | 109 | 81 | 49.58 | 47.42 |
|  | 11 | 86.76 | 85.72 | 58.15 | 110 | 80.96 | 49.58 | 47.49 |
|  | 12 | 86.74 | 85.17 | 58.56 | 111 | 80.9 | 50.22 | 47.16 |
|  | 13 | 86.68 | 84.92 | 58.96 | 112 | 80.88 | 50.79 | 46.79 |
|  | 14 | 86.63 | 84.31 | 58.71 | 113 | 80.81 | 51.62 | 47.19 |
|  | 15 | 86.57 | 84.05 | 59.32 | 114 | 80.74 | 52.14 | 46.81 |
|  | 16 | 86.49 | 83.61 | 59.06 | 115 | 80.68 | 52.75 | 47.17 |
|  | 17 | 86.41 | 83.1 | 59.06 | 116 | 80.63 | 53.41 | 47.54 |
|  | 18 | 86.35 | 82.86 | 59.04 | 117 | 80.57 | 53.93 | 47.57 |
|  | 19 | 86.32 | 82.61 | 59.18 | 118 | 80.27 | 54.53 | 47.57 |
|  | 20 | 86.27 | 82.61 | 59.18 | 119 | 80.22 | 54.53 | 48.17 |
|  | 21 | 86.23 | 82.21 | 58.92 | 120 | 79.79 | 54.87 | 48.74 |
|  | 22 | 86.2 | 81.97 | 59.19 | 121 | 79.48 | 55.45 | 47.84 |
|  | 23 | 86.17 | 81.66 | 58.62 | 122 | 79.44 | 55.87 | 48.4 |
|  | 24 | 86.17 | 81.36 | 58.74 | 123 | 79.16 | 56.23 | 48.84 |
|  | 25 | 86.14 | 81.14 | 58.74 | 124 | 79.1 | 56.57 | 48.84 |
|  | 26 | 86.14 | 80.79 | 58.35 | 125 | 78.82 | 57.04 | 48.56 |
|  | 27 | 86.1 | 80.67 | 58.11 | 126 | 78.46 | 57.22 | 48.56 |
|  | 28 | 86.08 | 80.47 | 58.24 | 127 | 77.95 | 57.59 | 48.39 |
|  | 29 | 86.07 | 80.47 | 58.72 | 128 | 77.2 | 57.59 | 47.62 |
|  | 30 | 86.03 | 80.43 | 58.75 | 129 | 76.82 | 57.75 | 46.95 |
|  | 31 | 86.03 | 74.92 | 58.76 | 130 | 75.78 | 58.05 | 47.05 |
|  | 32 | 86.03 | 69.6 | 58.95 | 131 | 74.96 | 58.15 | 48.42 |
|  | 33 | 86.01 | 64.27 | 58.95 | 132 | 74.63 | 58.34 | 47.57 |
|  | 34 | 85.97 | 64.36 | 58.81 | 133 | 74.57 | 58.51 | 48.26 |
|  | 35 | 85.98 | 66.12 | 58.82 | 134 | 74.31 | 58.86 | 48.06 |
|  | 36 | 85.96 | 64.94 | 58.61 | 135 | 74.27 | 59.03 | 48.06 |
|  | 37 | 85.94 | 64.94 | 58.37 | 136 | 74.22 | 59.11 | 48.73 |
|  | 38 | 85.91 | 63.14 | 58.4 | 137 | 74.21 | 59.11 | 48.58 |
|  | 39 | 85.89 | 61.81 | 58.4 | 138 | 74.24 | 59.32 | 49.17 |
|  | 40 | 85.85 | 60.09 | 58.73 | 139 | 74.28 | 59.72 | 48.6 |
|  | 41 | 85.82 | 58.17 | 58.73 | 140 | 74.34 | 60.01 | 49.17 |
|  | 42 | 85.79 | 54.78 | 58.55 | 141 | 74.56 | 60.14 | 48.88 |
|  | 43 | 85.76 | 52.91 | 58.77 | 142 | 74.56 | 60.43 | 49.59 |
|  | 44 | 85.72 | 51.51 | 58.77 | 143 | 74.55 | 60.51 | 49.59 |
|  | 45 | 85.67 | 49.65 | 58.78 | 144 | 74.58 | 60.83 | 49.32 |
|  | 46 | 85.62 | 47.66 | 58.89 | 145 | 74.63 | 60.92 | 49.66 |
|  | 47 | 85.58 | 47.66 | 58.65 | 146 | 74.62 | 60.92 | 49.54 |
|  | 48 | 85.55 | 45.81 | 59.61 | 147 | 74.55 | 61.18 | 49.85 |
|  | 49 | 85.49 | 44.51 | 59.61 | 148 | 74.26 | 61.15 | 49.73 |
|  | 50 | 85.42 | 43.06 | 60 | 149 | 74.29 | 61.45 | 50.02 |
|  | 51 | 85.37 | 41.93 | 60.53 | 150 | 74.27 | 61.47 | 49.79 |
|  | 52 | 85.3 | 40.97 | 60.52 | 151 | 74.23 | 61.59 | 50.04 |
|  | 53 | 85.22 | 40.51 | 60.48 | 152 | 74.2 | 61.71 | 50.04 |
|  | 54 | 85.15 | 40.04 | 59.29 | 153 | 74.15 | 61.88 | 49.74 |
|  | 55 | 85.1 | 39.64 | 58.09 | 154 | 73.98 | 61.92 | 49.97 |
|  | 56 | 85.01 | 39.64 | 56.87 | 155 | 73.95 | 61.92 | 49.5 |
|  | 57 | 84.93 | 39.28 | 56.87 | 156 | 73.91 | 62.08 | 49.14 |
|  | 58 | 84.86 | 38.93 | 54.97 | 157 | 73.9 | 62.22 | 48.73 |
|  | 59 | 84.8 | 38.65 | 54.32 | 158 | 73.9 | 62.25 | 49.32 |
|  | 60 | 84.74 | 38.3 | 53.24 | 159 | 73.89 | 62.19 | 49.57 |
|  | 61 | 84.67 | 37.93 | 53.22 | 160 | 73.9 | 62.11 | 49.57 |
|  | 62 | 84.62 | 37.73 | 52.38 | 161 | 73.91 | 62.25 | 49.57 |
|  | 63 | 84.58 | 37.27 | 51.74 | 162 | 73.89 | 62.25 | 49.55 |
|  | 64 | 84.57 | 36.97 | 50.78 | 163 | 73.87 | 62.29 | 49.35 |
|  | 65 | 84.55 | 36.97 | 50.78 | 164 | 73.82 | 62.29 | 48.86 |
|  | 66 | 84.51 | 36.43 | 50.36 | 165 | 73.78 | 62.29 | 48.86 |
|  | 67 | 84.48 | 36.15 | 50.35 | 166 | 73.76 | 62.3 | 49.34 |
|  | 68 | 84.48 | 35.79 | 49.79 | 167 | 73.79 | 62.23 | 49.56 |
|  | 69 | 84.46 | 35.35 | 47.53 | 168 | 73.83 | 62.38 | 49.56 |
|  | 70 | 84.46 | 35.03 | 47.39 | 169 | 73.85 | 62.3 | 49.7 |
|  | 71 | 84.42 | 34.57 | 47.59 | 170 | 73.85 | 62.53 | 49.72 |
|  | 72 | 84.39 | 34.25 | 47.26 | 171 | 73.88 | 62.54 | 49.96 |
|  | 73 | 84.41 | 33.9 | 48.27 | 172 | 73.92 | 62.53 | 49.75 |
|  | 74 | 84.36 | 33.9 | 48.27 | 173 | 74.02 | 62.53 | 50.12 |
|  | 75 | 84.34 | 33.34 | 46.72 | 174 | 74.28 | 62.64 | 49.95 |
|  | 76 | 84.33 | 31.41 | 48.06 | 175 | 74.27 | 62.62 | 50.14 |
|  | 77 | 84.33 | 31.65 | 45.7 | 176 | 74.31 | 62.72 | 50.04 |
|  | 78 | 84.29 | 31.79 | 47.48 | 177 | 74.33 | 62.88 | 50.04 |
|  | 79 | 84.28 | 31.82 | 46.44 | 178 | 74.38 | 62.71 | 50.14 |
|  | 80 | 84.27 | 31.55 | 46.82 | 179 | 74.44 | 62.85 | 50.17 |
|  | 81 | 84.28 | 31.46 | 45.78 | 180 | 74.51 | 62.86 | 50.45 |
|  | 82 | 84.25 | 31.49 | 47.87 | 181 | 74.56 | 62.86 | 50.41 |
|  | 83 | 84.26 | 31.49 | 47.87 | 182 | 74.57 | 62.87 | 50.32 |
|  | 84 | 84.29 | 31.64 | 47.51 | 183 | 74.58 | 62.86 | 51.08 |
|  | 85 | 84.27 | 31.79 | 46.75 | 184 | 74.62 | 62.91 | 50.46 |
|  | 86 | 84.25 | 32.04 | 47.42 | 185 | 74.65 | 63.19 | 50.46 |
|  | 87 | 84.21 | 32.53 | 48.11 | 186 | 74.72 | 63.32 | 50.49 |
|  | 88 | 84.2 | 32.9 | 46.75 | 187 | 74.79 | 63.48 | 50.63 |
|  | 89 | 84.21 | 33.44 | 46.73 | 188 | 74.86 | 63.66 | 50.51 |
|  | 90 | 84.2 | 34.14 | 46.93 | 189 | 75.06 | 63.93 | 50.85 |
|  | 91 | 84.19 | 34.91 | 46.93 | 190 | 75.09 | 63.93 | 50.48 |
|  | 92 | 84.15 | 34.91 | 47.99 | 191 | 75.17 | 63.98 | 51.04 |
|  | 93 | 84.12 | 35.69 | 47.52 | 192 | 75.42 | 64.21 | 50.77 |
|  | 94 | 84.08 | 36.58 | 48.24 | 193 | 75.43 | 64.6 | 51.24 |
|  | 95 | 83.81 | 37.7 | 47.53 | 194 | 75.51 | 64.79 | 51.24 |
|  | 96 | 83.38 | 38.76 | 47.86 | 195 | 75.6 | 64.84 | 51.53 |
|  | 97 | 83.04 | 39.87 | 46.66 | 196 | 75.87 | 65.05 | 50.45 |
|  | 98 | 82.64 | 41.07 | 46.69 | 197 | 75.86 | 65.14 | 51.38 |
|  | 99 | 82.29 | 42 | 46.97 | 198 | 75.89 | 65.26 | 51.20 |
| c | 1 | 85.09 | 82.24 | 60.47 | 50 | 83.75 | 49.49 | 61.67 |
|  | 2 | 85.07 | 82.26 | 60.23 | 51 | 83.73 | 48.29 | 61.4 |
|  | 3 | 85.07 | 82.26 | 60.04 | 52 | 83.73 | 47.21 | 61.08 |
|  | 4 | 85.07 | 82.31 | 59.93 | 53 | 83.73 | 46.13 | 61.08 |
|  | 5 | 85.07 | 82.28 | 60.23 | 54 | 83.75 | 45.59 | 61.08 |
|  | 6 | 85.07 | 82.3 | 60.23 | 55 | 83.76 | 45.01 | 60.89 |
|  | 7 | 85.06 | 82.44 | 60.23 | 56 | 83.74 | 44.32 | 60.65 |
|  | 8 | 85.04 | 82.59 | 60.11 | 57 | 83.75 | 44.32 | 60.55 |
|  | 9 | 85.01 | 82.5 | 60.35 | 58 | 83.74 | 43.72 | 57.94 |
|  | 10 | 84.94 | 82.6 | 60.34 | 59 | 83.75 | 43.1 | 57.93 |
|  | 11 | 84.92 | 82.79 | 60.11 | 60 | 83.71 | 42.21 | 57.18 |
|  | 12 | 84.85 | 82.79 | 59.73 | 61 | 83.67 | 41.29 | 57.18 |
|  | 13 | 84.86 | 82.79 | 59.96 | 62 | 83.62 | 40.48 | 53.42 |
|  | 14 | 84.83 | 82.81 | 59.96 | 63 | 83.54 | 39.65 | 52.57 |
|  | 15 | 84.77 | 82.68 | 59.77 | 64 | 83.49 | 38.62 | 49.99 |
|  | 16 | 84.72 | 82.43 | 59.78 | 65 | 83.29 | 37.75 | 49.5 |
|  | 17 | 84.63 | 82.43 | 59.9 | 66 | 83.25 | 37.75 | 51 |
|  | 18 | 84.56 | 82.25 | 60.43 | 67 | 83.04 | 37.16 | 48.3 |
|  | 19 | 84.52 | 82.28 | 60.61 | 68 | 83.06 | 36.4 | 49.59 |
|  | 20 | 84.47 | 82.13 | 60.6 | 69 | 83.07 | 35.79 | 49.98 |
|  | 21 | 84.4 | 82.13 | 61.13 | 70 | 83.06 | 35.52 | 49.98 |
|  | 22 | 84.35 | 82.12 | 61.13 | 71 | 83.06 | 35.19 | 48.01 |
|  | 23 | 84.28 | 81.99 | 61.1 | 72 | 83.04 | 34.95 | 47.49 |
|  | 24 | 84.24 | 81.82 | 60.44 | 73 | 83.02 | 34.9 | 47.17 |
|  | 25 | 84.18 | 81.95 | 60.22 | 74 | 83.03 | 34.93 | 48.89 |
|  | 26 | 84.15 | 81.86 | 60.03 | 75 | 83 | 34.93 | 47.57 |
|  | 27 | 84.11 | 81.75 | 59.74 | 76 | 83.03 | 34.92 | 48.13 |
|  | 28 | 84.11 | 81.75 | 59.44 | 77 | 83.01 | 35.12 | 47.9 |
|  | 29 | 84.08 | 81.87 | 59.56 | 78 | 83.03 | 35.33 | 48.66 |
|  | 30 | 84.09 | 81.87 | 59.56 | 79 | 83.02 | 35.55 | 48.66 |
|  | 31 | 84.06 | 81.77 | 59.55 | 80 | 83.01 | 35.92 | 49 |
|  | 32 | 84.07 | 77.35 | 59.79 | 81 | 82.99 | 36.23 | 49.32 |
|  | 33 | 84.07 | 69.45 | 60.02 | 82 | 83 | 36.76 | 49.92 |
|  | 34 | 84.02 | 64.93 | 60.02 | 83 | 82.99 | 37.35 | 50.59 |
|  | 35 | 83.99 | 63.9 | 60.35 | 84 | 82.97 | 37.35 | 50.72 |
|  | 36 | 83.95 | 62.77 | 60.47 | 85 | 82.97 | 37.72 | 49.8 |
|  | 37 | 83.93 | 61.98 | 60.47 | 86 | 82.95 | 38.29 | 50.85 |
|  | 38 | 83.89 | 59.54 | 60.35 | 87 | 82.94 | 38.9 | 50.85 |
|  | 39 | 83.86 | 59.54 | 60.34 | 88 | 82.97 | 39.39 | 50.16 |
|  | 40 | 83.81 | 56.76 | 60.41 | 89 | 82.95 | 40.14 | 50.63 |
|  | 41 | 83.79 | 55.51 | 60.43 | 90 | 82.96 | 40.69 | 51.11 |
|  | 42 | 83.78 | 54.75 | 60.44 | 91 | 82.93 | 41.51 | 50.38 |
|  | 43 | 83.76 | 53.78 | 60.74 | 92 | 82.94 | 42.24 | 51.4 |
|  | 44 | 83.74 | 52.53 | 61.06 | 93 | 82.93 | 42.24 | 51.39 |
|  | 45 | 83.75 | 52 | 61.06 | 94 | 82.92 | 43.09 | 51.06 |
|  | 46 | 83.75 | 51.36 | 61.8 | 95 | 82.94 | 44.05 | 51.81 |
|  | 47 | 83.74 | 50.95 | 62.12 | 96 | 82.93 | 45.05 | 51.81 |
|  | 48 | 83.74 | 50.95 | 62.12 | 97 | 82.91 | 45.97 | 51.99 |
|  | 49 | 83.72 | 50.28 | 61.9 | 98 | 82.9 | 46.89 | 52.08 |
| d | 1 | 87.55 | 86.75 | 62.73 | 150 | 75.58 | 65.74 | 52.46 |
|  | 2 | 87.54 | 87.22 | 63.02 | 151 | 75.21 | 66 | 52.71 |
|  | 3 | 87.56 | 87.43 | 63.02 | 152 | 75.18 | 66.22 | 53.2 |
|  | 4 | 87.65 | 87.55 | 62.88 | 153 | 74.93 | 66.35 | 53.69 |
|  | 5 | 87.68 | 87.9 | 63.22 | 154 | 74.88 | 66.65 | 53.57 |
|  | 6 | 87.76 | 88.17 | 63.2 | 155 | 74.9 | 66.68 | 53.56 |
|  | 7 | 87.88 | 88.35 | 62.69 | 156 | 74.83 | 66.8 | 53.52 |
|  | 8 | 87.91 | 88.35 | 62.69 | 157 | 74.8 | 66.8 | 53.52 |
|  | 9 | 87.93 | 88.71 | 62.47 | 158 | 74.75 | 67.12 | 53.71 |
|  | 10 | 87.91 | 88.92 | 62.58 | 159 | 74.77 | 67.15 | 53.85 |
|  | 11 | 87.92 | 89.15 | 62.58 | 160 | 74.82 | 67.57 | 53.97 |
|  | 12 | 87.93 | 89.34 | 62.59 | 161 | 74.83 | 67.73 | 53.97 |
|  | 13 | 87.92 | 89.6 | 63.01 | 162 | 74.86 | 67.82 | 54.33 |
|  | 14 | 87.86 | 89.79 | 62.88 | 163 | 74.84 | 68.17 | 54.5 |
|  | 15 | 87.8 | 89.95 | 63.02 | 164 | 74.82 | 68.38 | 54.5 |
|  | 16 | 87.72 | 89.96 | 62.85 | 165 | 74.82 | 68.72 | 54.62 |
|  | 17 | 87.65 | 89.96 | 62.86 | 166 | 74.82 | 68.72 | 54.62 |
|  | 18 | 87.52 | 90 | 62.87 | 167 | 74.79 | 69.05 | 54.78 |
|  | 19 | 87.15 | 88.54 | 62.87 | 168 | 74.8 | 69.15 | 54.77 |
|  | 20 | 87.1 | 86.44 | 62.92 | 169 | 74.77 | 69.18 | 54.5 |
|  | 21 | 87.02 | 85.39 | 62.7 | 170 | 74.76 | 69.3 | 54.14 |
|  | 22 | 86.78 | 85.42 | 62.69 | 171 | 74.82 | 69.18 | 53.43 |
|  | 23 | 86.8 | 85.37 | 62.31 | 172 | 74.92 | 69.11 | 52.99 |
|  | 24 | 86.78 | 82.78 | 62.03 | 173 | 74.93 | 68.75 | 52.34 |
|  | 25 | 86.76 | 82.78 | 62.21 | 174 | 75.21 | 68.12 | 52.34 |
|  | 26 | 86.76 | 76.8 | 62.06 | 175 | 75.24 | 68.12 | 51.88 |
|  | 27 | 86.84 | 71.32 | 62.06 | 176 | 75.3 | 67.32 | 51.27 |
|  | 28 | 86.88 | 68.74 | 61.05 | 177 | 75.33 | 66.55 | 50.73 |
|  | 29 | 86.94 | 68.16 | 61.05 | 178 | 75.41 | 65.69 | 50.43 |
|  | 30 | 86.96 | 67.42 | 60.76 | 179 | 75.49 | 64.96 | 50.68 |
|  | 31 | 86.96 | 65.91 | 60.75 | 180 | 75.73 | 64.22 | 50.69 |
|  | 32 | 86.95 | 63.72 | 60.47 | 181 | 75.77 | 63.37 | 51 |
|  | 33 | 86.85 | 61.81 | 60.13 | 182 | 76 | 62.35 | 51 |
|  | 34 | 86.79 | 61.81 | 59.24 | 183 | 76.01 | 61.71 | 51.25 |
|  | 35 | 86.72 | 60.61 | 59.24 | 184 | 76.28 | 61.71 | 51.77 |
|  | 36 | 86.62 | 59.67 | 58.71 | 185 | 76.36 | 61.06 | 51.96 |
|  | 37 | 86.59 | 58.1 | 57.83 | 186 | 76.64 | 60.31 | 51.8 |
|  | 38 | 86.51 | 57.34 | 57.07 | 187 | 76.94 | 59.66 | 52 |
|  | 39 | 86.47 | 57.23 | 56.64 | 188 | 77.02 | 58.99 | 52.15 |
|  | 40 | 86.45 | 57.52 | 56.15 | 189 | 77.32 | 58.44 | 52.3 |
|  | 41 | 86.46 | 57.88 | 55.22 | 190 | 77.39 | 58.05 | 52.3 |
|  | 42 | 86.39 | 57.95 | 54.99 | 191 | 77.63 | 57.67 | 52.44 |
|  | 43 | 86.36 | 57.95 | 54.99 | 192 | 77.72 | 57.47 | 52.43 |
|  | 44 | 86.25 | 58.08 | 55 | 193 | 77.92 | 57.47 | 52.46 |
|  | 45 | 86.21 | 58.35 | 53.93 | 194 | 78.03 | 57.16 | 52.46 |
|  | 46 | 86.14 | 58.61 | 54.7 | 195 | 78.31 | 56.93 | 52.65 |
|  | 47 | 86.08 | 58.88 | 53.41 | 196 | 78.38 | 56.79 | 52.78 |
|  | 48 | 85.99 | 59.15 | 53.19 | 197 | 78.71 | 56.83 | 52.58 |
|  | 49 | 85.94 | 59.43 | 53.06 | 198 | 78.8 | 56.63 | 52.58 |
|  | 50 | 85.92 | 59.74 | 52.34 | 199 | 79.12 | 56.58 | 52.25 |
|  | 51 | 85.91 | 59.99 | 51.99 | 200 | 79.2 | 56.69 | 51.48 |
|  | 52 | 85.93 | 59.99 | 51.99 | 201 | 79.48 | 56.69 | 51.46 |
|  | 53 | 85.94 | 60.11 | 51.99 | 202 | 79.79 | 56.65 | 51.69 |
|  | 54 | 85.95 | 60.21 | 51.46 | 203 | 79.86 | 56.74 | 51.81 |
|  | 55 | 85.97 | 60.23 | 51.91 | 204 | 80.18 | 56.82 | 51.96 |
|  | 56 | 85.98 | 60.39 | 51.65 | 205 | 80.22 | 56.96 | 51.94 |
|  | 57 | 85.94 | 60.27 | 52.17 | 206 | 80.49 | 57.17 | 51.82 |
|  | 58 | 85.95 | 60.34 | 52.79 | 207 | 80.76 | 57.31 | 51.82 |
|  | 59 | 85.88 | 60.28 | 53.15 | 208 | 80.8 | 57.43 | 51.84 |
|  | 60 | 85.88 | 60.23 | 53.56 | 209 | 81.15 | 57.71 | 52.11 |
|  | 61 | 85.84 | 60.23 | 53.56 | 210 | 81.19 | 57.71 | 52.23 |
|  | 62 | 85.88 | 60.37 | 53.39 | 211 | 81.53 | 57.84 | 52.24 |
|  | 63 | 85.82 | 60.17 | 53.4 | 212 | 81.89 | 58.01 | 52.28 |
|  | 64 | 85.83 | 60.15 | 53.36 | 213 | 81.99 | 57.99 | 52.41 |
|  | 65 | 85.8 | 59.68 | 54.11 | 214 | 82.28 | 58.18 | 52.35 |
|  | 66 | 85.78 | 59.56 | 53.92 | 215 | 82.34 | 58.3 | 52.35 |
|  | 67 | 85.74 | 59.43 | 54.35 | 216 | 82.63 | 58.42 | 52.5 |
|  | 68 | 85.7 | 59.12 | 54.12 | 217 | 82.9 | 58.79 | 52.52 |
|  | 69 | 85.65 | 58.92 | 54.36 | 218 | 82.95 | 58.95 | 52.54 |
|  | 70 | 85.62 | 58.92 | 54.36 | 219 | 83.21 | 58.95 | 52.71 |
|  | 71 | 85.61 | 58.45 | 54.5 | 220 | 83.27 | 59.11 | 52.73 |
|  | 72 | 85.62 | 58.34 | 54.49 | 221 | 83.6 | 58.95 | 52.7 |
|  | 73 | 85.6 | 58.16 | 54.91 | 222 | 83.62 | 59.22 | 52.51 |
|  | 74 | 85.56 | 57.86 | 54.91 | 223 | 83.71 | 59.22 | 52.51 |
|  | 75 | 85.57 | 57.65 | 54.51 | 224 | 83.99 | 59.1 | 51.58 |
|  | 76 | 85.56 | 57.49 | 54.75 | 225 | 84.03 | 59.14 | 51.08 |
|  | 77 | 85.56 | 57.61 | 55.04 | 226 | 84.1 | 59.28 | 51.34 |
|  | 78 | 85.54 | 57.63 | 55.07 | 227 | 84.31 | 59.45 | 51.98 |
|  | 79 | 85.55 | 57.63 | 55.07 | 228 | 84.4 | 59.45 | 51.82 |
|  | 80 | 85.51 | 57.68 | 55.27 | 229 | 84.45 | 59.59 | 51.82 |
|  | 81 | 85.47 | 57.96 | 55.47 | 230 | 84.64 | 59.48 | 52.1 |
|  | 82 | 85.52 | 58.32 | 55.78 | 231 | 84.76 | 59.7 | 52.11 |
|  | 83 | 85.51 | 58.9 | 55.91 | 232 | 85 | 59.81 | 52.11 |
|  | 84 | 85.49 | 59.52 | 56.17 | 233 | 85.06 | 59.86 | 52.14 |
|  | 85 | 85.54 | 60.09 | 55.63 | 234 | 85.32 | 59.91 | 52.25 |
|  | 86 | 85.55 | 60.78 | 53.89 | 235 | 85.38 | 59.9 | 52.23 |
|  | 87 | 85.54 | 60.78 | 53.08 | 236 | 85.65 | 59.9 | 51.99 |
|  | 88 | 85.56 | 61.53 | 53.08 | 237 | 85.72 | 60.05 | 51.25 |
|  | 89 | 85.57 | 62.33 | 53.05 | 238 | 85.96 | 60.18 | 50.89 |
|  | 90 | 85.6 | 63.15 | 54.14 | 239 | 86.24 | 60.29 | 50.74 |
|  | 91 | 85.62 | 63.2 | 53.79 | 240 | 86.25 | 60.19 | 50.74 |
|  | 92 | 85.6 | 63.29 | 53.67 | 241 | 86.59 | 60.4 | 50.75 |
|  | 93 | 85.59 | 64.06 | 53.84 | 242 | 86.58 | 60.62 | 51.17 |
|  | 94 | 85.55 | 65 | 54.22 | 243 | 86.61 | 60.57 | 51.17 |
|  | 95 | 85.37 | 65.63 | 54.55 | 244 | 86.63 | 60.82 | 51.08 |
|  | 96 | 85.22 | 65.63 | 54.35 | 245 | 86.89 | 60.82 | 50.72 |
|  | 97 | 84.96 | 65.85 | 54.35 | 246 | 86.89 | 60.98 | 50.55 |
|  | 98 | 84.68 | 65.89 | 54.49 | 247 | 86.94 | 60.85 | 50.55 |
|  | 99 | 84.66 | 65.89 | 54.51 | 248 | 87.01 | 61.1 | 50.55 |
|  | 100 | 84.31 | 66.06 | 55.03 | 249 | 87.01 | 61.11 | 50.12 |
|  | 101 | 83.93 | 66.29 | 55.36 | 250 | 87 | 60.98 | 50.15 |
|  | 102 | 83.53 | 66.45 | 54.58 | 251 | 87.03 | 61.13 | 49.89 |
|  | 103 | 83.51 | 66.7 | 54.16 | 252 | 87.27 | 61.09 | 49.9 |
|  | 104 | 83.13 | 66.84 | 54.03 | 253 | 87.26 | 61.21 | 49.9 |
|  | 105 | 82.83 | 66.84 | 54.03 | 254 | 87.28 | 61.21 | 49.84 |
|  | 106 | 82.48 | 67.11 | 53.69 | 255 | 87.32 | 61.2 | 49.84 |
|  | 107 | 82.02 | 67.2 | 52.9 | 256 | 87.37 | 61.29 | 49.84 |
|  | 108 | 81.99 | 67.48 | 52.89 | 257 | 87.65 | 61.36 | 49.84 |
|  | 109 | 81.61 | 67.62 | 52.71 | 258 | 87.65 | 61.61 | 50.34 |
|  | 110 | 81.19 | 67.54 | 53 | 259 | 87.74 | 61.79 | 50.62 |
|  | 111 | 81.13 | 67.84 | 53.45 | 260 | 87.8 | 62.04 | 51.12 |
|  | 112 | 80.83 | 67.93 | 53.15 | 261 | 87.84 | 62.34 | 51.57 |
|  | 113 | 80.76 | 68.33 | 53.41 | 262 | 87.88 | 62.46 | 51.58 |
|  | 114 | 80.52 | 68.33 | 53.41 | 263 | 88.2 | 62.46 | 51.92 |
|  | 115 | 80.45 | 68.55 | 53.81 | 264 | 88.2 | 62.96 | 51.92 |
|  | 116 | 80.17 | 68.67 | 54.05 | 265 | 88.25 | 63.13 | 51.92 |
|  | 117 | 80.14 | 68.79 | 54.13 | 266 | 88.29 | 63.63 | 51.92 |
|  | 118 | 79.74 | 68.97 | 54.37 | 267 | 88.39 | 64.06 | 52.21 |
|  | 119 | 79.67 | 69.14 | 54.38 | 268 | 88.36 | 64.43 | 52.21 |
|  | 120 | 79.42 | 69.03 | 54.41 | 269 | 88.31 | 64.85 | 52.08 |
|  | 121 | 79.39 | 69.08 | 54.93 | 270 | 88.2 | 65.29 | 51.93 |
|  | 122 | 79.35 | 69.08 | 55.06 | 271 | 88.21 | 65.61 | 51.8 |
|  | 123 | 79.3 | 69.03 | 55.06 | 272 | 88.21 | 65.61 | 51.79 |
|  | 124 | 79.23 | 68.71 | 55.33 | 273 | 88.28 | 66.09 | 51.61 |
|  | 125 | 79.12 | 68.46 | 55.58 | 274 | 88.55 | 66.6 | 51.61 |
|  | 126 | 79.1 | 67.99 | 55.61 | 275 | 88.63 | 66.99 | 51.61 |
|  | 127 | 79.01 | 67.5 | 55.78 | 276 | 88.66 | 67.32 | 51.25 |
|  | 128 | 78.99 | 66.85 | 55.86 | 277 | 88.68 | 67.91 | 51.06 |
|  | 129 | 78.99 | 66.57 | 55.8 | 278 | 88.68 | 68.27 | 51.24 |
|  | 130 | 78.95 | 65.93 | 55.51 | 279 | 88.74 | 68.57 | 51.37 |
|  | 131 | 78.91 | 65.93 | 55.51 | 280 | 88.76 | 68.57 | 51.48 |
|  | 132 | 78.92 | 65.52 | 55.54 | 281 | 88.82 | 69.13 | 51.98 |
|  | 133 | 78.59 | 64.99 | 55.52 | 282 | 88.86 | 69.41 | 51.98 |
|  | 134 | 78.25 | 64.61 | 55.5 | 283 | 88.91 | 69.68 | 51.75 |
|  | 135 | 77.86 | 64.4 | 55.37 | 284 | 88.92 | 69.73 | 51.52 |
|  | 136 | 77.55 | 64.21 | 55.36 | 285 | 88.95 | 69.97 | 51.5 |
|  | 137 | 77.25 | 63.95 | 55.2 | 286 | 88.96 | 70.1 | 51.49 |
|  | 138 | 76.91 | 63.86 | 54.58 | 287 | 89.01 | 70.21 | 51.47 |
|  | 139 | 76.86 | 63.83 | 53.27 | 288 | 89.03 | 70.35 | 52.07 |
|  | 140 | 76.56 | 63.83 | 53.27 | 289 | 89.08 | 70.35 | 52.32 |
|  | 141 | 76.53 | 63.83 | 52.84 | 290 | 89.11 | 70.51 | 52.31 |
|  | 142 | 76.5 | 63.97 | 53.35 | 291 | 89.12 | 70.52 | 52.31 |
|  | 143 | 76.44 | 64.06 | 53.66 | 292 | 89.06 | 70.76 | 52.05 |
|  | 144 | 76.27 | 64.4 | 53.51 | 293 | 89.05 | 70.76 | 52.59 |
|  | 145 | 76.24 | 64.61 | 52.96 | 294 | 88.97 | 70.93 | 52.84 |
|  | 146 | 76.13 | 64.93 | 52.74 | 295 | 88.93 | 71.01 | 52.87 |
|  | 147 | 75.92 | 65.13 | 52.61 | 296 | 88.9 | 71.11 | 52.84 |
|  | 148 | 75.9 | 65.55 | 52.47 | 297 | 88.94 | 71.22 | 52.59 |
|  | 149 | 75.6 | 65.55 | 52.47 | 298 | 89.06 | 71.28 | 52.82 |
| e | 1 | 86.81 | 79.73 | 56.13 | 50 | 84.95 | 47.09 | 48.71 |
|  | 2 | 86.74 | 79.73 | 56.19 | 51 | 84.94 | 46.36 | 47.56 |
|  | 3 | 86.67 | 79.75 | 56.51 | 52 | 84.9 | 45.56 | 46.88 |
|  | 4 | 86.58 | 79.76 | 56.73 | 53 | 84.85 | 44.62 | 46.43 |
|  | 5 | 86.31 | 79.96 | 56.33 | 54 | 84.84 | 43.65 | 49.02 |
|  | 6 | 86.32 | 80.01 | 56.1 | 55 | 84.8 | 42.49 | 47.6 |
|  | 7 | 86.29 | 80.32 | 56.37 | 56 | 84.75 | 42.49 | 46.87 |
|  | 8 | 86 | 80.47 | 56.37 | 57 | 84.7 | 41.59 | 46.87 |
|  | 9 | 85.95 | 80.65 | 56.56 | 58 | 84.64 | 40.65 | 46.35 |
|  | 10 | 85.89 | 80.77 | 56.13 | 59 | 84.63 | 39.5 | 46.18 |
|  | 11 | 85.78 | 80.77 | 56.14 | 60 | 84.59 | 38.72 | 45.62 |
|  | 12 | 85.69 | 80.93 | 56.47 | 61 | 84.53 | 37.76 | 45.86 |
|  | 13 | 85.66 | 81.04 | 56.89 | 62 | 84.49 | 37 | 45.19 |
|  | 14 | 85.65 | 81.13 | 56.92 | 63 | 84.48 | 36.24 | 45.16 |
|  | 15 | 85.6 | 81.17 | 56.71 | 64 | 84.46 | 35.69 | 46.03 |
|  | 16 | 85.61 | 81.5 | 56.71 | 65 | 84.49 | 35.69 | 46.51 |
|  | 17 | 85.62 | 81.56 | 57.04 | 66 | 84.49 | 35.05 | 46.51 |
|  | 18 | 85.6 | 81.71 | 57.46 | 67 | 84.45 | 34.48 | 45.57 |
|  | 19 | 85.64 | 81.79 | 57.1 | 68 | 84.46 | 34.33 | 46.01 |
|  | 20 | 85.58 | 81.79 | 56.8 | 69 | 84.44 | 34.29 | 44.89 |
|  | 21 | 85.58 | 81.93 | 56.57 | 70 | 84.41 | 34.07 | 47.7 |
|  | 22 | 85.46 | 81.97 | 56.57 | 71 | 84.39 | 34.09 | 46.79 |
|  | 23 | 85.41 | 65.33 | 56.58 | 72 | 84.36 | 34.11 | 46.33 |
|  | 24 | 85.38 | 66.54 | 56.58 | 73 | 84.3 | 34.45 | 46.68 |
|  | 25 | 85.35 | 68.66 | 56.43 | 74 | 84.28 | 34.45 | 46.55 |
|  | 26 | 85.33 | 71.18 | 56.79 | 75 | 84.25 | 34.67 | 46.55 |
|  | 27 | 85.28 | 72.37 | 56.97 | 76 | 84.22 | 35.01 | 47.04 |
|  | 28 | 85.27 | 68.32 | 58.08 | 77 | 84.21 | 35.44 | 46.45 |
|  | 29 | 85.26 | 68.32 | 59.12 | 78 | 84.15 | 35.85 | 47.22 |
|  | 30 | 85.26 | 65.52 | 59.54 | 79 | 84.1 | 36.38 | 47.09 |
|  | 31 | 85.23 | 64.05 | 59.1 | 80 | 84.04 | 36.8 | 47.49 |
|  | 32 | 85.24 | 63.45 | 59.1 | 81 | 83.99 | 37.46 | 46.61 |
|  | 33 | 85.22 | 63.21 | 59.11 | 82 | 83.95 | 38.2 | 47.51 |
|  | 34 | 85.24 | 62.26 | 59.06 | 83 | 83.91 | 38.2 | 47.51 |
|  | 35 | 85.28 | 60.69 | 58.52 | 84 | 83.85 | 38.86 | 47.51 |
|  | 36 | 85.27 | 59.17 | 58.73 | 85 | 83.65 | 39.86 | 47.45 |
|  | 37 | 85.26 | 57.79 | 58.42 | 86 | 83.59 | 40.69 | 47.5 |
|  | 38 | 85.27 | 57.79 | 58.41 | 87 | 83.52 | 41.53 | 47.96 |
|  | 39 | 85.27 | 56.85 | 55.7 | 88 | 83.34 | 42.56 | 47.45 |
|  | 40 | 85.26 | 55.78 | 55.7 | 89 | 83.29 | 43.38 | 48.96 |
|  | 41 | 85.25 | 54.67 | 54.34 | 90 | 83.19 | 44.26 | 48.45 |
|  | 42 | 85.24 | 53.44 | 52.94 | 91 | 83.13 | 44.26 | 49.14 |
|  | 43 | 85.18 | 52.2 | 51.62 | 92 | 83.08 | 45.25 | 49.14 |
|  | 44 | 85.14 | 51.2 | 51.27 | 93 | 83.01 | 46.11 | 48.65 |
|  | 45 | 85.09 | 50.2 | 50.69 | 94 | 82.97 | 47.17 | 49.14 |
|  | 46 | 85.07 | 49.37 | 49.25 | 95 | 82.94 | 48.13 | 49.17 |
|  | 47 | 85.04 | 49.37 | 48.34 | 96 | 82.85 | 49.18 | 48.86 |
|  | 48 | 85.03 | 48.54 | 48.34 | 97 | 82.84 | 50.11 | 49.69 |
|  | 49 | 85.01 | 47.8 | 47.17 | 98 | 82.79 | 51.13 | 49.38 |
| f | 1 | 87.33 | 86.25 | 60.88 | 50 | 85.34 | 42.38 | 47.33 |
|  | 2 | 87.36 | 86.58 | 60.56 | 51 | 85.34 | 42.2 | 46.88 |
|  | 3 | 87.38 | 86.58 | 60.56 | 52 | 85.32 | 41.78 | 45.82 |
|  | 4 | 87.36 | 86.56 | 60.56 | 53 | 85.31 | 41.49 | 44.09 |
|  | 5 | 87.37 | 86.78 | 60.56 | 54 | 85.3 | 41.04 | 44.66 |
|  | 6 | 87.33 | 86.76 | 60.73 | 55 | 85.32 | 40.48 | 45.98 |
|  | 7 | 87.28 | 86.83 | 60.39 | 56 | 85.28 | 40.48 | 45.98 |
|  | 8 | 87.24 | 87 | 59.83 | 57 | 85.26 | 39.84 | 45.97 |
|  | 9 | 87.24 | 86.92 | 59.81 | 58 | 85.31 | 39.07 | 46.52 |
|  | 10 | 87.33 | 87.1 | 60.44 | 59 | 85.3 | 38.33 | 45.97 |
|  | 11 | 87.53 | 87.1 | 60.91 | 60 | 85.28 | 37.31 | 46.51 |
|  | 12 | 87.55 | 86.98 | 60.67 | 61 | 85.32 | 36.57 | 46.67 |
|  | 13 | 87.59 | 87.05 | 60.67 | 62 | 85.3 | 35.84 | 46.82 |
|  | 14 | 87.61 | 86.84 | 60.27 | 63 | 85.33 | 35.34 | 47.42 |
|  | 15 | 87.65 | 86.73 | 59.58 | 64 | 85.32 | 34.7 | 47.42 |
|  | 16 | 87.6 | 86.43 | 59.1 | 65 | 85.35 | 34.7 | 47.6 |
|  | 17 | 87.59 | 85.97 | 59.34 | 66 | 85.34 | 34.23 | 47.59 |
|  | 18 | 87.56 | 85.66 | 59.61 | 67 | 85.37 | 33.76 | 46.71 |
|  | 19 | 87.51 | 84.89 | 59.42 | 68 | 85.34 | 33.46 | 48.39 |
|  | 20 | 87.43 | 84.89 | 59.56 | 69 | 85.31 | 33.36 | 48.18 |
|  | 21 | 87.14 | 84.25 | 59.86 | 70 | 85.25 | 33.28 | 48.05 |
|  | 22 | 87.08 | 83.56 | 59.86 | 71 | 85.19 | 33.15 | 48.59 |
|  | 23 | 86.79 | 82.9 | 59.64 | 72 | 85.14 | 33.27 | 48.4 |
|  | 24 | 86.7 | 78.58 | 59.79 | 73 | 85.09 | 33.26 | 48.4 |
|  | 25 | 86.45 | 69.81 | 59.42 | 74 | 85.08 | 33.26 | 48 |
|  | 26 | 86.39 | 62.81 | 59.12 | 75 | 85.05 | 33.14 | 48.86 |
|  | 27 | 86.31 | 61.09 | 59.12 | 76 | 85.04 | 33.65 | 48.95 |
|  | 28 | 86.13 | 59.5 | 59.11 | 77 | 85.01 | 33.92 | 49.08 |
|  | 29 | 86.12 | 59.5 | 59.3 | 78 | 84.94 | 34.28 | 49.36 |
|  | 30 | 86.04 | 58.96 | 59.3 | 79 | 84.9 | 34.76 | 49.36 |
|  | 31 | 85.99 | 57.24 | 58.81 | 80 | 84.88 | 35.27 | 49.54 |
|  | 32 | 85.9 | 55.57 | 58.59 | 81 | 84.88 | 35.66 | 49.95 |
|  | 33 | 85.85 | 54.77 | 58.54 | 82 | 84.85 | 36.46 | 49.95 |
|  | 34 | 85.76 | 53.73 | 58.97 | 83 | 84.83 | 36.46 | 50.19 |
|  | 35 | 85.67 | 52.39 | 58.97 | 84 | 84.82 | 37.12 | 50.44 |
|  | 36 | 85.63 | 50.81 | 58.97 | 85 | 84.77 | 37.83 | 50.56 |
|  | 37 | 85.61 | 49.88 | 59.07 | 86 | 84.74 | 38.62 | 50.69 |
|  | 38 | 85.62 | 49.88 | 59.49 | 87 | 84.64 | 39.53 | 50.94 |
|  | 39 | 85.57 | 48.79 | 59.49 | 88 | 84.62 | 40.36 | 50.74 |
|  | 40 | 85.55 | 48.15 | 59.49 | 89 | 84.59 | 41.19 | 50.75 |
|  | 41 | 85.58 | 47.26 | 58.15 | 90 | 84.56 | 42.28 | 50.75 |
|  | 42 | 85.56 | 46.18 | 56.95 | 91 | 84.51 | 43.28 | 50.75 |
|  | 43 | 85.55 | 45.31 | 55.19 | 92 | 84.48 | 43.28 | 50.9 |
|  | 44 | 85.59 | 44.47 | 53.7 | 93 | 84.4 | 44.48 | 51.04 |
|  | 45 | 85.55 | 43.9 | 51.41 | 94 | 84.12 | 45.63 | 51.05 |
|  | 46 | 85.51 | 43.35 | 50.66 | 95 | 84.06 | 46.81 | 51.07 |
|  | 47 | 85.42 | 43.35 | 50.66 | 96 | 83.65 | 48.03 | 51.08 |
|  | 48 | 85.4 | 43.03 | 48.44 | 97 | 83.31 | 49.32 | 51.13 |
|  | 49 | 85.37 | 42.71 | 46.8 | 98 | 82.99 | 50.59 | 51.29 |
| g | 1 | 83.5 | 81.79 | 49.04 | 150 | 86.18 | 54.45 | 42 |
|  | 2 | 83.49 | 81.55 | 49.31 | 151 | 86.23 | 54.74 | 42 |
|  | 3 | 83.5 | 81.55 | 49.56 | 152 | 86.27 | 54.73 | 42.01 |
|  | 4 | 83.49 | 81.73 | 48.91 | 153 | 86.26 | 54.68 | 41.66 |
|  | 5 | 83.51 | 81.45 | 48.39 | 154 | 86.28 | 54.62 | 41.66 |
|  | 6 | 83.48 | 81.58 | 47.94 | 155 | 86.33 | 54.56 | 41.66 |
|  | 7 | 83.47 | 77.11 | 47.94 | 156 | 86.4 | 54.57 | 41.45 |
|  | 8 | 83.46 | 75.21 | 47.75 | 157 | 86.63 | 54.57 | 41.45 |
|  | 9 | 83.44 | 74.89 | 47.76 | 158 | 86.63 | 54.41 | 41.41 |
|  | 10 | 83.39 | 76.29 | 47.87 | 159 | 86.7 | 54.46 | 41.39 |
|  | 11 | 83.39 | 77.02 | 47.65 | 160 | 86.81 | 54.34 | 41.39 |
|  | 12 | 83.35 | 75.75 | 47.32 | 161 | 86.85 | 54.49 | 41.73 |
|  | 13 | 83.37 | 75.75 | 47.31 | 162 | 86.57 | 54.48 | 41.55 |
|  | 14 | 83.36 | 74.09 | 47.31 | 163 | 86.57 | 54.45 | 41.58 |
|  | 15 | 83.31 | 73.19 | 47.32 | 164 | 86.54 | 54.78 | 41.8 |
|  | 16 | 83.34 | 73.25 | 47.53 | 165 | 86.52 | 54.83 | 41.71 |
|  | 17 | 83.29 | 72.92 | 47.49 | 166 | 86.53 | 54.83 | 41.89 |
|  | 18 | 83.29 | 72.09 | 47.39 | 167 | 86.59 | 55.11 | 41.71 |
|  | 19 | 83.29 | 70.19 | 47.41 | 168 | 86.61 | 55.26 | 41.7 |
|  | 20 | 83.27 | 68.19 | 46.88 | 169 | 86.63 | 55.32 | 41.7 |
|  | 21 | 83.26 | 66.91 | 46.88 | 170 | 86.56 | 55.63 | 42.11 |
|  | 22 | 83.23 | 66.91 | 46.53 | 171 | 86.45 | 55.61 | 42.13 |
|  | 23 | 83.20 | 66.4 | 46.19 | 172 | 86.36 | 55.9 | 41.93 |
|  | 24 | 83.16 | 65.89 | 46.08 | 173 | 86.27 | 56.02 | 42.04 |
|  | 25 | 83.17 | 64.6 | 45.68 | 174 | 86.34 | 56.16 | 42.18 |
|  | 26 | 83.12 | 63.38 | 45.43 | 175 | 86.37 | 56.16 | 42.49 |
|  | 27 | 83.15 | 62.43 | 45.25 | 176 | 86.43 | 56.35 | 42.82 |
|  | 28 | 83.12 | 61.18 | 45.25 | 177 | 86.46 | 56.51 | 43.04 |
|  | 29 | 83.11 | 59.99 | 45.27 | 178 | 86.48 | 56.55 | 43.04 |
|  | 30 | 83.11 | 59.99 | 45.16 | 179 | 86.42 | 56.77 | 43.04 |
|  | 31 | 83.08 | 59.01 | 45.13 | 180 | 86.41 | 57.1 | 43.05 |
|  | 32 | 83.05 | 56.9 | 45.14 | 181 | 86.32 | 57.32 | 42.83 |
|  | 33 | 83.05 | 55.73 | 44.86 | 182 | 86.31 | 57.51 | 42.48 |
|  | 34 | 83.04 | 54.95 | 44.70 | 183 | 86.35 | 57.74 | 42.35 |
|  | 35 | 82.98 | 55.03 | 44.70 | 184 | 86.36 | 57.74 | 42.35 |
|  | 36 | 82.97 | 54.59 | 44.70 | 185 | 86.63 | 58.17 | 42.46 |
|  | 37 | 82.92 | 54.25 | 44.33 | 186 | 86.68 | 58.5 | 42.46 |
|  | 38 | 82.85 | 53.83 | 44.16 | 187 | 86.74 | 58.75 | 42.47 |
|  | 39 | 82.84 | 53.83 | 44.14 | 188 | 86.79 | 59.15 | 42.48 |
|  | 40 | 82.82 | 53.88 | 43.98 | 189 | 86.84 | 59.7 | 42.49 |
|  | 41 | 82.82 | 53.83 | 43.6 | 190 | 86.84 | 60.12 | 42.68 |
|  | 42 | 82.81 | 53.99 | 43.6 | 191 | 86.81 | 60.39 | 42.33 |
|  | 43 | 82.77 | 54.16 | 43.42 | 192 | 86.82 | 60.8 | 42.13 |
|  | 44 | 82.76 | 54.59 | 43.42 | 193 | 86.78 | 60.8 | 41.99 |
|  | 45 | 82.75 | 54.68 | 43.41 | 194 | 86.75 | 61.08 | 41.99 |
|  | 46 | 82.74 | 54.78 | 43.24 | 195 | 86.71 | 61.42 | 41.97 |
|  | 47 | 82.71 | 54.85 | 43.24 | 196 | 86.65 | 61.75 | 41.98 |
|  | 48 | 82.69 | 54.85 | 43.12 | 197 | 86.61 | 61.99 | 42.1 |
|  | 49 | 82.69 | 54.84 | 43.11 | 198 | 86.62 | 62.3 | 42.12 |
|  | 50 | 82.70 | 54.76 | 42.94 | 199 | 86.75 | 62.61 | 42.67 |
|  | 51 | 82.69 | 54.69 | 42.91 | 200 | 86.98 | 63.01 | 43.28 |
|  | 52 | 82.69 | 54.69 | 42.91 | 201 | 86.97 | 63.24 | 43.16 |
|  | 53 | 82.66 | 54.55 | 42.72 | 202 | 87.27 | 63.24 | 43.16 |
|  | 54 | 82.65 | 53.72 | 42.70 | 203 | 87.3 | 63.5 | 43.12 |
|  | 55 | 82.61 | 52.6 | 42.70 | 204 | 87.33 | 64.03 | 42.86 |
|  | 56 | 82.59 | 51.95 | 42.67 | 205 | 87.39 | 64.28 | 42.5 |
|  | 57 | 82.55 | 51.95 | 42.54 | 206 | 87.36 | 64.49 | 42.36 |
|  | 58 | 82.5 | 51.3 | 42.52 | 207 | 87.33 | 64.88 | 42.58 |
|  | 59 | 82.52 | 51.01 | 42.52 | 208 | 87.34 | 65.11 | 42.78 |
|  | 60 | 82.48 | 49.56 | 42.53 | 209 | 87.35 | 65.16 | 42.57 |
|  | 61 | 82.47 | 48.5 | 42.53 | 210 | 87.41 | 65.38 | 42.57 |
|  | 62 | 82.47 | 48.23 | 42.85 | 211 | 87.42 | 65.38 | 42.6 |
|  | 63 | 82.43 | 48.07 | 42.97 | 212 | 87.42 | 65.35 | 42.89 |
|  | 64 | 82.43 | 47.71 | 42.95 | 213 | 87.45 | 65.71 | 43.46 |
|  | 65 | 82.40 | 46.80 | 42.73 | 214 | 87.4 | 65.73 | 43.25 |
|  | 66 | 82.39 | 46.80 | 42.47 | 215 | 87.38 | 65.72 | 43.64 |
|  | 67 | 82.36 | 46.08 | 42.16 | 216 | 87.41 | 65.75 | 43.46 |
|  | 68 | 82.3 | 45.51 | 42.17 | 217 | 87.46 | 65.58 | 43.08 |
|  | 69 | 82.28 | 45.19 | 42.16 | 218 | 87.51 | 65.72 | 43.08 |
|  | 70 | 82.28 | 44.78 | 42.16 | 219 | 87.53 | 65.91 | 42.92 |
|  | 71 | 82.27 | 44.74 | 42.08 | 220 | 87.55 | 65.96 | 42.62 |
|  | 72 | 82.23 | 44.53 | 42.28 | 221 | 87.5 | 65.96 | 42.44 |
|  | 73 | 82.2 | 44.72 | 42.28 | 222 | 87.5 | 66.23 | 42.57 |
|  | 74 | 82.18 | 45.18 | 42.13 | 223 | 87.44 | 66.56 | 42.58 |
|  | 75 | 82.11 | 45.18 | 42.14 | 224 | 87.39 | 66.69 | 42.53 |
|  | 76 | 82.04 | 45.71 | 42.14 | 225 | 87.32 | 67.15 | 42.82 |
|  | 77 | 81.96 | 46.22 | 41.96 | 226 | 87.27 | 67.46 | 42.82 |
|  | 78 | 81.92 | 46.85 | 41.96 | 227 | 87.26 | 67.85 | 42.55 |
|  | 79 | 81.88 | 47.38 | 41.95 | 228 | 87.3 | 68.11 | 42.43 |
|  | 80 | 81.85 | 48.23 | 42.08 | 229 | 87.36 | 68.70 | 42.43 |
|  | 81 | 81.84 | 48.75 | 42.09 | 230 | 87.4 | 68.70 | 42.7 |
|  | 82 | 81.89 | 49.45 | 42.11 | 231 | 87.46 | 69.14 | 42.83 |
|  | 83 | 81.99 | 49.89 | 42.31 | 232 | 87.46 | 69.60 | 42.79 |
|  | 84 | 82.17 | 49.89 | 42.45 | 233 | 87.45 | 69.94 | 43.17 |
|  | 85 | 82.27 | 50.41 | 42.45 | 234 | 87.48 | 70.38 | 43.17 |
|  | 86 | 82.61 | 50.85 | 42.42 | 235 | 87.47 | 70.95 | 43.73 |
|  | 87 | 82.70 | 51.28 | 42.42 | 236 | 87.48 | 71.23 | 43.73 |
|  | 88 | 82.97 | 51.37 | 42.52 | 237 | 87.51 | 71.53 | 43.59 |
|  | 89 | 83.04 | 51.6 | 42.52 | 238 | 87.55 | 71.97 | 43.41 |
|  | 90 | 83.33 | 51.71 | 42.7 | 239 | 87.65 | 71.97 | 43.42 |
|  | 91 | 83.35 | 51.8 | 42.68 | 240 | 87.73 | 72.35 | 43.63 |
|  | 92 | 83.54 | 51.87 | 42.66 | 241 | 87.78 | 72.67 | 43.64 |
|  | 93 | 83.57 | 51.87 | 42.68 | 242 | 87.79 | 72.88 | 43.64 |
|  | 94 | 83.61 | 51.87 | 42.76 | 243 | 87.84 | 73.13 | 43.76 |
|  | 95 | 83.66 | 51.85 | 42.58 | 244 | 87.89 | 73.51 | 43.30 |
|  | 96 | 83.65 | 51.57 | 42.58 | 245 | 87.89 | 73.78 | 43.63 |
|  | 97 | 83.67 | 51.69 | 42.43 | 246 | 87.92 | 74.05 | 43.64 |
|  | 98 | 83.72 | 51.66 | 42.25 | 247 | 87.89 | 74.27 | 43.18 |
|  | 99 | 83.91 | 51.38 | 42.25 | 248 | 87.86 | 74.27 | 43.51 |
|  | 100 | 84.02 | 51.22 | 42.12 | 249 | 87.84 | 74.4 | 44.07 |
|  | 101 | 84.20 | 50.96 | 42.14 | 250 | 87.85 | 74.63 | 44.07 |
|  | 102 | 84.21 | 50.85 | 42.15 | 251 | 87.81 | 74.75 | 44.21 |
|  | 103 | 84.27 | 50.85 | 42.20 | 252 | 87.82 | 74.97 | 44.12 |
|  | 104 | 84.31 | 50.92 | 42.20 | 253 | 87.84 | 75.18 | 44.62 |
|  | 105 | 84.36 | 50.66 | 42.41 | 254 | 87.92 | 75.31 | 44.93 |
|  | 106 | 84.38 | 50.63 | 42.40 | 255 | 87.97 | 75.42 | 45.91 |
|  | 107 | 84.43 | 50.66 | 42.40 | 256 | 88.01 | 75.71 | 45.92 |
|  | 108 | 84.56 | 50.64 | 42.21 | 257 | 88.07 | 75.94 | 45.81 |
|  | 109 | 84.63 | 50.75 | 42.18 | 258 | 88.05 | 75.94 | 45.81 |
|  | 110 | 84.88 | 51.08 | 42.04 | 259 | 88.01 | 76.18 | 45.47 |
|  | 111 | 84.91 | 51.03 | 42.04 | 260 | 88.02 | 76.31 | 44.86 |
|  | 112 | 84.98 | 51.03 | 42.04 | 261 | 88.05 | 76.52 | 44.45 |
|  | 113 | 85.06 | 51.54 | 42.04 | 262 | 88.05 | 76.72 | 44.47 |
|  | 114 | 85.25 | 51.9 | 42.15 | 263 | 88.14 | 76.91 | 44.79 |
|  | 115 | 85.31 | 52.1 | 42.03 | 264 | 88.17 | 77.01 | 45.04 |
|  | 116 | 85.40 | 52.5 | 41.85 | 265 | 88.26 | 76.95 | 45.02 |
|  | 117 | 85.64 | 52.77 | 41.71 | 266 | 88.3 | 77.17 | 45.49 |
|  | 118 | 85.66 | 53.04 | 41.48 | 267 | 88.35 | 77.17 | 45.49 |
|  | 119 | 85.67 | 53.07 | 41.48 | 268 | 88.36 | 77.37 | 45.02 |
|  | 120 | 85.74 | 53.40 | 41.50 | 269 | 88.37 | 77.27 | 44.59 |
|  | 121 | 85.79 | 53.40 | 41.71 | 270 | 88.47 | 77.61 | 44.57 |
|  | 122 | 85.80 | 53.56 | 41.86 | 271 | 88.51 | 77.57 | 45.21 |
|  | 123 | 85.80 | 53.82 | 41.86 | 272 | 88.58 | 77.79 | 45.00 |
|  | 124 | 85.75 | 54.02 | 42.01 | 273 | 88.66 | 77.78 | 44.64 |
|  | 125 | 85.73 | 54.17 | 42.16 | 274 | 88.95 | 78.07 | 45.10 |
|  | 126 | 85.73 | 54.36 | 42.14 | 275 | 89.01 | 78.2 | 45.10 |
|  | 127 | 85.75 | 54.51 | 41.78 | 276 | 89.11 | 78.11 | 45.09 |
|  | 128 | 85.75 | 54.72 | 41.77 | 277 | 89.18 | 78.11 | 45.09 |
|  | 129 | 85.74 | 54.81 | 41.50 | 278 | 89.24 | 78.37 | 44.82 |
|  | 130 | 85.74 | 54.81 | 41.51 | 279 | 89.23 | 78.32 | 45.09 |
|  | 131 | 85.71 | 55.07 | 41.49 | 280 | 89.23 | 78.33 | 45.06 |
|  | 132 | 85.74 | 55.20 | 41.49 | 281 | 89.26 | 78.47 | 45.08 |
|  | 133 | 85.83 | 55.28 | 41.51 | 282 | 89.3 | 78.52 | 45.28 |
|  | 134 | 85.88 | 55.24 | 41.48 | 283 | 89.36 | 78.57 | 45.28 |
|  | 135 | 85.9 | 55.21 | 41.4 | 284 | 89.46 | 78.6 | 45.05 |
|  | 136 | 85.92 | 55.13 | 41.41 | 285 | 89.52 | 78.65 | 45.19 |
|  | 137 | 85.90 | 54.97 | 41.23 | 286 | 89.55 | 78.65 | 45.41 |
|  | 138 | 85.91 | 54.67 | 41.38 | 287 | 89.55 | 78.79 | 45.71 |
|  | 139 | 85.93 | 54.67 | 41.85 | 288 | 89.58 | 78.78 | 45.71 |
|  | 140 | 85.92 | 54.54 | 41.87 | 289 | 89.59 | 78.83 | 45.73 |
|  | 141 | 85.91 | 54.41 | 41.87 | 290 | 89.63 | 79.00 | 45.48 |
|  | 142 | 85.91 | 54.42 | 41.88 | 291 | 89.73 | 78.94 | 44.95 |
|  | 143 | 85.93 | 54.32 | 41.71 | 292 | 89.9 | 79.2 | 44.95 |
|  | 144 | 85.97 | 54.41 | 41.53 | 293 | 89.93 | 79.25 | 44.99 |
|  | 145 | 85.99 | 54.41 | 41.39 | 294 | 90.02 | 79.31 | 45.22 |
|  | 146 | 86.01 | 54.57 | 41.39 | 295 | 90.07 | 79.31 | 45.22 |
|  | 147 | 86.09 | 54.49 | 41.4 | 296 | 90.12 | 79.30 | 45.98 |
|  | 148 | 86.10 | 54.49 | 41.63 | 297 | 90.18 | 79.6 | 45.98 |
|  | 149 | 86.14 | 54.57 | 42.03 | 298 | 90.24 | 79.64 | 45.99 |
| h | 1 | 86.52 | 88.00 | 57.36 | 50 | 83.67 | 30.74 | 44.14 |
|  | 2 | 86.51 | 88.01 | 57.65 | 51 | 83.67 | 29.78 | 44.38 |
|  | 3 | 86.48 | 87.97 | 57.64 | 52 | 83.67 | 29.13 | 45.11 |
|  | 4 | 86.39 | 87.97 | 56.82 | 53 | 83.69 | 28.25 | 44.94 |
|  | 5 | 86.34 | 87.89 | 56.82 | 54 | 83.69 | 27.58 | 45.08 |
|  | 6 | 86.25 | 87.78 | 56.96 | 55 | 83.73 | 26.75 | 45.08 |
|  | 7 | 86.22 | 87.69 | 56.59 | 56 | 83.75 | 26.19 | 46.26 |
|  | 8 | 86.16 | 87.53 | 56.59 | 57 | 83.76 | 25.48 | 45.80 |
|  | 9 | 86.14 | 87.58 | 56.47 | 58 | 83.74 | 25.48 | 46.00 |
|  | 10 | 86.11 | 87.46 | 56.89 | 59 | 83.74 | 24.95 | 46.22 |
|  | 11 | 86.03 | 87.44 | 56.61 | 60 | 83.78 | 24.65 | 46.00 |
|  | 12 | 85.72 | 84.57 | 56.90 | 61 | 83.85 | 23.78 | 46.16 |
|  | 13 | 85.36 | 84.57 | 56.90 | 62 | 83.84 | 23.66 | 46.49 |
|  | 14 | 85.01 | 74.93 | 56.46 | 63 | 83.82 | 23.30 | 46.33 |
|  | 15 | 84.73 | 72.84 | 56.75 | 64 | 83.85 | 22.85 | 46.33 |
|  | 16 | 84.70 | 65.53 | 55.78 | 65 | 83.88 | 22.69 | 46.21 |
|  | 17 | 84.64 | 66.13 | 53.53 | 66 | 83.88 | 22.69 | 46.58 |
|  | 18 | 84.7 | 66.47 | 52.63 | 67 | 83.85 | 22.47 | 46.40 |
|  | 19 | 84.66 | 62.84 | 52.06 | 68 | 83.89 | 22.28 | 46.52 |
|  | 20 | 84.65 | 59.86 | 51.87 | 69 | 83.89 | 22.43 | 47.02 |
|  | 21 | 84.58 | 58.07 | 51.87 | 70 | 83.95 | 22.24 | 47.33 |
|  | 22 | 84.53 | 58.07 | 49.79 | 71 | 83.94 | 22.36 | 46.79 |
|  | 23 | 84.47 | 57.52 | 48.32 | 72 | 83.96 | 22.77 | 47.15 |
|  | 24 | 84.37 | 56.85 | 47.47 | 73 | 83.93 | 22.92 | 47.15 |
|  | 25 | 84.36 | 54.91 | 48.07 | 74 | 83.94 | 23.37 | 47.19 |
|  | 26 | 84.34 | 52.00 | 46.77 | 75 | 83.96 | 23.37 | 47.32 |
|  | 27 | 84.31 | 50.25 | 46.90 | 76 | 83.96 | 23.91 | 47.33 |
|  | 28 | 84.29 | 49.01 | 46.40 | 77 | 83.95 | 24.59 | 47.33 |
|  | 29 | 84.30 | 47.87 | 46.40 | 78 | 83.96 | 25.28 | 47.54 |
|  | 30 | 84.32 | 46.43 | 46.81 | 79 | 84.00 | 26.08 | 47.84 |
|  | 31 | 84.31 | 46.43 | 45.81 | 80 | 83.96 | 27.03 | 47.83 |
|  | 32 | 84.3 | 44.05 | 45.82 | 81 | 84.00 | 27.91 | 47.96 |
|  | 33 | 84.26 | 42.57 | 45.18 | 82 | 83.97 | 28.85 | 47.96 |
|  | 34 | 84.19 | 40.50 | 45.18 | 83 | 83.92 | 30.24 | 47.97 |
|  | 35 | 84.15 | 37.75 | 45.76 | 84 | 83.57 | 30.24 | 48.39 |
|  | 36 | 83.91 | 36.82 | 45.51 | 85 | 83.52 | 31.55 | 48.41 |
|  | 37 | 83.83 | 36.59 | 44.22 | 86 | 83.23 | 32.73 | 48.98 |
|  | 38 | 83.77 | 36.74 | 44.22 | 87 | 82.88 | 34.24 | 48.72 |
|  | 39 | 83.72 | 36.65 | 43.81 | 88 | 82.49 | 35.70 | 49.02 |
|  | 40 | 83.67 | 36.65 | 43.96 | 89 | 82.08 | 37.15 | 48.81 |
|  | 41 | 83.63 | 36.11 | 44.29 | 90 | 81.78 | 38.14 | 48.81 |
|  | 42 | 83.62 | 35.4 | 44.01 | 91 | 81.48 | 39.60 | 48.98 |
|  | 43 | 83.61 | 34.83 | 44.23 | 92 | 81.13 | 41.03 | 49.15 |
|  | 44 | 83.61 | 34.26 | 43.86 | 93 | 81.10 | 41.03 | 48.79 |
|  | 45 | 83.67 | 33.7 | 44.27 | 94 | 80.78 | 42.64 | 49.24 |
|  | 46 | 83.65 | 33.09 | 42.32 | 95 | 80.72 | 44.08 | 49.23 |
|  | 47 | 83.69 | 32.40 | 42.32 | 96 | 80.39 | 45.85 | 49.21 |
|  | 48 | 83.69 | 31.40 | 45.05 | 97 | 80.3 | 47.34 | 49.43 |
|  | 49 | 83.67 | 31.40 | 44.13 | 98 | 80.02 | 48.65 | 49.40 |
| i | 1 | 85.86 | 87.55 | 57.25 | 50 | 82.88 | 41.35 | 48.34 |
|  | 2 | 85.81 | 87.46 | 57.37 | 51 | 82.86 | 41.35 | 48.34 |
|  | 3 | 85.50 | 87.4 | 57.20 | 52 | 82.88 | 40.48 | 48.23 |
|  | 4 | 85.01 | 87.31 | 57.20 | 53 | 82.90 | 39.56 | 49.11 |
|  | 5 | 84.46 | 87.15 | 56.68 | 54 | 82.89 | 38.68 | 49.11 |
|  | 6 | 84.07 | 87.15 | 56.93 | 55 | 82.86 | 37.97 | 48.65 |
|  | 7 | 83.74 | 87.04 | 57.45 | 56 | 82.90 | 37.46 | 48.11 |
|  | 8 | 83.38 | 86.94 | 58.05 | 57 | 82.96 | 37.12 | 48.94 |
|  | 9 | 83.35 | 86.87 | 57.88 | 58 | 82.96 | 36.65 | 49.28 |
|  | 10 | 83.29 | 86.67 | 58.1 | 59 | 83.01 | 36.59 | 49.48 |
|  | 11 | 83.29 | 86.46 | 57.92 | 60 | 82.99 | 36.59 | 49.49 |
|  | 12 | 83.23 | 86.39 | 57.92 | 61 | 83.01 | 36.36 | 49.22 |
|  | 13 | 83.17 | 85.06 | 58.26 | 62 | 82.98 | 36.39 | 49.22 |
|  | 14 | 83.11 | 75.75 | 59.32 | 63 | 83.01 | 36.27 | 48.63 |
|  | 15 | 83.06 | 75.75 | 59.96 | 64 | 83.02 | 36.24 | 49.65 |
|  | 16 | 83.03 | 72.68 | 60.30 | 65 | 83.05 | 36.20 | 49.12 |
|  | 17 | 83.01 | 70.62 | 61.06 | 66 | 83.04 | 36.21 | 49.14 |
|  | 18 | 83.02 | 68.38 | 61.05 | 67 | 83.05 | 36.31 | 49.26 |
|  | 19 | 82.97 | 65.73 | 60.80 | 68 | 83.06 | 36.58 | 49.65 |
|  | 20 | 82.92 | 60.37 | 60.80 | 69 | 83.07 | 36.58 | 49.93 |
|  | 21 | 82.88 | 57.30 | 59.51 | 70 | 83.11 | 36.77 | 50.32 |
|  | 22 | 82.84 | 55.84 | 57.82 | 71 | 83.14 | 36.71 | 50.32 |
|  | 23 | 82.80 | 54.89 | 56.73 | 72 | 83.16 | 37.04 | 50.55 |
|  | 24 | 82.77 | 54.89 | 55.48 | 73 | 83.18 | 37.40 | 50.75 |
|  | 25 | 82.5 | 54.38 | 53.02 | 74 | 83.16 | 37.71 | 50.97 |
|  | 26 | 82.46 | 53.7 | 52.31 | 75 | 83.20 | 37.92 | 51.08 |
|  | 27 | 82.47 | 53.27 | 50.73 | 76 | 83.18 | 38.28 | 51.10 |
|  | 28 | 82.45 | 52.86 | 50.73 | 77 | 83.19 | 38.66 | 51.26 |
|  | 29 | 82.48 | 52.52 | 48.89 | 78 | 83.20 | 38.66 | 51.26 |
|  | 30 | 82.47 | 52.19 | 49.72 | 79 | 83.24 | 39.13 | 51.26 |
|  | 31 | 82.50 | 51.97 | 48.89 | 80 | 83.24 | 39.75 | 51.36 |
|  | 32 | 82.51 | 51.90 | 48.08 | 81 | 83.25 | 40.14 | 51.39 |
|  | 33 | 82.49 | 51.90 | 47.81 | 82 | 83.25 | 40.91 | 51.37 |
|  | 34 | 82.46 | 51.73 | 48.19 | 83 | 83.24 | 41.64 | 51.36 |
|  | 35 | 82.43 | 51.63 | 48.32 | 84 | 83.20 | 42.33 | 51.43 |
|  | 36 | 82.40 | 51.58 | 48.33 | 85 | 82.93 | 43.22 | 51.42 |
|  | 37 | 82.40 | 51.39 | 48.33 | 86 | 82.90 | 44.04 | 51.44 |
|  | 38 | 82.46 | 51.04 | 49.02 | 87 | 82.60 | 44.04 | 51.16 |
|  | 39 | 82.53 | 50.79 | 49.02 | 88 | 82.19 | 45.15 | 51.16 |
|  | 40 | 82.57 | 50.27 | 48.05 | 89 | 81.88 | 46.11 | 51.74 |
|  | 41 | 82.65 | 49.59 | 49.08 | 90 | 81.40 | 47.1 | 52.04 |
|  | 42 | 82.62 | 49.59 | 48.44 | 91 | 81.37 | 48.12 | 51.88 |
|  | 43 | 82.59 | 49.01 | 48.59 | 92 | 81.08 | 49.35 | 52.28 |
|  | 44 | 82.61 | 48.04 | 48.18 | 93 | 81.07 | 50.26 | 52.33 |
|  | 45 | 82.66 | 47.10 | 48.18 | 94 | 81.06 | 51.26 | 52.25 |
|  | 46 | 82.72 | 45.91 | 48.54 | 95 | 81.04 | 52.28 | 52.45 |
|  | 47 | 82.95 | 44.84 | 49.19 | 96 | 81.05 | 52.28 | 52.43 |
|  | 48 | 82.91 | 43.53 | 48.23 | 97 | 81.05 | 53.32 | 52.43 |
|  | 49 | 82.90 | 42.48 | 48.50 | 98 | 81.05 | 54.18 | 52.42 |

a. The thermal medium flow rate (TMFR) was 30 L/min, the scraper speed (SS) was 0 r/min, the snow completely melted time (SCMT) was 218 s; b. The TMFR was 30 L/min, the SS was 25 r/min, the SCMT time was 34 s; c. The TMFR was 30 L/min, the SS was 42 r/min, the SCMT was 10 s; d. The TMFR was 40 L/min, the SS was 0 r/min, the SCMT was 210 s; e. The TMFR was 40 L/min, the SS was 25 r/min, the SCMT was 30 s; f. The TMFR was 40 L/min, the SS was 42 r/min, the SCMT was 9 s; g. The TMFR was 60 L/min, the SS was 0 r/min, the SCMT was 206 s; h. The TMFR was 60 L/min, the SS was 25 r/min, the SCMT was 23 s; i. The TMFR was 60 L/min, the SS was 42 r/min, the SCMT was 7 s
